# Urinary Proteomics for the Early Diagnosis of Diabetic Nephropathy in Taiwanese Patients

**DOI:** 10.3390/jcm7120483

**Published:** 2018-11-26

**Authors:** Wen-Ling Liao, Chiz-Tzung Chang, Ching-Chu Chen, Wen-Jane Lee, Shih-Yi Lin, Hsin-Yi Liao, Chia-Ming Wu, Ya-Wen Chang, Chao-Jung Chen, Fuu-Jen Tsai

**Affiliations:** 1 Graduate Institute of Integrated Medicine, China Medical University, Taichung 404, Taiwan; wl0129@mail.cmu.edu.tw; 2 Center for Personalized Medicine, China Medical University Hospital, Taichung 404, Taiwan; 3 Division of Nephrology and Kidney Institute, Department of Internal Medicine, China Medical University Hospital, Taichung 404, Taiwan; d19863@mail.cmuh.org.tw (C.-T.C); Oasisbestonly@yahoo.com.tw (S.-Y.L.); 4 Institute of Clinical Medical Science, China Medical University College of Medicine, Taichung 404, Taiwan; 5 Division of Endocrinology and Metabolism, Department of Medicine, China Medical University Hospital, Taichung 404, Taiwan; chingchu@ms15.hinet.net; 6 School of Chinese Medicine, China Medical University, Taichung 404, Taiwan; 7 Department of Medical Research, Taichung Veterans General Hospital, Taichung 404 Taiwan; wjlee@vghtc.gov.tw; 8 Department of Social Work, Tunghai University, Taichung 404, Taiwan; 9 Proteomics Core Laboratory, Department of Medical Research, China Medical University Hospital, Taichung 404, Taiwan; linda198015@yahoo.com.tw; 10 Human Genetic Center, Department of Medical Research, China Medical University Hospital, China Medical University, Taichung 404, Taiwan; chiaming119@gmail.com (C.-M.W.); windy87518@gmail.com (Y.-W.C.); 11 Department of Health and Nutrition Biotechnology, Asia University, Taichung 404, Taiwan

**Keywords:** proteomics, early prediction, nephropathy

## Abstract

Diabetic nephropathy (DN) is a major complication in diabetic patients. Microalbuminuria testing is used to identify renal disease; however, its predictive value is questionable. We aimed to identify urinary biomarkers to early diagnosis nephropathy before identifiable alternations in kidney function or urine albumin excretion occurs. Proteomic approaches were used to identify potential urinary biomarkers and enzyme-linked immunosorbent assay was performed to verify the results. The data identified haptoglobin (HPT) and α-1-microglobulin/bikunin precursor (AMBP) as two biomarkers with the highest ability to distinguish between healthy individuals and patients with nephropathy, and between diabetic patients with and without DN. Further, the HPT-to-creatinine ratio (HCR) was evaluated as an independent predictor of early renal functional decline (ERFD) in a cohort with an average follow-up of 4.2 years. The area under the curve (AUC) value for ERFD prediction was significantly improved when the HCR biomarker was included in the model with albumin to creatinine ratio (ACR) and baseline characteristics (AUC values were 0.803 and 0.759 for HCR and ACR, respectively; *p* value was 0.0423 for difference between models). In conclusion, our results suggest that HCR represents an early indicator of nephropathy, and a marker related to ERFD among diabetic patients in Taiwan.

## 1. Introduction

Diabetic nephropathy (DN) is a major complication of diabetes. It is estimated that approximately 20–40% of type 2 diabetes (T2D) patients will develop renal disease [1,2]. Moreover, DN is the leading cause of end-stage renal disease (ESRD) worldwide [3]. Analysis of the international statistics collected in the US Renal Data System indicates that Taiwan has the highest incidence and the second highest prevalence of ESRD worldwide [4,5]. To date, the most useful approach to identify renal disease in nonproteinuric diabetic patients is microalbuminuria (MA) testing. Patients with MA often progress to macroalbuminuria and ESRD. However, the prognostic value of MA as a biomarker of disease progression is questionable, as patients with MA may exhibit the preservation of renal function [6]. In addition, some studies indicate that a subset of diabetic patients with normal albuminuria may develop nephropathy [7], suggesting that patients may have declined renal function before the onset of MA. Moreover, MA is associated with high blood pressure [5], and it represents a potential risk factor for cardiovascular events in individuals with or without diabetes [8]. Therefore, the identification of reliable biomarkers for the early prediction of DN and the development of effective treatment to reduce the incidence of ESRD is necessary.

Proteins are functional executors of biological mechanisms, and they have been utilized as prognostic biomarkers for a number of diseases such as DN, cardiovascular disease, and cancer [9,10,11,12]. With the development of protein separation techniques and mass spectrometry, liquid chromatography tandem mass spectrometry (LC-MS/MS)-based proteomics has been widely applied to the analysis of clinical samples for the elucidation of protein changes in complex mixtures. In the case of nephropathy, proteomic studies have identified several urinary biomarkers, such as alpha 1 antitrypsin [13], extracellular glutathione peroxidase [14], and apolipoprotein A-1 [15], to be associated with renal functional decline.

In the present investigation, we used isobaric tags for relative and absolute quantification (iTRAQ) and label-free proteomics to identify potential urinary protein markers that are associated with nephropathy, which were verified by an enzyme-linked immunosorbent assay (ELISA) in a cross-sectional population study. We additionally performed a nested case-control study to evaluate the ability of one urinary protein, haptoglobin (HPT), on the discrimination of early renal functional decline (ERFD) among T2D patients in Taiwan.

## 2. Materials and Methods

### 2.1. Study Population

#### 2.1.1. Proteomic Discovery Phase

Four groups of participants, including healthy individuals (*n* = 35), patients with micro- or macroalbuminuria due to nondiabetic disease (WDM-NP; albumin-to-creatinine ratio (ACR) ≥ 30 mg/g; *n* = 25), diabetic patients without micro- or macro-albuminuria (DM-WNP; ACR < 30 mg/g; *n* = 24), and diabetic patients with microalbuminuria (DM-NP; 30 mg/g ≤ ACR < 300 mg/g; *n* = 16) were included in the proteomic discovery phase. The serum creatinine levels of all subjects were < 1.3 mg/dL. Random urine samples were collected. The urine from these patients was pooled according to four groups for proteomic analysis, using iTRAQ labeling and a label-free quantitative proteomics approach.

#### 2.1.2. Verification Phase

A cross-sectional study design was applied for the verification of eight potential protein biomarkers using ELISA. A total of 208 participants, including 34 healthy individuals and 30 WDM-NP, 89 DM-WNP, and 55 DM-NP patients were recruited in this phase; all subjects had normal albuminuria or MA (ACR < 300 mg/g). Subjects in the verification phase were different from those in the discovery phase.

#### 2.1.3. Evaluation of the Role of HPT on ERFD

A nested case-control study design was used for the evaluation of the role of one potential protein biomarker, HPT, on ERFD using ELISA. Urine samples from 305 T2D patients were obtained from the Taiwan Biobank [16]. The renal function of all patients was characterized by an estimated glomerular filtration rate (eGFR) > 60 mL/min per 1.73 m^2^, and the ACR < 300 mg/g at the time of enrollment. Among the patients, 59 had ERFD, and 246 did not have ERFD during the follow-up period (4.2 years on average). ERFD was defined as a decline of more than 3.3% per year [17]. The Modified Diet in Renal Disease (MDRD) equation [18] was used to estimate the glomerular filtration rate (GFR). 

**Figure 1:**
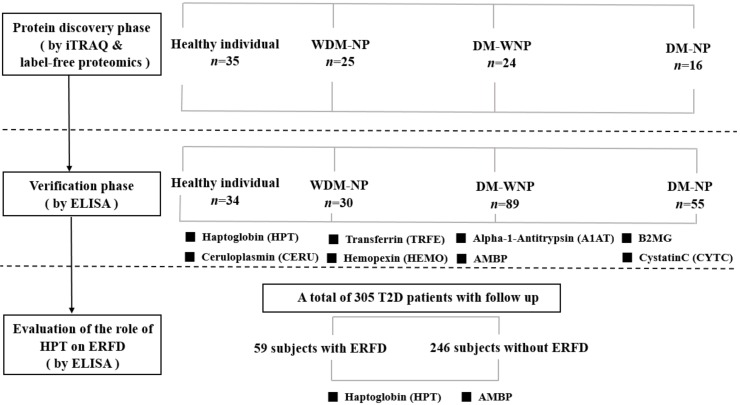
Flowchart of the three phases of the study. Abbreviation: iTRAQ: isobaric tags for relative and absolute quantification; ELISA: enzyme-linked immunosorbent assay; WDM-NP: patients with micro- or macroalbuminuria due to nondiabetic disease; DM-WNP: diabetic patients without micro- or macro-albuminuria; DM-NP: diabetic patients with microalbuminuria; ERFD, early renal function decline; AMBP: α-1-microglobulin/bikunin precursor; T2D: type 2 diabetes; B2MG: β-2-microglobulin.

All subjects in three phases of the study gave their informed consent for inclusion before they participated in the study. The study was conducted in accordance with the Declaration of Helsinki, and the protocols were approved by the China Medical University Hospital Institutional Review Board (DMR101-IRB-2-267 and CMUH103-REC2-071). The flowchart for the investigation is shown in Figure 1.

### 2.2. Method for the Proteomic Discovery Phase

#### 2.2.1. Urine Sample Preparation for Proteomic Analysis

Urine samples were centrifuged at 3000 × *g* for 15 min at 4 °C, and the supernatant was collected, supplemented with protease inhibitor cocktail (Complete Mini; Roche Diagnostics, Indianapolis, IN, USA), and stored at −80 °C. Albumin was removed from urine using the ProteoPrep Immunoaffinity kit (Sigma-Aldrich, St. Louis, MO, USA), and urine proteins were precipitated using the 2D clean-up kit (General Electric (GE) Healthcare Life Sciences, Piscataway, NJ, USA), which also removed interfering substances. The purified protein pellet was obtained by centrifugation, and then re-suspended in triethyl ammonium bicarbonate (TEABC) buffer. Protein quantification was performed by the Bradford assays.

#### 2.2.2. iTRAQ Labeling

iTRAQ labeling was performed according to the manufacturer’s instructions using the iTRAQ 4plex kit (Applied Biosystems-SCIEX, Foster, CA, USA). Briefly, 50 μg of protein from the pooled samples of each group (healthy, WDM-NP, DM-WNP, and DM-NP) were dissolved in 20 μL of TEABC buffer, followed by the addition of 2 μL of reducing agent and cysteine-blocking agent. Protein samples were digested with trypsin overnight; peptides were then labeled with iTRAQ reagents for 2 h at 25 °C and quenched with Milli-Q water. The iTRAQ-labeled peptide samples were combined and analyzed by nano-LC-MS/MS.

#### 2.2.3. Nano-LC-MS/MS

Nano-LC-MS/MS was performed using a nanoflow ultra-high performance liquid chromatography system (UltiMate 3000 RSLCnano system, Dionex, Amsterdam, Netherlands) coupled to a hybrid Q-TOF mass spectrometer (maXis impact, Bruker Daltonics, Billerica, MA, USA). The labeled peptides were injected into a C18 LC column (Atlantis T3 C18, 5 µm, 2.1 × 150 mm) and eluted with a linear gradient of mobile phase A (water containing 0.1% (*v/v*) formic acid) and mobile phase B (99.9% acetonitrile and 0.1% (*v/v*) formic acid) applied at a flow rate of 0.3 μL/min. Gradient conditions were as follows: 1% to 40% (*v/v*) B for 80 min, and then to 99% B for 5 min; hold at 99% B for 4 min at the same flow rate, then return to the starting conditions and column re-equilibration with 1% B (*v/v*) for 10 min. For MS detection, peptides with a charge of 2+, 3+, or 4+ and intensities of greater than 500 counts were selected for data-dependent acquisition, which was set to one full MS scan (400–2000 m/z) with 1 Hz, and it was switched to 16 product ion scans (50–2000 m/z) with 20 Hz.

#### 2.2.4. Label-Free Quantitative Proteomics

Label-free quantitative proteomics was performed using the DataAnalysis 4.1, ProfileAnalysis 2.1, and ProteinScape 3.0 software packages from Bruker Daltonics (Bremen, Germany). Briefly, 10 μg of protein from each of the four pooled samples was digested and analyzed by nano-LC-MS/MS with replicated runs (*n* = 4) for the quantification of peptide ions and by nano-LC-MS/MS to acquire MS/MS spectra for protein identification. Peak intensities of peptide ions from each nano-LC-MS run were processed by the “Find Molecular Features” (FMF) algorithm in DataAnalysis 4.1, with the following parameters: a minimum retention time of 20 min, a maximum retention time of 90 min, a minimum compound length of eight spectra, and a smoothing width of 4. The intensity and elution time of each peptide ion were recorded as a quantitative “molecular feature” and used to construct a feature map. Feature abundances on different maps were compared using ProfileAnalysis 2.1 with a *t*-test to reveal the relative changes between peptide ions. The results of the *t*-test comparison and the quantification of all peptide ions were transferred to ProteinScape to obtain quantitative and qualitative data on the protein composition in each group.

#### 2.2.5. Protein Database Search

Nano-LC-MS/MS spectra were deisotoped, centroided, and converted to xml files using DataAnalysis 4.1. To identify the proteins, the obtained mass spectra were compared to those in the SwissProt database (release 51.0) using the MASCOT search algorithm (version 2.3). The search parameters for MASCOT were as follows: peptide mass tolerance, 50 ppm; MS/MS mass tolerance, 0.07 Da; taxonomy, human; enzyme, trypsin; fixed modification, iTRAQ4plex (Lys/N-term), and methylthio (C) for the iTRAQ experiment, and carbamidomethyl (Cys) for the label-free experiment; variable modification, oxidation (Met), and deamidation (Asn/Gln). The identification of peptides was performed based on a MASCOT individual ion score of higher than 25.

### 2.3. ELISA 

Urinary protein candidates were quantitatively analyzed using sandwich ELISA (USCN Life Science Inc., Wuhan, China). Briefly, 100 μL/well of urine samples or standards was added to microtiter plates coated with the specific protein; then, the biotin-conjugated antibodies specific to a protein candidate was added as secondary antibodies for detection. The assay was performed according to the manufacturer’s instructions. The optical density was measured at 405 nm, and the concentration of protein candidates in the samples was calculated based on the standard curve.

### 2.4. Statistical Analysis

Continuous data are presented as the mean with the standard deviation, or the median with the interquartile range, and categorical data are presented as proportions. *T*-tests or Mann-Whitney *U* tests were used to compare mean/median values of continuous variables, and chi-squared tests or Fisher exact tests were used to compare the frequencies of categorical variables between two groups. The Kruskal–Wallis test with Dunn’s method for post hoc testing was used to compare differences among the median values in the groups. Odds ratios (ORs) and 95% confidence intervals (CIs) of variables were estimated using logistic regression to examine the independent association between urinary proteins and the ERFD end point. The potential variables in the logistic models were selected by the backward selection method. Receiver operating characteristic (ROC) curve analysis was applied to assess the predictive accuracy, and the area under the curve (AUC) was used to assess the discriminatory ability of the predictive model. The statistical significance of the difference between the AUC values was determined using Z statistics [19]. In the models, patients were classified according to the ACR (low, <30 mg/g, and high, 30–300 mg/g) based on the Kidney Disease: Improving Global Outcomes (KDIGO) guidelines [20], and the HPT-to-creatinine ratio (HCR) (low, <10.38 ng/mg, and high, ≥10.38 ng/mg) based on the optimal cut-off point from the AUC. All statistical analyses were conducted using the IBM SPSS statistical software, version 22.0 (IBM Corp., Armonk, NY, USA), and *p* values less than 0.05 (two-sided) were considered significant.

## 3. Results

### 3.1. Proteomic Discovery Phase 

Urine proteins from four pooled group samples (healthy individuals (*n* = 35), and patients with micro- or macro-albuminuria due to nondiabetic disease (WDM-NP, *n* = 25), diabetic patients without micro- or macro-albuminuria (DM-WNP, *n* = 24), and diabetic patients with microalbuminuria (DM-NP, *n* = 30)) were compared, based on iTRAQ labeling and nano-LC-MS/MS analysis. In total, 341 and 396 proteins were identified by iTRAQ and label-free proteomics analyses, respectively (see Supplementary Table S1-1 and Table S1-2 online). Among them, 17 were upregulated (Supplementary Table S2-1) and 20 downregulated (Supplementary Table S2-2) according to iTRAQ results, whereas 49 were upregulated (Supplementary Table S2-3) and 84 downregulated (Supplementary Table S2-4) according to label-free proteomics (a fold change of more than 1.5 for WDN-NP vs healthy or DM-NP vs DM-WNP). Seven proteins (serotransferrin, ceruloplasmin, hemopexin, alpha-1-antitrypsin, beta-2-microglobulin, HPT, and α-1-microglobulin/bikunin precursor (AMBP)) were selected for further ELISA validation as they were increased both in the WDM-NP group and the DM-NP group relative to healthy individuals and the DM-WNP group, respectively (Table 1). 

### 3.2. Verification Phase

**Figure 2:**
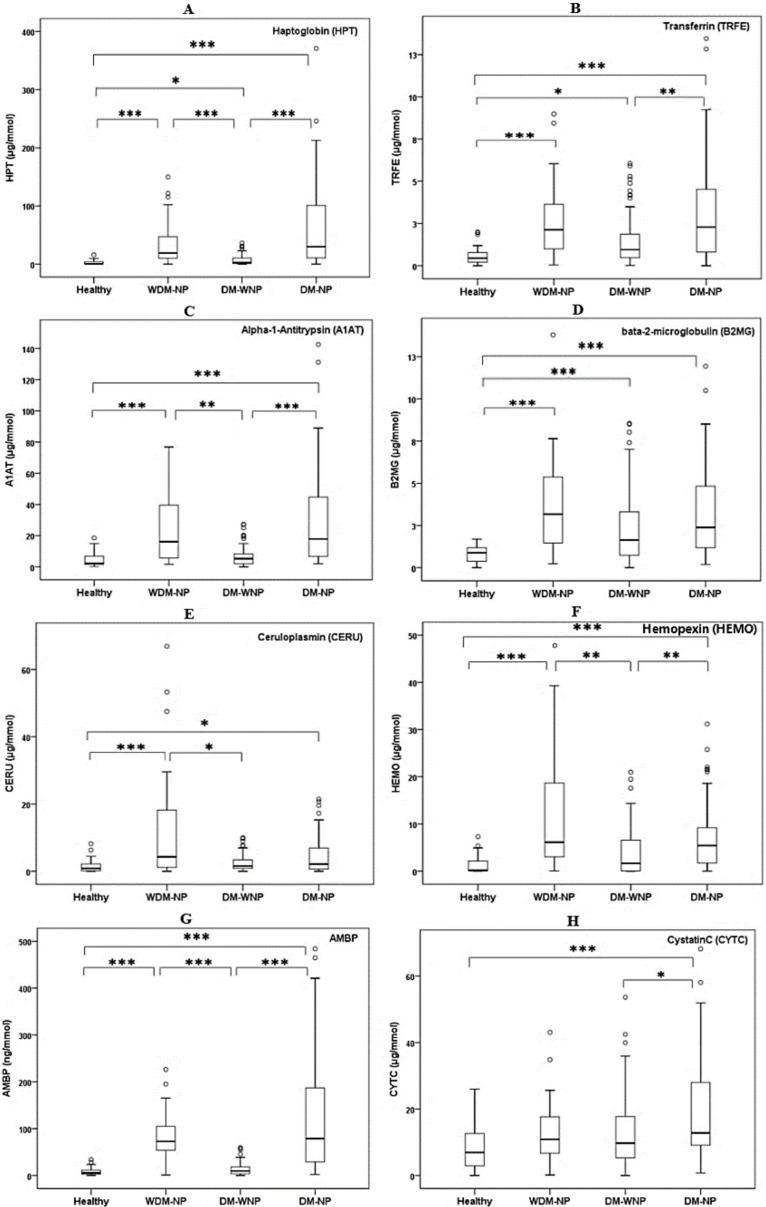
ELISA results for eight urinary protein biomarkers (**A**. HPT; **B**. TRFE; **C**. A1AT; **D**. B2MG; **E**. CERU; **F**. HEMO; **G**. AMBP; **H**. CYTC) in healthy individuals (*n* = 34), patients with micro- or macro-albuminuria due to nondiabetic disease (WDM-NP, *n* = 30), diabetic patients without micro- or macro-albuminuria (DM-WNP, *n* = 89), and diabetic patients with microalbuminuria (DM-NP, *n* = 55) patients in the initial verification phase (cross-sectional study design). * represent *p* value less than 0.05; ** represent *p* value less than 0.01; ***represent *p* value less than 0.001.

**Figure 3:**
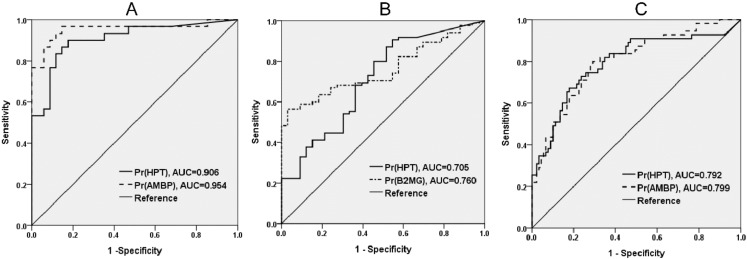
Receiver operating characteristic (ROC) analysis and area under the curve (AUC) of two protein biomarkers with the highest discriminative ability in the initial verification phase (case control study design). **A**. Healthy individuals vs. patients with micro- or macro-albuminuria due to nondiabetic disease (WDM-NP); **B**. Healthy individuals vs. diabetic patients without micro- or macro-albuminuria (DM-WNP); **C**. DM-WNP vs. diabetic patients with microalbuminuria (DM-NP).

A cross-sectional study design was used for the verification of eight potential protein biomarkers, including seven protein markers selected in the proteomic discovery phase, and one clinical marker of kidney injury, cystatin C. The demographic and clinical characteristics of the study population recruited for the verification of urinary protein markers are presented in Table S3. Seven proteins identified during the proteomic discovery phase and one clinical marker of kidney injury, cystatin C, were tested by ELISA (Figure 2). To investigate the ability of the biomarkers to distinguish between individuals from proteinuria and non-proteinuria groups, ROC and AUC analyses were performed (see Supplementary Figure S1 online). The ROC analysis showed that HPT and AMBP had the two highest AUCs to distinguish WDM-NP patients from healthy individuals (AUC = 0.906 and 0.954 for HPT and AMBP, respectively) and DM-NP from DM-WNP patients (AUC = 0.792 and 0.799 for HPT and AMBP, respectively) (Figure 3).

### 3.3. Evaluation of the Role of HPT on ERFD

Next, we evaluated HPT in a cohort of 305 T2D patients, including 59 who had ERFD during follow-up. Patients with ERFD were of similar age and diabetic duration, and had comparable body mass indices, hemoglobin A1c (HbA1c), creatinine levels, ACR, and diastolic blood pressure. Compared with patients without ERFD, those with ERFD had shorter follow-up times (4.57 ± 1.52 versus 3.37 ± 1.61 years; *p* < 0.001), and higher eGFR (100.54 ± 22.39 versus 113.36 ± 25.62; *p* < 0.001), HCR (17.98 ± 64.80 versus 42.97 ± 79.11; *p* = 0.028) and systolic blood pressures (SBP) (121.25 ± 17.20 versus 127.63 ± 18.80; *p* = 0.013) (Table 2).

To examine the independent association between urinary proteins and ERFD, we used multivariate logistic regression analysis (Table 3). A comparison of patients with ACR of 30–300 mg/g and those with an ACR of < 30 mg/g yielded an OR of 1.38 (95% CI: 0.65–2.94) for developing ERFD, whereas a comparison of patients with a high and low HCR yielded an OR of 4.47 (95% CI: 2.20–9.09) after adjustment for follow-up time, SBP, and eGFR levels. The AUC value for ERFD prediction by the HCR was 0.804, and that by ACR was 0.759 (*p* = 0.0398 between the models). Furthermore, when we combined HCR and ACR into the models, the AUC value was 0.803. A difference in the AUC value between the model with ACR and the model with ACR and HCR was observed (*p* = 0.0423). No difference in AUC value between the model with HCR and the model with ACR and HCR was found. 

## 4. Discussion

In the present study, we identified urinary HPT as a candidate biomarker that is associated with nephropathy, and verified its discriminatory ability in a population of 208 individuals comprising diabetic and nondiabetic patients. The ability of HCR to predict ERFD was evaluated in 305 T2D patients, with an average follow-up of 4.2 years. Although the HCR and ACR were correlated (*r*^2^_pearson_ = 0.085, *p* < 0.001, see Supplementary Figure S2 online), HCR was identified as an independent predictor of ERFD among T2D patients who had not yet manifested significant kidney disease, as shown in data in logistic models. The HCR had higher sensitivity (28.8%), specificity (96.5%), and positive predictive value (68.0%) than the ACR (18.6%, 97.4%, and 64.7%, respectively) to predict ERFD in our cohort population. These data indicate that the HCR may be a more sensitive biomarker than the ACR, and it may be used as a confirmatory test for the early detection of decline in renal function. Moreover, when we limited our population to T2D patients with normal albumin levels in urine, patients with higher urinary HCR had a higher risk of developing ERFD, indicating that the HCR may be a valuable clinical tool for predicting nephropathy before the appearance of urinary albumin (when limited to T2D patients with ACR < 30 mg/g (*n* = 226), the O.R. for developing ERFD was 4.15 (1.88, 9.16) when comparing individuals with higher HCR with those with lower HCR).

HPT has been identified as a biomarker for several clinical outcomes, including acute allograft rejection [11], DN [21,22], chronic renal insufficiency [23], and glomerulonephritis. HPT is a heavily glycosylated alpha-2 sialoglycoprotein with a net negative charge [24]. This protein was shown to bind hemoglobin and to prevent the loss of iron through the kidneys and oxidative damage to nephrons [25,26], as the HPT–hemoglobin complex cannot be filtered at the glomerulus, owing to its large size and negative charge. Increased concentrations of HPT may be a response to renal tubular injury or oxidative stress. Moreover, HPT, similar to albumin, is a marker of increased glomerular permeability, as serum HPT can leak into urine when the glomerular permeability reaches a certain level [27]. Recently, HPT has been identified as a biomarker of kidney damage in the Chinese population. Studies have shown that urinary HPT can predict ERFD among T2D patients with chronic kidney disease (CKD) of stages 1 and 2 [27], or even in the CKD of stage 3 [28]. In a 5.3-year cohort study, Yang et al. [23] also identified HPT as a novel biomarker that complements urine albumin in predicting chronic renal insufficiency (< 60 mL/min per 1.73 m^2^) in T2D patients with eGFR > 80 mL/min per 1.73 m^2^. In the present study, we validated the ability of HPT to predict ERFD among T2D patients with CKD stages 1 and 2 among the Taiwanese population. 

Our results showed that AMBP had the highest AUC value in the ELISA verification phase, including four cross-sectional groups. AMBP could pass through the glomerulus and subsequently be re-absorbed by the tubular tissues. AMBP is a stable urinary protein that has been used as a clinical parameter for the detection and differentiation of proteinuria. High-urine AMBP levels indicate proximal tubular dysfunction, and they are associated with interstitial fibrosis, tubular atrophy, and inflammation [11,21]. A previous study using ProteinChip (H50 chip) analysis has shown that urinary AMBP is increased in DN [22]. More, the increase of AMBP level in urinary exosomes from individuals with advanced stages of DN (CKD stages III–V, with high urinary albumin), compared with healthy subjects, was revealed by label-free comparative analysis [29]. These results suggested that AMBP in total urine or urinary exosomes could be a potential biomarker for DN. However, this marker was not sufficient for the early prediction of ERFD, based on our preliminary results from a nested case-control study (see Supplementary Table S4 online). The possible reasons for the lack of significant association between AMBP levels and ERFD may be the small differences of AMBP level between groups, and the small sample size. The predictive potential of AMBP should be determined in a larger patient population. Alternatively, AMBP may not increase at early stages of ERFD. 

Our investigation had several strengths. To the best of our knowledge, the present work describes the largest cohort study, in which samples were collected nation-wide by the Taiwan Biobank. The HCR was validated as a biomarker that outperformed the ACR in predicting renal decline progression among T2D patients in Taiwan. Moreover, the result from our verification phase (cross-sectional study) indicates that the HCR may serve as a biomarker of nephropathy not only in diabetic patients but also in nondiabetic individuals. Finally, the HCR was found to be an independent predictor of ERFD in T2D patients, even before the appearance of urinary albumin. 

However, there were also some limitations in this study. First, the study population was limited to patients with an ACR of less than 300 mg/g, and none of the patients had stage 3 CKD (eGFR level between 30 and 59 mL/min per 1.73 m^2^); therefore, we did not test whether HPT could predict ERFD among T2D patients with stage 3 CKD, which has been demonstrated by Liu et al. [28]. In addition, we did not collect information on the use of insulin or other drugs (such as renin-angiotensin system antagonists) that may alter renal function. However, according to our data, eGFR and SBP/diastolic blood pressure (DBP) values were similar between individuals with lower and higher HCRs at baseline. Third, the HPT biomarker was validated in a Taiwanese population only. Further validation steps should be performed in more diverse populations. Moreover, previous studies have shown that several protein and metabolic urinary markers such as CD59 and alpha‑1 antitrypsin were associated with albuminuria and with the capacity to predict the progression to high albuminuria [30]. Therefore, this may be another direction for investigating the potential urinary protein markers in a cohort in which subjects are with normoalbuminuric at baseline, and will develop albuminuria in the future.

## 5. Conclusions

In summary, urinary HPT and AMBP were identified as indicators of nephropathy, and HPT was confirmed as a biomarker related to the rapid decline in renal function among Taiwanese T2D patients who had not yet manifested significant kidney disease.

## Figures and Tables

**Table 1:** The selected protein biomarkers identified by iTRAQ and label free proteomic approaches for ELISA assay.

Accession/ Protein	Proteomic Approach	WDM-NP: Healthy (Ratio)	Number of Quantitative Peptides	CV (%)	DM-WNP: Healthy (Ratio)	Number of Quantitative Peptides	CV (%)	DM-NP: DM-WNP (Ratio)	Number of Quantitative Peptides	CV (%)
TRFE_ HUMAN										
Serotransferrin M.W.: 77.0 kDa	iTRAQ	4.71	36	121.14	1.22	36	67.65	2.56	47	66.06
	label-free	15.36	31	75.14	1.68	30	67.45	3.67	39	44.00
CERU_HUMAN										
Ceruloplasmin M.W.: 122.1 kDa	iTRAQ	1.87	4	28.26	1.27	4	25.30	1.72	5	29.68
	label-free	5.18	15	46.62	2.08	11	36.90	1.98	14.00	18.50
HEMO_HUMAN										
Hemopexin M.W.: 51.6 kDa	iTRAQ	1.62	1	N.D.	1.08	1	N.D.	N.D.	N.D.	N.D.
	label-free	2.08	12	28.91	0.99	12	63.44	3.08	11.00	21.52
A1AT_HUMAN										
𝛼-1-antitrypsin M.W.: 46.7 kDa	iTRAQ	7.29	20	95.11	0.67	14	53.67	3.05	16	55.02
	label-free	10.49	16	42.81	0.54	13	63.87	5.18	14.00	42.85
B2MG_HUMAN										
β-2-microglobulin M.W.: 13.7 kDa	iTRAQ	1.50	4	90.85	0.95	4	41.36	1.42	4	17.18
	label-free	2.24	2	7.20	0.36	1	N.D.	1.62	2.00	19.02
HPT_HUMAN										
Haptoglobin M.W.: 45.2 kDa	iTRAQ	3.42	10	67.54	1.11	9	39.42	1.30	7	18.55
	label-free	9.56	12	87.79	1.79	13	65.41	1.48	13.00	27.33
AMBP_HUMAN										
Protein AMBP M.W.: 39.0 kDa	iTRAQ	1.40	7	187.03	0.41	7	79.48	5.75	6	206.59
	label-free	2.20	20	138.75	0.54	15	88.25	3.20	19.00	317.40

Abbreviation: iTRAQ: isobaric tags for relative and absolute quantification; ELISA: enzyme-linked immunosorbent assay; WDM-NP: patients with micro- or macro-albuminuria due to nondiabetic disease; DM-WNP: diabetic patients without micro- or macro-albuminuria; DM-NP: diabetic patients with microalbuminuria; TRFE: serotransferrin; M.W.: molecular weight; N.D.: not detected; CERU: Ceruloplasmin; HEMO: Hemopexin; A1AT: 𝛼-1-antitrypsin; B2MG: β-2-microglobulin; HPT: haptoglobin; AMBP: α-1-microglobulin/bikunin precursor; CV: Coefficient of variation.

**Table 2:** Demographic and clinical characteristics of type 2 diabetes patients for evaluation of the role of haptoglobin on early renal function decline (ERFD).

	Non-ERFD (*n* = 230)	ERFD (*n* = 59)	*p*-Value
Age (years)	55.06 (8.40)	56.22 (7.94)	0.340
Diabetes duration (years)	6.85 (6.92)	8.11 (5.79)	0.324
Follow-up time (years)	4.57 (1.52)	3.37 (1.61)	<0.001 *
HbA1c (%)	7.64 (1.52)	7.91 (1.90)	0.309
Creatinine (mg/dL)	0.77 (0.18)	0.72 (0.19)	0.053
eGFR (mL/min per 1.73 m^2^)	100.54 (22.39)	113.36 (25.62)	<0.001 *
ACR (mg/g)	25.68 (41.08)	32.35 (51.24)	0.293
HCR (ng/mg)	17.98 (64.80)	42.97 (79.11)	0.028 *
BMI (kg/m^2^)	26.15 (3.71)	26.20 (4.02)	0.927
SBP (mmHg)	121.25 (17.20)	127.63 (18.80)	0.013 *
DBP (mmHg)	72.70 (10.77)	72.58 (10.59)	0.939

Abbreviation: T2D: type 2 diabetes; ERFD: early renal function decline; HbA1c: hemoglobin A1c; eGFR: estimated glomerular filtration rate; ACR: albumin to creatinine ratio; HCR: haptoglobin to creatinine ratio; BMI: body mass index; SBP: systolic blood pressure; DBP: diastolic blood pressure. * represent *p* value less than 0.05.

**Table 3:** Odds ratios with 95% confidence intervals (CI) and area under the curve (AUC) values for ERFD prediction in different logistic regression models.

	Model 1 ACR	Model 2 HCR	Model 3 HCR and ACR
Follow-up time	0.64 (0.53, 0.78)	0.58 (0.47, 0.71)	0.58 (0.47, 0.71)
SBP	1.02 (1.00, 1.04)	1.02 (1.00, 1.04)	1.02 (1.00, 1.04)
eGFR	1.02 (1.01, 1.04)	1.02 (1.01, 1.04)	1.02 (1.01, 1.04)
Lower conc. *	1.00		1.00
Upper conc. *	1.38 (0.65, 2.94)		1.03 (0.47, 2.24)
Lower conc. ^#^		1.00	1.00
Upper conc. ^#^		4.47 (2.20, 9.09)	4.45 (2.17, 9.16)
AUC value	0.759	0.804	0.803

Abbreviation: SBP: systolic blood pressure; eGFR, estimated glomerular filtration rate; conc., concentration; AUC: area under the curve; ACR: albumin to creatinine ratio; HCR: haptoglobin to creatinine ratio. * For ACR, subjects with ACR < 30 mg/g were defined as the lower group and subjects with ACR 30–300 mg/g were defined as the upper group. ^#^ For HCR, subjects with HCR < 10.38 ng/mg were defined as the lower group and subjects with HCR ≥ 10.38 ng/mg were defined as the upper group.

## Supplementary Materials

The following are available online at http://www.mdpi.com/2077-0383/7/12/483/s1, Table S1-1: Proteins identified by isobaric tags for relative and absolute quantification. Table S1-2: Proteins identified by label-free quantitative proteomics; Table S2-1: Seventeen proteins were up-regulated according to the iTRAQ proteomics results; Table S2-2: Twenty proteins were down-regulated according to the iTRAQ proteomics results; Table S2-3: Forty-nine proteins were up-regulated according to the results of label-free quantitative proteomics; Table S2-4: Eighty-four proteins were down-regulated according to the results of label-free quantitative proteomics; Table S3: Demographic and clinical characteristics of study participants recruited for verification of urinary protein markers; Table S4: Preliminary results from nested case-control study to validate two potential urinary biomarkers in verification phase; Figure S1: ROC analysis of eight protein biomarkers in the initial verification phase (cross-sectional study design); Figure S2: Correlation between HCR and ACR.
