# Supplementary material for: Urinary Proteomics for the Early Diagnosis of Diabetic Nephropathy in Taiwanese Patients
Source: J Clin Med. 2018 Nov 26;7(12):483. doi: 10.3390/jcm7120483 (PMC6306863; doi:10.3390/jcm7120483)
Supplement: Supplementary file 1 [file jcm-07-00483-s001.pdf]

Urinary Proteomics for the Early Diagnosis of Diabetic Nephropathy in Taiwanese Patients

Authors: Wen-Ling Liao[1,2], Chiz-Tzung Chang[3,4], Ching-Chu Chen[5,6], Wen-Jane Lee[7,8], Shih-Yi Lin[3,4], Hsin-Yi Liao[9], Chia-Ming Wu[10], Ya-Wen Chang[10], Chao-Jung Chen[1,9,+,*], Fuu-Jen Tsai[6,10,11,+,*]

1 Graduate Institute of Integrated Medicine, China Medical University, Taichung, 404, Taiwan

2 Center for Personalized Medicine, China Medical University Hospital, Taichung, 404, Taiwan

3 Division of Nephrology and Kidney Institute, Department of Internal Medicine, China Medical University Hospital, Taichung, 404, Taiwan

4 Institute of Clinical Medical Science, China Medical University College of Medicine, Taichung, 404, Taiwan

5 Division of Endocrinology and Metabolism, Department of Medicine, China Medical University Hospital, Taichung, 404, Taiwan

6 School of Chinese Medicine, China Medical University, Taichung, 404, Taiwan

7 Department of Medical Research, Taichung Veterans General Hospital, Taichung, 404, Taiwan

8 Department of Social Work, Tunghai University, Taichung, 404, Taiwan

9 Proteomics Core Laboratory, Department of Medical Research, China Medical University Hospital, Taichung, 404, Taiwan

10 Human Genetic Center, Department of Medical Research, China Medical University Hospital, China Medical University, Taichung, 404, Taiwan

11 Department of Health and Nutrition Biotechnology, Asia University, Taichung, 404, Taiwan

[+] Fuu-Jen Tsai and Chao-Jung Chen contributed equally to this work.

Correspondence:

Fuu-Jen Tsai, MD, PhD and Chao-Jung Chen, PhD

FJ Tsai: Genetic Center, China Medical University Hospital, No.2 Yuh-Der Road, 404 Taichung, Taiwan; Telephone: 886-4-22062121 Ext. 2041; Fax: 886-4-22033295; E-mail: d0704@mail.cmuh.org.tw

CJ Chen: Graduate Institute of Integrated Medicine, China Medical University, No.91, Hsueh-Shih Road, 404, Taichung, Taiwan; Telephone: 886-4-22053366 Ext. 1542; Fax: 886-4-22033295; E-mail: cjchen@mail.cmu.edu.tw

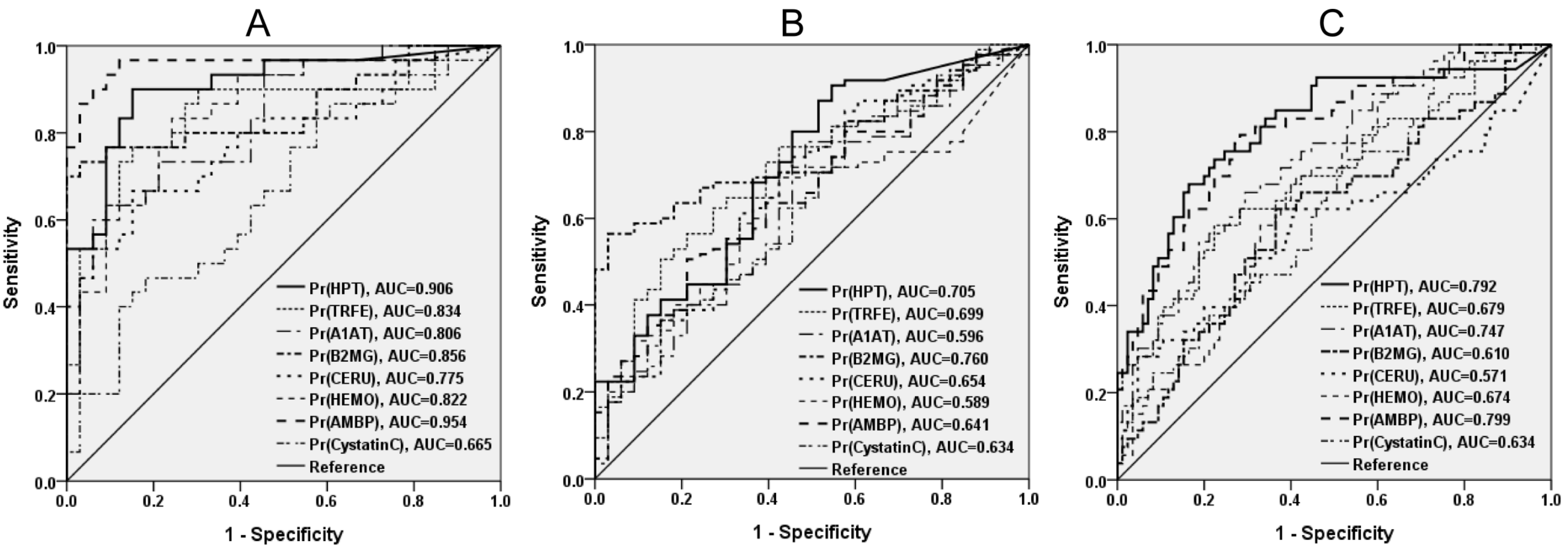

Figure S1. ROC analysis of eight protein biomarkers in the initial verification phase (cross-sectional study design). A. Healthy individuals vs. nondiabetic patients with nephropathy (WDM-NP); B. Healthy individuals vs. diabetic subjects without nephropathy (DM-WNP); C. DM-WNP vs. diabetic subjects with nephropathy (DM-NP). Abbreviation: A1AT, alpha-1-antitrypsin; AMBP, α-1-microglobulin/bikunin precursor; B2MG, beta-2-microglobulin; CERU, ceruloplasmin; HPT, haptoglobin; HEMO, hemopexin; TRFE, serotransferrin; AUC, area under curve; Pr, probability.

Table S1-1. Proteins identified by isobaric tags for relative and absolute quantification.

							(WDM-NP: Healthy)			(DM-WNP: Healthy)			(DM-NP: DM-WNP Healthy)		
NO	Accession	Protein	MW (kDa)	Scores	Qualitative peptides	SC (%)	iTRAQ Median	Quantitative peptides	CV (%)	iTRAQ Median	Quantitative peptides	CV (%)	iTRAQ Median	Quantitative peptides	CV (%)
1	A1AG1	Alpha-1-acid glycoprotein 1	23.5	801.5	15	31.8	7.94	10	86.17	0.73	10	50.55	4	9	47.17
2	A1AG2	Alpha-1-acid glycoprotein 2	23.6	356.7	9	24.9	3.27	5	156.47	0.63	5	30.28	4.37	4	69.59
3	A1AT	Alpha-1-antitrypsin	46.7	2100.8	36	44	7.29	20	95.11	0.67	14	53.67	3.05	16	55.02
4	A1BG	Alpha-1B-glycoprotein	54.2	1220.7	21	44.4	2.5	6	33.85	1.06	5	22.97	1.14	5	54.03
5	A2GL	Leucine-rich alpha-2-glycoprotein	38.2	742.6	13	39.5	1.22	4	10.19	0.88	4	15.78	1.68	5	45.68
6	AACT	Alpha-1-antichymotrypsin	47.6	480.4	10	29.6	0.85	1	N.D.	0.85	1	N.D.	1.13	5	37.41
7	ACTB	Actin, cytoplasmic 1	41.7	303	5	20.8	N.D.	N.D.	N.D.	N.D.	N.D.	N.D.	N.D.	N.D.	N.D.
8	ACTC	Actin, alpha cardiac muscle 1	42	230.2	5	20.7	0.65	1	N.D.	2.42	1	N.D.	N.D.	N.D.	N.D.
9	ACTG	Actin, cytoplasmic 2	41.8	594.3	11	41.9	0.36	2	64.42	0.88	2	132.9	N.D.	N.D.	N.D.
10	AFAM	Afamin	69	40.6	1	3.7	N.D.	N.D.	N.D.	N.D.	N.D.	N.D.	N.D.	N.D.	N.D.
11	ALBU	Serum albumin	69.3	4548.2	79	56.5	1.27	45	72.2	3.3	44	58.54	0.72	61	48.34
12	ALPK2	Alpha-protein kinase 2	236.9	35.6	1	0.4	0.13	1	N.D.	0.36	1	N.D.	1.08	1	N.D.
13	AMBP	Protein AMBP	39	1009.4	19	36.9	1.4	7	187.03	0.41	7	79.48	5.75	6	206.59
14	AMPD2	AMP deaminase 2	100.6	25.7	1	1.3	0.41	1		0.93	1	N.D.	N.D.	N.D.	N.D.
15	AMPN	Aminopeptidase N	109.5	772.4	16	20.1	0.71	2	45.13	1.05	2	22.07	N.D.	N.D.	N.D.
16	AMY1	Alpha-amylase 1	57.7	1443.2	24	40.9	0.35	9	92.92	0.99	10	29.83	0.52	11	80.31
17	AMYP	Pancreatic alpha-amylase	57.7	1687.4	27	46	0.27	9	71.93	1.03	11	37.92	0.49	12	86.7
18	AN30A	Ankyrin repeat domain-containing protein 30A	158.7	25.8	1	0.8	N.D.	N.D.	N.D.	N.D.	N.D.	N.D.	N.D.	N.D.	N.D.
19	AN34B	Ankyrin repeat domain-containing protein 34B	56.4	32.3	1	2.1	N.D.	N.D.	N.D.	N.D.	N.D.	N.D.	1.25	1	N.D.
20	ANAG	Alpha-N-acetylglucosaminidase	82.2	710.1	12	25	N.D.	N.D.	N.D.	N.D.	N.D.	N.D.	N.D.	N.D.	N.D.
21	ANO2	Anoctamin-2	113.9	25.1	1	1.1	N.D.	N.D.	N.D.	N.D.	N.D.	N.D.	0.8	1	N.D.

22	ANT3	Antithrombin-III	52.6	71	2	8	N.D.	N.D.	N.D.	N.D.	N.D.	N.D.	N.D.	N.D.	N.D.
23	ANXA1	Annexin A1	38.7	111.6	1	4.6	N.D.	N.D.	N.D.	N.D.	N.D.	N.D.	N.D.	N.D.	N.D.
24	ANXA2	Annexin A2	38.6	28	1	4.1	N.D.	N.D.	N.D.	N.D.	N.D.	N.D.	N.D.	N.D.	N.D.
25	ANXA5	Annexin A5	35.9	92.2	2	7.8	N.D.	N.D.	N.D.	N.D.	N.D.	N.D.	N.D.	N.D.	N.D.
26	APOA1	Apolipoprotein A-I	30.8	48.2	5	4.1	N.D.	N.D.	N.D.	N.D.	N.D.	N.D.	0.87	2	22.13
27	APOD	Apolipoprotein D	21.3	1330	27	39.2	0.26	15	70.9	0.21	16	84.85	1.51	4	61.23
28	APOH	Beta-2-glycoprotein 1	38.3	55.8	1	4.1	N.D.	N.D.	N.D.	N.D.	N.D.	N.D.	N.D.	N.D.	N.D.
29	APOL1	Apolipoprotein L1	43.9	80.3	2	5.8	N.D.	N.D.	N.D.	N.D.	N.D.	N.D.	N.D.	N.D.	N.D.
30	ARGAL	Rho guanine nucleotide exchange factor 10-like protein	140.3	25.7	1	0.5	N.D.	N.D.	N.D.	N.D.	N.D.	N.D.	N.D.	N.D.	N.D.
31	ARID2	AT-rich interactive domain-containing protein 2	197.3	32.6	1	0.4	N.D.	N.D.	N.D.	N.D.	N.D.	N.D.	0.68	1	N.D.
32	ARSA	Arylsulfatase A	53.6	304.6	6	15.8	0.79	1	N.D.	1.14	1	N.D.	N.D.	N.D.	N.D.
33	B2MG	Beta-2-microglobulin	13.7	178.1	4	19.3	1.5	4	90.85	0.95	4	41.36	1.42	4	17.18
34	B3GA3	Galactosylgalactosylxylosylprotein 3-beta-glucuronosyltransferase 3	37.1	25.5	1	3	N.D.	N.D.	N.D.	N.D.	N.D.	N.D.	N.D.	N.D.	N.D.
35	BGAL	Beta-galactosidase	76	222.4	5	9.6	N.D.	N.D.	N.D.	N.D.	N.D.	N.D.	N.D.	N.D.	N.D.
36	BLML	Putative beta-lactamase-like 1	54.6	27.4	1	1.6	N.D.	N.D.	N.D.	N.D.	N.D.	N.D.	N.D.	N.D.	N.D.
37	BT2A2	Butyrophilin subfamily 2 member A2	59	39.3	1	2.1	0.46	1	N.D.	0.6	1	N.D.	1.17	1	N.D.
38	BTD	Biotinidase	61.1	92.2	2	5.5	N.D.	N.D.	N.D.	N.D.	N.D.	N.D.	N.D.	N.D.	N.D.
39	C1RL	Complement C1r subcomponent-like protein	53.5	49.7	1	2.9	0.29	1	N.D.	0.48	1	N.D.	1.03	1	N.D.
40	CAD11	Cadherin-11	87.9	28	1	1.8	0.47	1	N.D.	1.18	1	N.D.	N.D.	N.D.	N.D.
41	CAD13	Cadherin-13	78.2	294.8	6	9	1.44	1	N.D.	0.58	1	N.D.	1.8	1	N.D.
42	CADH1	Cadherin-1	97.4	840.9	12	14.6	0.58	5	52.58	1.57	4	27.89	0.44	6	58.87
43	CADH2	Cadherin-2	99.7	108.2	2	3.3	N.D.	N.D.	N.D.	N.D.	N.D.	N.D.	N.D.	N.D.	N.D.
44	CADH6	Cadherin-6	88.3	53.1	1	1.6	N.D.	N.D.	N.D.	N.D.	N.D.	N.D.	N.D.	N.D.	N.D.
45	CADH8	Cadherin-8	88.2	30.3	1	1.3	N.D.	N.D.	N.D.	N.D.	N.D.	N.D.	N.D.	N.D.	N.D.

46	CADM4	Cell adhesion molecule 4	42.8	152.4	3	10.3	N.D.	N.D.	N.D.	N.D.	N.D.	N.D.	N.D.	N.D.	N.D.
47	CALM	Calmodulin	16.8	35.1	1	24.8	N.D.	N.D.	N.D.	N.D.	N.D.	N.D.	N.D.	N.D.	N.D.
48	CAN7	Calpain-7	92.6	26.5	1	1	3.15	1	N.D.	0.46	1	N.D.	N.D.	N.D.	N.D.
49	CATB	Cathepsin B	37.8	112.9	3	7.7	N.D.	N.D.	N.D.	N.D.	N.D.	N.D.	N.D.	N.D.	N.D.
50	CATC	Dipeptidyl peptidase 1	51.8	126.3	2	5.6	N.D.	N.D.	N.D.	N.D.	N.D.	N.D.	N.D.	N.D.	N.D.
51	CATD	Cathepsin D	44.5	146.9	3	7.3	N.D.	N.D.	N.D.	N.D.	N.D.	N.D.	1.38	2	4.84
52	CATZ	Cathepsin Z	33.8	42.4	1	4	N.D.	N.D.	N.D.	N.D.	N.D.	N.D.	N.D.	N.D.	N.D.
53	CBG	Corticosteroid-binding globulin	45.1	106.2	3	13.6	N.D.	N.D.	N.D.	N.D.	N.D.	N.D.	N.D.	N.D.	N.D.
54	CBPM	Carboxypeptidase M	50.5	65.8	2	7.2	N.D.	N.D.	N.D.	N.D.	N.D.	N.D.	N.D.	N.D.	N.D.
55	CBPQ	Carboxypeptidase Q	51.9	76.3	1	2.3	N.D.	N.D.	N.D.	N.D.	N.D.	N.D.	N.D.	N.D.	N.D.
56	CC171	Coiled-coil domain-containing protein 171	152.7	38.1	1	0.9	N.D.	N.D.	N.D.	N.D.	N.D.	N.D.	N.D.	N.D.	N.D.
57	CC28A	Coiled-coil domain-containing protein 28A	30.3	26.7	1	2.6	N.D.	N.D.	N.D.	N.D.	N.D.	N.D.	N.D.	N.D.	N.D.
58	CCD40	Coiled-coil domain-containing protein 40	130	25.3	1	0.6	2.5	1	N.D.	0.8	1	N.D.	N.D.	N.D.	N.D.
59	CD14	Monocyte differentiation antigen CD14	40.1	349.3	6	24.5	N.D.	N.D.	N.D.	1.07	1	N.D.	0.44	1	N.D.
60	CD248	Endosialin	80.8	210.5	5	6.5	N.D.	N.D.	N.D.	N.D.	N.D.	N.D.	N.D.	N.D.	N.D.
61	CD44	CD44 antigen	81.5	296.5	6	4.6	0.64	3	60.04	0.21	3	110.89	1.51	3	49.43
62	CD59	CD59 glycoprotein	14.2	118.4	3	15.6	0.66	2	39.46	0.35	2	74.17	1.57	2	34.01
63	CD7	T-cell antigen CD7	25.4	44.5	1	9.6	N.D.	N.D.	N.D.	N.D.	N.D.	N.D.	N.D.	N.D.	N.D.
64	CERU	Ceruloplasmin	122.1	693.2	12	15	1.87	4	28.26	1.27	4	25.3	1.72	5	29.68
65	CHMP5	Charged multivesicular body protein 5	24.6	369.2	4	12.3	N.D.	N.D.	N.D.	N.D.	N.D.	N.D.	N.D.	N.D.	N.D.
66	CILP1	Cartilage intermediate layer protein 1	132.5	33.1	1	1.3	N.D.	N.D.	N.D.	0.4	1	N.D.	0.82	1	N.D.
67	CLM9	CMRF35-like molecule 9	36	96.8	2	7.8	0.25	1	N.D.	0.58	1	N.D.	N.D.	N.D.	N.D.
68	CLUS	Clusterin	52.5	63	1	3.8	N.D.	N.D.	N.D.	N.D.	N.D.	N.D.	N.D.	N.D.	N.D.
69	CN105	Uncharacterized protein	34.7	25.1	1	2.4	0.49	1	N.D.	0.71	1	N.D.	N.D.	N.D.	N.D.

		C14orf105												
70	CNTFR	Ciliary neurotrophic factor receptor subunit alpha	40.6	51	1	3.5	N.D.	N.D.	N.D.	N.D.	N.D.	N.D.	N.D.	N.D.	N.D.
71	CNTRL	Centriolin	268.7	25.4	1	0.8	N.D.	N.D.	N.D.	N.D.	N.D.	N.D.	1.17	1	N.D.
72	CO4B	Complement C4-B	192.6	114	3	2.9	N.D.	N.D.	N.D.	N.D.	N.D.	N.D.	N.D.	N.D.	N.D.
73	CO6A1	Collagen alpha-1(VI) chain	108.5	584.3	11	11.2	N.D.	N.D.	N.D.	N.D.	N.D.	N.D.	N.D.	N.D.	N.D.
74	COFA1	Collagen alpha-1(XV) chain	141.6	64.1	1	1.1	N.D.	N.D.	N.D.	N.D.	N.D.	N.D.	N.D.	N.D.	N.D.
75	COIA1	Collagen alpha-1(XVIII) chain	178.1	58.5	2	2.5	N.D.	N.D.	N.D.	N.D.	N.D.	N.D.	N.D.	N.D.	N.D.
76	COL12	Collectin-12	81.5	95.6	2	3.6	0.42	1	N.D.	0.27	1	N.D.	N.D.	N.D.	N.D.
77	COMP	Cartilage oligomeric matrix protein	82.8	90.2	1	1.7	N.D.	N.D.	N.D.	N.D.	N.D.	N.D.	N.D.	N.D.	N.D.
78	CP089	UPF0764 protein C16orf89	45.4	32.7	1	3.2	N.D.	N.D.	N.D.	N.D.	N.D.	N.D.	N.D.	N.D.	N.D.
79	CPSM	Carbamoyl-phosphate synthase [ammonia], mitochondrial	164.8	26.6	1	0.5	1.31	1	N.D.	N.D.	N.D.	N.D.	N.D.	N.D.	N.D.
80	CSF1	Macrophage colony-stimulating factor 1	60.1	272	5	8.3	0.25	1	N.D.	0.22	1	N.D.	2.13	1	N.D.
81	CSN1	COP9 signalosome complex subunit 1	55.5	32.6	1	1	36.87	1	N.D.	4.25	1	N.D.	2.69	1	N.D.
82	CUBN	Cubilin	398.5	108.4	2	0.6	N.D.	N.D.	N.D.	N.D.	N.D.	N.D.	N.D.	N.D.	N.D.
83	CUTA	Protein CutA	19.1	137.9	2	22.9	N.D.	N.D.	N.D.	N.D.	N.D.	N.D.	N.D.	N.D.	N.D.
84	CUX2	Homeobox protein cut-like 2	161.6	27.7	1	0.9	N.D.	N.D.	N.D.	N.D.	N.D.	N.D.	N.D.	N.D.	N.D.
85	CYH1	Homeobox protein cut-like 2	46.3	29.8	1	1.3	N.D.	N.D.	N.D.	N.D.	N.D.	N.D.	1.36	1	N.D.
86	CYLC1	Cylicin-1	74.2	34.2	1	1.5	N.D.	N.D.	N.D.	N.D.	N.D.	N.D.	N.D.	N.D.	N.D.
87	CYTM	Cystatin-M	16.5	30.2	1	6.7	0.97	1	N.D.	0.31	1	N.D.	N.D.	N.D.	N.D.
88	DCP1B	mRNA-decapping enzyme 1B	67.7	29.4	1	1	N.D.	N.D.	N.D.	N.D.	N.D.	N.D.	3.72	1	N.D.
89	DERM	Dermatopontin	24	96.6	2	6.5	N.D.	N.D.	N.D.	N.D.	N.D.	N.D.	N.D.	N.D.	N.D.
90	DIAC	Di-N-acetylchitobiase	43.7	105.6	2	7.8	0.38	2	37.82	0.53	1	N.D.	0.67	2	0.28
91	DNAS1	Deoxyribonuclease-1	31.4	548.5	10	30.5	0.41	3	32.28	0.97	3	34.58	0.65	2	19.19
92	DPEP1	Dipeptidase 1	45.6	87.5	2	5.6	N.D.	N.D.	N.D.	N.D.	N.D.	N.D.	N.D.	N.D.	N.D.
93	DPP10	Inactive dipeptidyl peptidase 10	90.8	26	1	0.6	N.D.	N.D.	N.D.	N.D.	N.D.	N.D.	2.69	1	N.D.

94	DPP2	Dipeptidyl peptidase 2	54.3	26.5	1	4.1	N.D.	N.D.	N.D.	N.D.	N.D.	N.D.	N.D.	N.D.	N.D.
95	DPP4	Dipeptidyl peptidase 4	88.2	222.2	5	10.3	N.D.	N.D.	N.D.	N.D.	N.D.	N.D.	N.D.	N.D.	N.D.
96	DRC1	Dynein regulatory complex protein 1	87.1	28.4	1	0.9	0.13	1	N.D.	0.07	1	N.D.	N.D.	N.D.	N.D.
97	DSC2	Desmocollin-2	99.9	62.2	1	2.1	N.D.	N.D.	N.D.	N.D.	N.D.	N.D.	N.D.	N.D.	N.D.
98	DUS5	Dual specificity protein phosphatase 5	42	26.7	1	3.6	N.D.	N.D.	N.D.	N.D.	N.D.	N.D.	N.D.	N.D.	N.D.
99	DYH2	Dynein heavy chain 2, axonemal	507.4	25.7	1	0.2	N.D.	N.D.	N.D.	0.66	1	N.D.	N.D.	N.D.	N.D.
100	DYH5	Dynein heavy chain 5, axonemal	528.7	56.1	2	0.3	0.13	1	N.D.	0.89	1	N.D.	1.69	1	N.D.
101	EAA1	Excitatory amino acid transporter 1	59.5	30.7	1	1.3	1.16	1	N.D.	0.91	1	N.D.	N.D.	N.D.	N.D.
102	EGF	Pro-epidermal growth factor	133.9	42.6	1	1	N.D.	N.D.	N.D.	N.D.	N.D.	N.D.	N.D.	N.D.	N.D.
103	ENDD1	Endonuclease domain-containing 1 protein	55	314.7	5	16	N.D.	N.D.	N.D.	N.D.	N.D.	N.D.	N.D.	N.D.	N.D.
104	ENOA	Alpha-enolase	47.1	37.5	1	3.9	N.D.	N.D.	N.D.	N.D.	N.D.	N.D.	N.D.	N.D.	N.D.
105	EPCR	Endothelial protein C receptor	26.7	189.5	4	20.6	0.54	1	N.D.	0.94	1	N.D.	1.48	1	N.D.
106	EPHB4	Ephrin type-B receptor 4	108.2	28.7	1	1.1	N.D.	N.D.	N.D.	N.D.	N.D.	N.D.	N.D.	N.D.	N.D.
107	EZRI	Ezrin	69.4	93.4	3	4.8	N.D.	N.D.	N.D.	N.D.	N.D.	N.D.	N.D.	N.D.	N.D.
108	FA8	Coagulation factor VIII	266.8	30.9	1	0.4	N.D.	N.D.	N.D.	N.D.	N.D.	N.D.	1.32	1	N.D.
109	FAM3C	Protein FAM3C	24.7	35.3	1	7.5	N.D.	N.D.	N.D.	N.D.	N.D.	N.D.	N.D.	N.D.	N.D.
110	FBLN2	Fibulin-2	126.5	30.1	1	1.1	N.D.	N.D.	N.D.	N.D.	N.D.	N.D.	N.D.	N.D.	N.D.
111	FBLN3	EGF-containing fibulin-like extracellular matrix protein 1	54.6	99.5	2	7.9	N.D.	N.D.	N.D.	N.D.	N.D.	N.D.	N.D.	N.D.	N.D.
112	FCG3A	Low affinity immunoglobulin gamma Fc region receptor III-A	29.1	50.8	1	4.3	N.D.	N.D.	N.D.	N.D.	N.D.	N.D.	N.D.	N.D.	N.D.
113	FETUA	Alpha-2-HS-glycoprotein	39.3	644.8	13	34.1	0.57	4	57.87	1.06	4	44.37	0.43	2	23.26
114	FGF17	Fibroblast growth factor 17	24.9	30.1	1	4.6	N.D.	N.D.	N.D.	N.D.	N.D.	N.D.	1.05	1	N.D.
115	FIBA	Fibrinogen alpha chain	94.9	609.5	6	7.2	N.D.	N.D.	N.D.	N.D.	N.D.	N.D.	N.D.	N.D.	N.D.
116	FINC	Fibronectin	262.5	123.6	3	1.6	N.D.	N.D.	N.D.	N.D.	N.D.	N.D.	N.D.	N.D.	N.D.
117	FLNA	Filamin-A	280.6	27.2	1	0.5	N.D.	N.D.	N.D.	N.D.	N.D.	N.D.	N.D.	N.D.	N.D.

118	FRIL	Ferritin light chain	20	74.2	1	8.6	N.D.	N.D.	N.D.	N.D.	N.D.	N.D.	N.D.	N.D.	N.D.
119	GAS8	Growth arrest-specific protein 8	56.3	25.7	1	1.5	0.28	1	N.D.	0.37	1	N.D.	N.D.	N.D.	N.D.
120	GBP6	Guanylate-binding protein 6	72.4	25.6	1	1.3	N.D.	N.D.	N.D.	N.D.	N.D.	N.D.	N.D.	N.D.	N.D.
121	GELS	Gelsolin	85.6	1091.1	17	24.4	0.95	4	12.54	1.17	4	21.59	0.95	5	32.47
122	GGH	Gamma-glutamyl hydrolase	35.9	252.5	5	20.8	0.38	1	N.D.	0.41	1	N.D.	N.D.	N.D.	N.D.
123	GGTL2	Gamma-glutamyltransferase light chain 2	23.6	28.3	1	6	N.D.	N.D.	N.D.	N.D.	N.D.	N.D.	N.D.	N.D.	N.D.
124	GILT	Gamma-interferon-inducible lysosomal thiol reductase	27.9	61.6	2	4.4	N.D.	N.D.	N.D.	N.D.	N.D.	N.D.	N.D.	N.D.	N.D.
125	GNS	N-acetylglucosamine-6-sulfatase	62	73.9	2	3.8	N.D.	N.D.	N.D.	0.7	1	N.D.	N.D.	N.D.	N.D.
126	GOGA4	Golgin subfamily A member 4	261	28.8	1	0.6	N.D.	N.D.	N.D.	N.D.	N.D.	N.D.	0.48	1	N.D.
127	GP148	Probable G-protein coupled receptor 148	38.3	36.2	1	1.7	N.D.	N.D.	N.D.	N.D.	N.D.	N.D.	0.74	1	N.D.
128	GPC5B	G-protein coupled receptor family C group 5 member B	44.8	67.4	2	6.5	N.D.	N.D.	N.D.	N.D.	N.D.	N.D.	N.D.	N.D.	N.D.
129	GPC5C	G-protein coupled receptor family C group 5 member C	48.2	114.9	3	6.3	N.D.	N.D.	N.D.	N.D.	N.D.	N.D.	N.D.	N.D.	N.D.
130	GPX3	Glutathione peroxidase 3	25.6	44.8	1	5.3	N.D.	N.D.	N.D.	N.D.	N.D.	N.D.	N.D.	N.D.	N.D.
131	GSTA1	Glutathione S-transferase A1	25.6	46.4	1	12.2	N.D.	N.D.	N.D.	N.D.	N.D.	N.D.	N.D.	N.D.	N.D.
132	GSTM3	Glutathione S-transferase Mu 3	26.5	57.2	1	7.1	N.D.	N.D.	N.D.	N.D.	N.D.	N.D.	N.D.	N.D.	N.D.
133	GSTP1	Glutathione S-transferase P	23.3	33.1	1	7.6	N.D.	N.D.	N.D.	N.D.	N.D.	N.D.	N.D.	N.D.	N.D.
134	GVIN1	Interferon-induced very large GTPase 1	278.9	26.8	1	0.4	6.6	1	N.D.	0.5	1	N.D.	N.D.	N.D.	N.D.
135	H2A1H	Histone H2A type 1-H	13.9	42.2	1	22.7	N.D.	N.D.	N.D.	N.D.	N.D.	N.D.	N.D.	N.D.	N.D.
136	HAVR2	Hepatitis A virus cellular receptor 2	33.4	49.8	1	4	N.D.	N.D.	N.D.	N.D.	N.D.	N.D.	N.D.	N.D.	N.D.
137	HBA	Hemoglobin subunit alpha	15.2	52.5	1	11.3	N.D.	N.D.	N.D.	N.D.	N.D.	N.D.	1.83	1	N.D.
138	HBB	Hemoglobin subunit beta	16	30.3	1	6.8	N.D.	N.D.	N.D.	N.D.	N.D.	N.D.	1.54	1	N.D.
139	HEMO	Hemopexin	51.6	129.5	3	10	1.62	1	N.D.	1.08	1	N.D.	N.D.	N.D.	N.D.
140	HEXA	Beta-hexosaminidase subunit alpha	15.2	233	1	15.2	N.D.	N.D.	N.D.	N.D.	N.D.	N.D.	N.D.	N.D.	N.D.

141	HEXB	Beta-hexosaminidase subunit beta	16	134.2	1	11	N.D.	N.D.	N.D.	N.D.	N.D.	N.D.	N.D.	N.D.	N.D.
142	HMCN1	Hemicentin-1	613	215.9	3	0.5	N.D.	N.D.	N.D.	N.D.	N.D.	N.D.	N.D.	N.D.	N.D.
143	HPT	Haptoglobin	45.2	723.3	13	26.6	3.42	10	67.54	1.11	9	39.42	1.3	7	18.55
144	HSP71	Heat shock 70 kDa protein 1A/1B	70	140.3	3	4.5	N.D.	N.D.	N.D.	N.D.	N.D.	N.D.	N.D.	N.D.	N.D.
145	HV304	Ig heavy chain V-III region TIL	12.3	67.4	1	16.5	N.D.	N.D.	N.D.	N.D.	N.D.	N.D.	N.D.	N.D.	N.D.
146	HV316	Ig heavy chain V-III region TEI	12.8	89.5	1	16	N.D.	N.D.	N.D.	N.D.	N.D.	N.D.	N.D.	N.D.	N.D.
147	IC1	Plasma protease C1 inhibitor	55.1	115.3	3	9.8	N.D.	N.D.	N.D.	N.D.	N.D.	N.D.	N.D.	N.D.	N.D.
148	ICOSL	ICOS ligand	33.3	89.6	2	6.6	N.D.	N.D.	N.D.	N.D.	N.D.	N.D.	N.D.	N.D.	N.D.
149	IGHA1	Ig alpha-1 chain C region	37.6	598.5	12	27.5	1.68	4	47.53	2.16	3	48.94	0.79	5	29.4
150	IGHA2	Ig alpha-2 chain C region	36.5	403.6	8	25.9	1.55	3	61.14	2.21	3	44.7	0.79	3	33.56
151	IGHG1	Ig gamma-1 chain C region	36.1	306.6	5	14.8	0.16	2	32.99	0.59	2	45.66	0.67	3	47.43
152	IGJ	Immunoglobulin J chain	18.1	39.3	1	4.4	N.D.	N.D.	N.D.	N.D.	N.D.	N.D.	0.55	1	N.D.
153	IGKC	Ig kappa chain C region	11.6	2023.6	26	82.1	0.48	7	79.37	1.46	10	73.42	0.48	13	54.36
154	IGLL5	Immunoglobulin lambda-like polypeptide 5	23	853.3	14	31.8	0.9	9	106.59	1.34	9	89.39	N.D.	N.D.	N.D.
155	IL25	Interleukin-25	20.3	34.8	1	5.6	N.D.	N.D.	N.D.	N.D.	N.D.	N.D.	N.D.	N.D.	N.D.
156	IPSP	Plasma serine protease inhibitor	45.6	52.3	2	4.2	N.D.	N.D.	N.D.	N.D.	N.D.	N.D.	3.6	1	N.D.
157	IRF7	Interferon regulatory factor 7	54.2	37.1	1	1.8	N.D.	N.D.	N.D.	N.D.	N.D.	N.D.	N.D.	N.D.	N.D.
158	IST1	IST1 homolog	39.7	105.2	2	13.2	N.D.	N.D.	N.D.	N.D.	N.D.	N.D.	N.D.	N.D.	N.D.
159	ITIH4	Inter-alpha-trypsin inhibitor heavy chain H4	103.3	718.5	12	14.8	0.24	2	33.87	1.59	2	15.17	0.55	6	27.31
160	ITPR2	Inositol 1,4,5-trisphosphate receptor type 2	307.9	31.1	1	0.3	N.D.	N.D.	N.D.	N.D.	N.D.	N.D.	2.39	1	N.D.
161	K1C10	Keratin, type I cytoskeletal 10	58.8	690.2	10	16.8	0.3	2	3.84	0.63	1	N.D.	0.68	2	45.93
162	K1C13	Keratin, type I cytoskeletal 13	49.6	611.4	12	20.3	0.14	2	5.74	0.23	2	46.05	N.D.	N.D.	N.D.
163	K1C19	Keratin, type I cytoskeletal 19	44.1	87.3	2	4.5	N.D.	N.D.	N.D.	N.D.	N.D.	N.D.	N.D.	N.D.	N.D.
164	K1C9	Keratin, type I cytoskeletal 9	62	100.4	2	3.2	N.D.	N.D.	N.D.	N.D.	N.D.	N.D.	N.D.	N.D.	N.D.
165	K22E	Keratin, type II cytoskeletal 2	65.4	217.1	6	9.9	0.46	3	12.01	1.08	3	5.53	0.41	1	N.D.

		epidermal													
166	K2C1	Keratin, type II cytoskeletal 1	66	811.8	15	23	0.67	8	50.09	1.06	8	57.26	0.76	2	61.82
167	K2C4	Keratin, type II cytoskeletal 4	57.2	191.7	4	8.6	0.19	2	85.64	1.15	1	N.D.	N.D.	N.D.	N.D.
168	K2C6B	Keratin, type II cytoskeletal 6B	60	247.9	5	9.6	0.47	2	13.96	1.12	2	2.77	0.41	1	N.D.
169	KDM5A	Lysine-specific demethylase 5A	192	30.4	1	0.6	0.55	1	N.D.	2.68	1	N.D.	N.D.	N.D.	N.D.
170	KLK1	Kallikrein-1	28.9	322	5	17.9	0.28	1	N.D.	0.45	1	N.D.	0.86	2	23.8
171	KNG1	Kininogen-1	71.9	1705.2	28	14.1	0.43	11	45.71	0.56	11	63.61	0.55	7	40.93
172	KPRA	Phosphoribosyl pyrophosphate synthase-associated protein 1	39.4	26.7	1	4.5	0.68	1	N.D.	0.65	1	N.D.	N.D.	N.D.	N.D.
173	KV101	Ig kappa chain V-I region AG	12	406.2	4	31.5	0.35	1	N.D.	0.88	1	N.D.	0.62	1	N.D.
174	KV104	Ig kappa chain V-I region CAR	11.7	93.5	1	16.8	N.D.	N.D.	N.D.	N.D.	N.D.	N.D.	N.D.	N.D.	N.D.
175	KV105	Ig kappa chain V-I region DEE	11.7	156	2	16.7	N.D.	N.D.	N.D.	N.D.	N.D.	N.D.	N.D.	N.D.	N.D.
176	KV122	Ig kappa chain V-I region BAN	11.8	116.9	1	16.7	N.D.	N.D.	N.D.	N.D.	N.D.	N.D.	N.D.	N.D.	N.D.
177	KV203	Ig kappa chain V-II region MIL	12	31.5	1	17.9	0.92	1	N.D.	1.13	1	N.D.	1.88	1	N.D.
178	KV204	Ig kappa chain V-II region TEW	12.3	290.9	4	38.9	0.7	2	47.46	1.08	2	8.32	0.48	2	4.79
179	KV301	Ig kappa chain V-III region B6	11.6	33.2	1	16.7	0.46	1	N.D.	0.8	1	N.D.	0.69	1	N.D.
180	KV302	Ig kappa chain V-III region SIE	11.8	443.3	5	45.9	N.D.	N.D.	N.D.	N.D.	N.D.	N.D.	1	1	N.D.
181	KV305	Ig kappa chain V-III region WOL	11.7	440	5	45.9	0.71	1	N.D.	1.35	1	N.D.	N.D.	N.D.	N.D.
182	KV309	Ig kappa chain V-III region VG (Fragment)	12.6	67.6	2	7.8	0.22	1	N.D.	1.08	1	N.D.	0.55	1	N.D.
183	KV310	Ig kappa chain V-III region VH (Fragment)	12.7	45.4	1	15.5	N.D.	N.D.	N.D.	N.D.	N.D.	N.D.	N.D.	N.D.	N.D.
184	KV402	Ig kappa chain V-IV region Len	12.6	336.8	4	23.7	0.76	1	N.D.	1.34	1	N.D.	0.5	2	5.17
185	LAC1	Ig lambda-1 chain C regions	11.3	923.2	13	81.1	N.D.	N.D.	N.D.	N.D.	N.D.	N.D.	1	8	33.75
186	LAC2	Ig lambda-2 chain C regions	11.3	1254	19	81.1	N.D.	N.D.	N.D.	N.D.	N.D.	N.D.	1.33	13	28.73
187	LAC3	Ig lambda-3 chain C regions	11.2	988.8	15	74.5	0.84	8	95.63	0.99	8	77.18	N.D.	N.D.	N.D.
188	LAC7	Ig lambda-7 chain C region	11.3	568.7	9	50.9	1.38	3	81.66	1.96	3	59.41	1.29	6	57.65
189	LAIR1	Leukocyte-associated immunoglobulin-like receptor 1	31.4	60.6	2	8.4	N.D.	N.D.	N.D.	N.D.	N.D.	N.D.	N.D.	N.D.	N.D.

190	LAIR2	Leukocyte-associated immunoglobulin-like receptor 2	16.3	43.5	1	5.9	N.D.	N.D.	N.D.	N.D.	N.D.	N.D.	N.D.	N.D.	N.D.
191	LAMP1	Lysosome-associated membrane glycoprotein 1	44.9	25.2	1	3.8	N.D.	N.D.	N.D.	N.D.	N.D.	N.D.	N.D.	N.D.	N.D.
192	LCAT	Phosphatidylcholine-sterol acyltransferase	49.5	78.2	1	6.4	N.D.	N.D.	N.D.	N.D.	N.D.	N.D.	N.D.	N.D.	N.D.
193	LCOR	Ligand-dependent corepressor	47	30.5	1	2.3	N.D.	N.D.	N.D.	N.D.	N.D.	N.D.	0.65	1	N.D.
194	LENG8	Leukocyte receptor cluster member 8	86.1	31.5	1	1	N.D.	N.D.	N.D.	N.D.	N.D.	N.D.	N.D.	N.D.	N.D.
195	LG3BP	Galectin-3-binding protein	65.3	439.6	9	17.8	0.29	3	28.3	0.76	3	33.46	0.71	1	N.D.
196	LMAN2	Vesicular integral-membrane protein VIP36	40.2	659	11	28.7	0.37	2	97.65	0.74	3	15.12	0.86	2	10.69
197	LRP2	Low-density lipoprotein receptor-related protein 2	521.6	224.7	5	1.5	N.D.	N.D.	N.D.	N.D.	N.D.	N.D.	N.D.	N.D.	N.D.
198	LUM	Lumican	38.4	72.3	2	6.8	0.49	1	N.D.	0.41	1	N.D.	N.D.	N.D.	N.D.
199	LV301	Ig lambda chain V-III region SH	11.4	36.5	1	16.7	N.D.	N.D.	N.D.	N.D.	N.D.	N.D.	N.D.	N.D.	N.D.
200	LV302	Ig lambda chain V-III region LOI	11.9	29.5	1	7.2	N.D.	N.D.	N.D.	N.D.	N.D.	N.D.	N.D.	N.D.	N.D.
201	LV403	Ig lambda chain V-IV region Hil	11.5	38.7	1	17.8	N.D.	N.D.	N.D.	N.D.	N.D.	N.D.	N.D.	N.D.	N.D.
202	LYAG	Lysosomal alpha-glucosidase	105.3	1172.4	22	27.1	0.21	4	182.56	0.69	4	19.08	0.48	5	18.08
203	LYNX1	Ly-6/neurotoxin-like protein 1	14	25.9	1	9.9	0.35	1	N.D.	1.21	1	N.D.	0.29	1	N.D.
204	M3K5	Mitogen-activated protein kinase kinase kinase 5	154.4	48.7	1	0.7	N.D.	N.D.	N.D.	N.D.	N.D.	N.D.	2.64	1	N.D.
205	MA2B2	Epididymis-specific alpha-mannosidase	113.9	85.4	2	3.7	N.D.	N.D.	N.D.	N.D.	N.D.	N.D.	N.D.	N.D.	N.D.
206	MAGG1	Melanoma-associated antigen G1	34.3	26.7	1	3.3	N.D.	N.D.	N.D.	N.D.	N.D.	N.D.	N.D.	N.D.	N.D.
207	MAL	Myelin and lymphocyte protein	16.7	38.2	1	19	N.D.	N.D.	N.D.	N.D.	N.D.	N.D.	N.D.	N.D.	N.D.
208	MARK2	Mannan-binding lectin serine protease 2	87.9	25.1	1	1.3	N.D.	N.D.	N.D.	N.D.	N.D.	N.D.	1.35	1	N.D.
209	MASP2	Mannan-binding lectin serine protease 2	75.7	449.8	8	7.3	N.D.	N.D.	N.D.	N.D.	N.D.	N.D.	0.85	1	N.D.
210	MATN4	Matrilin-4	68.4	67.8	1	2.9	N.D.	N.D.	N.D.	N.D.	N.D.	N.D.	N.D.	N.D.	N.D.
211	MGA	Maltase-glucoamylase, intestinal	209.7	249	6	4	N.D.	N.D.	N.D.	N.D.	N.D.	N.D.	0.62	2	13.59

212	MMP20	Matrix metalloproteinase-20	54.4	25.2	1	1.9	0.1	1	N.D.	1.25	1	N.D.	1.29	1	N.D.
213	MOES	Moesin	67.8	91.4	3	4.9	N.D.	N.D.	N.D.	N.D.	N.D.	N.D.	N.D.	N.D.	N.D.
214	MOV10	Putative helicase MOV-10	113.6	30.6	1	0.8	N.D.	N.D.	N.D.	N.D.	N.D.	N.D.	N.D.	N.D.	N.D.
215	MPZL1	Myelin protein zero-like protein 1	29.1	25.4	1	2.2	N.D.	N.D.	N.D.	N.D.	N.D.	N.D.	12.19	1	N.D.
216	MRCKB	Serine/threonine-protein kinase MRCK beta	194.2	31.8	1	0.6	N.D.	N.D.	N.D.	N.D.	N.D.	N.D.	1.62	1	N.D.
217	MRP2	Canalicular multispecific organic anion transporter 1	174.1	27.8	1	0.7	0.19	1	N.D.	0.12	1	N.D.	N.D.	N.D.	N.D.
218	MSH6	DNA mismatch repair protein Msh6	152.7	27.2	1	0.7	N.D.	N.D.	N.D.	N.D.	N.D.	N.D.	1.41	1	N.D.
219	MUC1	Mucin-1	122	111.2	3	2.2	0.37	1	N.D.	0.72	1	N.D.	4.03	1	N.D.
220	MUCB	Ig mu heavy chain disease protein	43	35.1	1	2	N.D.	N.D.	N.D.	N.D.	N.D.	N.D.	N.D.	N.D.	N.D.
221	MYO15	Unconventional myosin-XV	395	29.2	1	0.2	N.D.	N.D.	N.D.	N.D.	N.D.	N.D.	N.D.	N.D.	N.D.
222	MYO5B	Unconventional myosin-Vb	213.5	68	2	0.4	2.67	2	33.76	0.93	2	31.39	1.11	2	3.85
223	NACC2	Nucleus accumbens-associated protein 2	62.8	27.3	1	1.7	N.D.	N.D.	N.D.	N.D.	N.D.	N.D.	N.D.	N.D.	N.D.
224	NB5R4	Cytochrome b5 reductase 4	59.4	28.6	1	1.5	0.26	1	N.D.	0.17	1	N.D.	N.D.	N.D.	N.D.
225	NEP	Neprilysin	85.5	82.1	2	2.9	N.D.	N.D.	N.D.	N.D.	N.D.	N.D.	N.D.	N.D.	N.D.
226	NF1	Neurofibromin	319.2	28.7	1	0.2	N.D.	N.D.	N.D.	N.D.	N.D.	N.D.	N.D.	N.D.	N.D.
227	NFH	Neurofilament heavy polypeptide	112.4	31.4	1	0.8	N.D.	N.D.	N.D.	N.D.	N.D.	N.D.	N.D.	N.D.	N.D.
228	NGN1	Neurogenin-1	25.7	35.9	1	3	N.D.	N.D.	N.D.	N.D.	N.D.	N.D.	N.D.	N.D.	N.D.
229	NID1	Nidogen-1	136.3	262.1	4	8.7	N.D.	N.D.	N.D.	N.D.	N.D.	N.D.	N.D.	N.D.	N.D.
230	NIPS1	Protein NipSnap homolog 1	33.3	26.3	1	2.5	N.D.	N.D.	N.D.	N.D.	N.D.	N.D.	N.D.	N.D.	N.D.
231	NOSIP	Nitric oxide synthase-interacting protein	33.2	29.8	1	3.7	N.D.	N.D.	N.D.	N.D.	N.D.	N.D.	N.D.	N.D.	N.D.
232	NPL	N-acetylneuraminate lyase	35.1	30.1	1	2.5	0.16	1	N.D.	0.15	1	N.D.	N.D.	N.D.	N.D.
233	NSD2	Histone-lysine N-methyltransferase NSD2	152.2	34.8	1	0.6	N.D.	N.D.	N.D.	N.D.	N.D.	N.D.	0.78	1	N.D.
234	NT5D2	5'-nucleotidase domain-containing protein 2	60.7	31.1	1	1.5	N.D.	N.D.	N.D.	N.D.	N.D.	N.D.	N.D.	N.D.	N.D.

235	NTF2	Nuclear transport factor 2	14.5	26.2	1	6.3	0.25	1	N.D.	0.33	1	N.D.	N.D.	N.D.	N.D.
236	NXPE4	NXPE family member 4	62.2	27.4	1	1.5	N.D.	N.D.	N.D.	N.D.	N.D.	N.D.	0.83	1	N.D.
237	OAS1	2'-5'-oligoadenylate synthase 1	46	25.4	1	2.8	1.06	1	N.D.	4.11	1	N.D.	1.09	1	N.D.
238	OLFM4	Olfactomedin-4	57.2	64.7	1	2.9	N.D.	N.D.	N.D.	N.D.	N.D.	N.D.	N.D.	N.D.	N.D.
239	OSCAR	Osteoclast-associated immunoglobulin-like receptor	30.5	176.4	4	17	0.45	1	N.D.	0.16	1	N.D.	N.D.	N.D.	N.D.
240	OSTP	Osteopontin	35.4	90.5	1	5.4	N.D.	N.D.	N.D.	N.D.	N.D.	N.D.	1.34	1	N.D.
241	PCP	Lysosomal Pro-X carboxypeptidase	55.8	55.7	1	4.2	N.D.	N.D.	N.D.	N.D.	N.D.	N.D.	N.D.	N.D.	N.D.
242	PDS5B	Sister chromatid cohesion protein PDS5 homolog B	164.6	26.8	1	0.8	N.D.	N.D.	N.D.	N.D.	N.D.	N.D.	1.85	1	N.D.
243	PEBP1	Phosphatidylethanolamine-binding protein 1	21	37.1	1	4.3	0.24	1	N.D.	0.3	1	N.D.	N.D.	N.D.	N.D.
244	PEPA3	Pepsin A-3	41.9	206.4	5	10.8	0.28	2	7.21	0.6	3	33.59	1.93	2	27.55
245	PGAM4	Probable phosphoglycerate mutase 4	28.8	34.9	1	7.1	N.D.	N.D.	N.D.	N.D.	N.D.	N.D.	N.D.	N.D.	N.D.
246	PGBM	Basement membrane-specific heparan sulfate proteoglycan core protein	468.5	1329.6	18	3.8	0.28	6	64.95	1.13	6	57.01	0.4	7	52.32
247	PGRP1	Peptidoglycan recognition protein 1	21.7	41	1	9.7	N.D.	N.D.	N.D.	N.D.	N.D.	N.D.	N.D.	N.D.	N.D.
248	PI16	Peptidase inhibitor 16	49.4	318	5	7.3	1.37	2	38	1.3	2	15.25	1.28	3	29.87
249	PIGR	Polymeric immunoglobulin receptor	83.2	150.4	4	10.3	N.D.	N.D.	N.D.	0.55	1	N.D.	0.81	1	N.D.
250	PKP2	Plakophilin-2	97.4	29.4	1	1.1	1.22	1	N.D.	0.49	1	N.D.	N.D.	N.D.	N.D.
251	PLSL	Plastin-2	70.2	31	1	2.4	N.D.	N.D.	N.D.	N.D.	N.D.	N.D.	N.D.	N.D.	N.D.
252	PPAP	Prostatic acid phosphatase	44.5	147.6	3	5.7	N.D.	N.D.	N.D.	N.D.	N.D.	N.D.	N.D.	N.D.	N.D.
253	PPIA	Peptidyl-prolyl cis-trans isomerase A	18	83.6	1	10.9	N.D.	N.D.	N.D.	N.D.	N.D.	N.D.	N.D.	N.D.	N.D.
254	PPR42	Protein phosphatase 1 regulatory subunit 42	40.9	31.3	1	2.8	N.D.	N.D.	N.D.	N.D.	N.D.	N.D.	N.D.	N.D.	N.D.
255	PRDX2	Peroxiredoxin-2	21.9	53.7	1	5.6	N.D.	N.D.	N.D.	N.D.	N.D.	N.D.	N.D.	N.D.	N.D.
256	PROF1	Profilin-1	15	48.2	1	10	N.D.	N.D.	N.D.	N.D.	N.D.	N.D.	N.D.	N.D.	N.D.

257	PROZ	Vitamin K-dependent protein Z	44.7	26.1	1	2.2	N.D.	N.D.	N.D.	N.D.	N.D.	N.D.	N.D.	N.D.	N.D.
258	PRUN2	Protein prune homolog 2	340.4	26.3	1	0.3	N.D.	N.D.	N.D.	N.D.	N.D.	N.D.	4.14	1	N.D.
259	PTGDS	Prostaglandin-H2 D-isomerase	21	435	6	17.4	1.4	2	17.39	0.75	2	34.49	1.31	2	74.01
260	PTN14	Tyrosine-protein phosphatase non-receptor type 14	135.2	30.2	1	1	8.09	1	N.D.	0.33	1	N.D.	N.D.	N.D.	N.D.
261	PTPRG	Receptor-type tyrosine-protein phosphatase gamma	161.9	72.9	1	1.9	N.D.	N.D.	N.D.	N.D.	N.D.	N.D.	N.D.	N.D.	N.D.
262	PTPRU	Receptor-type tyrosine-protein phosphatase U	162.3	28.3	1	0.3	N.D.	N.D.	N.D.	N.D.	N.D.	N.D.	0.54	1	N.D.
263	PVR	Poliovirus receptor	45.3	56.3	2	4.8	N.D.	N.D.	N.D.	N.D.	N.D.	N.D.	N.D.	N.D.	N.D.
264	PVRL2	Poliovirus receptor-related protein 2	57.7	39.6	1	1.5	N.D.	N.D.	N.D.	N.D.	N.D.	N.D.	N.D.	N.D.	N.D.
265	QIL1	Protein QIL1	13.1	28.2	1	5.9	N.D.	N.D.	N.D.	N.D.	N.D.	N.D.	5.11	1	N.D.
266	RAB34	Ras-related protein Rab-34	29	26.5	1	5	N.D.	N.D.	N.D.	N.D.	N.D.	N.D.	N.D.	N.D.	N.D.
267	RBM27	RNA-binding protein 27	118.6	30.5	1	1.3	1.81	1	N.D.	N.D.	N.D.	N.D.	N.D.	N.D.	N.D.
268	RBM34	RNA-binding protein 34	48.5	27.6	1	1.6	N.D.	N.D.	N.D.	N.D.	N.D.	N.D.	5.91	1	N.D.
269	REG1B	Lithostathine-1-beta	18.7	57.5	1	6.6	N.D.	N.D.	N.D.	N.D.	N.D.	N.D.	N.D.	N.D.	N.D.
270	RET4	Retinol-binding protein 4	23	232.5	6	27.4	0.88	4	26.15	0.69	4	11.14	0.96	2	15.44
271	RFC3	Replication factor C subunit 3	40.5	26.6	1	4.8	N.D.	N.D.	N.D.	N.D.	N.D.	N.D.	N.D.	N.D.	N.D.
272	RL40	Ubiquitin-60S ribosomal protein L40	14.7	89	2	14.1	N.D.	N.D.	N.D.	N.D.	N.D.	N.D.	N.D.	N.D.	N.D.
273	RN220	E3 ubiquitin-protein ligase RNF220	62.7	40.3	1	1.8	N.D.	N.D.	N.D.	N.D.	N.D.	N.D.	N.D.	N.D.	N.D.
274	RNAS2	Non-secretory ribonuclease	18.3	137.1	3	19.9	N.D.	N.D.	N.D.	N.D.	N.D.	N.D.	N.D.	N.D.	N.D.
275	ROBO4	Roundabout homolog 4	107.4	137.3	3	4.7	N.D.	N.D.	N.D.	N.D.	N.D.	N.D.	N.D.	N.D.	N.D.
276	RS16	40S ribosomal protein S16	16.4	26.6	1	5.5	N.D.	N.D.	N.D.	N.D.	N.D.	N.D.	1.38	1	N.D.
277	RYR3	Ryanodine receptor 3	551.7	26.8	1	0.2	N.D.	N.D.	N.D.	N.D.	N.D.	N.D.	N.D.	N.D.	N.D.
278	S10A6	Protein S100-A6	10.2	77.9	2	40	N.D.	N.D.	N.D.	N.D.	N.D.	N.D.	N.D.	N.D.	N.D.
279	S10A7	Protein S100-A7	11.5	137	2	19.8	N.D.	N.D.	N.D.	N.D.	N.D.	N.D.	N.D.	N.D.	N.D.

280	S10A8	Protein S100-A8	10.8	103.7	2	23.7	0.66	1	N.D.	0.44	1	N.D.	0.9	1	N.D.
281	S10A9	Protein S100-A9	13.2	545.1	9	74.6	0.12	3	46.76	0.58	4	109.97	0.72	3	13.47
282	S10AB	Protein S100-A11	11.7	36.7	1	15.2	1.15	1	N.D.		N.D.	N.D.	N.D.	N.D.	N.D.
283	S10AD	Protein S100-A13	11.5	26.6	1	10.2	N.D.	N.D.	N.D.	N.D.	N.D.	N.D.	1.65	1	N.D.
284	S10AG	Protein S100-A16	11.8	25.8	1	16.5	N.D.	N.D.	N.D.	N.D.	N.D.	N.D.	N.D.	N.D.	N.D.
285	S12A1	Solute carrier family 12 member 1	121.4	86.2	2	3.5	N.D.	N.D.	N.D.	N.D.	N.D.	N.D.	N.D.	N.D.	N.D.
286	S12A3	Solute carrier family 12 member 3	113.1	79.1	1	2.3	N.D.	N.D.	N.D.	N.D.	N.D.	N.D.	N.D.	N.D.	N.D.
287	S31D1	Spermatogenesis-associated protein 31D1	175.5	26.6	1	0.8	N.D.	N.D.	N.D.	N.D.	N.D.	N.D.	N.D.	N.D.	N.D.
288	SAP	Proactivator polypeptide	58.1	268.2	6	20.2	0.47	2	37.69	0.09	1	N.D.	N.D.	N.D.	N.D.
289	SAP3	Ganglioside GM2 activator	20.8	285.2	4	7.1	0.45	3	57.74	0.27	3	33.69	3.04	2	16.75
290	SCRN3	Secernin-3	48.5	26.6	1	1.9	N.D.	N.D.	N.D.	0.29	1	N.D.	N.D.	N.D.	N.D.
291	SCTM1	Secreted and transmembrane protein 1	27	285.6	4	14.1	0.39	1	N.D.	1.04	1	N.D.	0.98	2	48.28
292	SH3L3	SH3 domain-binding glutamic acid-rich-like protein 3	10.4	260.6	6	57	N.D.	N.D.	N.D.	N.D.	N.D.	N.D.	0.38	1	N.D.
293	SIK1	Serine/threonine-protein kinase SIK1	84.8	30	1	1.4	N.D.	N.D.	N.D.	N.D.	N.D.	N.D.	0.61	1	N.D.
294	SIRBL	Signal-regulatory protein beta-1 isoform 3	43.3	56.5	1	3.5	N.D.	N.D.	N.D.	N.D.	N.D.	N.D.	N.D.	N.D.	N.D.
295	SLAF5	SLAM family member 5	38.8	59.1	1	6.4	N.D.	N.D.	N.D.	N.D.	N.D.	N.D.	N.D.	N.D.	N.D.
296	SMC1A	Structural maintenance of chromosomes protein 1A	143.1	26.2	1	0.5	2.45	1	N.D.	0.49	1	N.D.	N.D.	N.D.	N.D.
297	SMCA2	Probable global transcription activator SNF2L2	181.2	33.1	1	0.6	0.35	1	N.D.	0.26	1	N.D.	1.81	1	N.D.
298	SMRC2	SWI/SNF complex subunit SMARCC2	132.8	25.1	1	1	N.D.	N.D.	N.D.	N.D.	N.D.	N.D.	1.11	1	N.D.
299	SODE	Extracellular superoxide dismutase [Cu-Zn]	25.8	38.4	1	4.6	N.D.	N.D.	N.D.	N.D.	N.D.	N.D.	N.D.	N.D.	N.D.
300	SPAG7	Sperm-associated antigen 7	26	25.4	1	4.4	N.D.	N.D.	N.D.	N.D.	N.D.	N.D.	0.81	1	N.D.
301	SPB3	Serpin B3	44.5	42.6	1	2.6	N.D.	N.D.	N.D.	N.D.	N.D.	N.D.	N.D.	N.D.	N.D.
302	SRC8	Src substrate cortactin	61.5	30.9	1	2.4	N.D.	N.D.	N.D.	N.D.	N.D.	N.D.	N.D.	N.D.	N.D.

303	SRRM4	Serine/arginine repetitive matrix protein 4	68.5	25.2	1	1.5	N.D.	N.D.	N.D.	0.4	1	N.D.	N.D.	N.D.	N.D.
304	SUCB1	Succinyl-CoA ligase [ADP-forming] subunit beta, mitochondrial	50.3	30.9	1	1.7	N.D.	N.D.	N.D.	N.D.	N.D.	N.D.	N.D.	N.D.	N.D.
305	SUCB2	Succinyl-CoA ligase [GDP-forming] subunit beta, mitochondrial	46.5	34	1	3.2	6.07	1	N.D.	0.41	1	N.D.	N.D.	N.D.	N.D.
306	SYCP1	Synaptonemal complex protein 1	114.1	30.1	1	1.4	N.D.	N.D.	N.D.	N.D.	N.D.	N.D.	N.D.	N.D.	N.D.
307	TAF4	Transcription initiation factor TFIID subunit 4	110	26.3	1	1.4	N.D.	N.D.	N.D.	N.D.	N.D.	N.D.	N.D.	N.D.	N.D.
308	TBD2B	TBC1 domain family member 2B	109.8	26	1	0.7	N.D.	N.D.	N.D.	N.D.	N.D.	N.D.	N.D.	N.D.	N.D.
309	TETN	Tetranectin	22.5	42.2	1	5.9	N.D.	N.D.	N.D.	N.D.	N.D.	N.D.	N.D.	N.D.	N.D.
310	THBG	Thyroxine-binding globulin	46.3	61.7	2	9.2	N.D.	N.D.	N.D.	N.D.	N.D.	N.D.	N.D.	N.D.	N.D.
311	THRB	Prothrombin	70	33.1	1	1.4	N.D.	N.D.	N.D.	N.D.	N.D.	N.D.	N.D.	N.D.	N.D.
312	THY1	Thy-1 membrane glycoprotein	17.9	56.4	1	9.3	N.D.	N.D.	N.D.	N.D.	N.D.	N.D.	N.D.	N.D.	N.D.
313	TI17A	Mitochondrial import inner membrane translocase subunit Tim17-A	18	26.8	1	5.3	N.D.	N.D.	N.D.	N.D.	N.D.	N.D.	N.D.	N.D.	N.D.
314	TMCC3	Transmembrane and coiled-coil domains protein 3	53.8	42.6	1	2.5	N.D.	N.D.	N.D.	N.D.	N.D.	N.D.	N.D.	N.D.	N.D.
315	TNIK	TRAF2 and NCK-interacting protein kinase	154.8	37.6	1	0.7	N.D.	N.D.	N.D.	N.D.	N.D.	N.D.	1.06	1	N.D.
316	TNK1	Non-receptor tyrosine-protein kinase TNK1	72.4	28.2	1	1.7	N.D.	N.D.	N.D.	N.D.	N.D.	N.D.	N.D.	N.D.	N.D.
317	TR19L	Tumor necrosis factor receptor superfamily member 19L	46.1	84.1	2	2.6	N.D.	N.D.	N.D.	N.D.	N.D.	N.D.	N.D.	N.D.	N.D.
318	TRBM	Thrombomodulin	60.3	75.3	1	3.5	N.D.	N.D.	N.D.	N.D.	N.D.	N.D.	N.D.	N.D.	N.D.
319	TRFE	Serotransferrin	77	3991.5	78	48.6	4.71	36	121.14	1.22	36	67.65	2.56	47	66.06
320	TRI36	E3 ubiquitin-protein ligase TRIM36	83	28.1	1	1.4	N.D.	N.D.	N.D.	N.D.	N.D.	N.D.	2.04	1	N.D.
321	TSN14	Tetraspanin-14	30.7	40.3	1	3	N.D.	N.D.	N.D.	N.D.	N.D.	N.D.	3.12	1	N.D.
322	TTC7A	Tetratricopeptide repeat protein 7A	96.1	25.1	1	1.2	N.D.	N.D.	N.D.	N.D.	N.D.	N.D.	0.43	1	N.D.

323	TTHY	Transthyretin	15.9	210	3	24.5	3.25	1	N.D.	2.11	1	N.D.	1.29	1	N.D.
324	TTLL7	Tubulin polyglutamylase TTLL7	102.9	71.9	2	1.4	0.21	1	N.D.	1.06	1	N.D.	0.48	1	N.D.
325	TXLNB	Beta-taxilin	76.5	28.4	1	1	N.D.	N.D.	N.D.	N.D.	N.D.	N.D.	0.42	1	N.D.
326	UFO	Tyrosine-protein kinase receptor UFO	98.3	143.2	3	4	N.D.	N.D.	N.D.	N.D.	N.D.	N.D.	N.D.	N.D.	N.D.
327	UROM	Uromodulin	69.7	2930.6	51	30.6	0.22	20	84.09	1.03	21	84.45	0.23	18	61.96
328	USP9Y	Probable ubiquitin carboxyl-terminal hydrolase FAF-Y	290.9	27.9	1	0.4	N.D.	N.D.	N.D.	N.D.	N.D.	N.D.	8.13	1	N.D.
329	UTER	Uteroglobin	10	739.1	11	41.8	1.45	4	49.84	0.93	4	34.91	1.29	4	14.74
330	VASN	Vasorin	71.7	353.3	5	15.9	N.D.	N.D.	N.D.	N.D.	N.D.	N.D.	N.D.	N.D.	N.D.
331	VCAM1	Vascular cell adhesion protein 1	81.2	189.7	4	4.7	0.61	2	62.04	0.84	2	25.1	0.76	3	32.82
332	VCIP1	Deubiquitinating protein VCIP135	134.2	27.2	1	0.6	N.D.	N.D.	N.D.	N.D.	N.D.	N.D.	0.95	1	N.D.
333	VMO1	Vitelline membrane outer layer protein 1 homolog	21.5	417.3	6	42.1	0.74	1	N.D.	1.12	1	N.D.	0.63	1	N.D.
334	VNN1	Pantetheinase	57	37.7	1	6.6	N.D.	N.D.	N.D.	N.D.	N.D.	N.D.	N.D.	N.D.	N.D.
335	VTDB	Vitamin D-binding protein	52.9	102.7	3	9.3	2.2	1	N.D.	1.16	1	N.D.	1.09	3	81.87
336	VTNC	Vitronectin	54.3	58.6	1	3.1	N.D.	N.D.	N.D.	N.D.	N.D.	N.D.	N.D.	N.D.	N.D.
337	WDR87	WD repeat-containing protein 87	333	30.8	1	0.3	2.17	1	N.D.	0.62	1	N.D.	N.D.	N.D.	N.D.
338	YJ017	Putative uncharacterized protein LOC439951	22.3	31.4	1	4.7	3	1	N.D.	1.04	1	N.D.	N.D.	N.D.	N.D.
339	YK042	Putative uncharacterized protein ENSP00000347057	10.2	25.5	0	16	N.D.	N.D.	N.D.	N.D.	N.D.	N.D.	N.D.	N.D.	N.D.
340	ZA2G	Zinc-alpha-2-glycoprotein	34.2	2013.4	36	56.7	0.75	22	83.98	1.01	22	38.2	1.03	22	32.88
341	ZN708	Zinc finger protein 708	64.7	27	1	1.4	N.D.	N.D.	N.D.	N.D.	N.D.	N.D.	2.46	1	N.D.

Table S1-2. Proteins identified by label-free quantitative proteomics.

							(WDM-NP:Healthy)			(DM-WNP:Healthy)			(DM-NP:DM-WNP Healthy)		
NO	Accession	Protein	MW (kDa)	Scores	Qualitative peptides	SC (%)	LF Median	Quantitative peptides	CV (%)	iTRAQ Median	Quantitative peptides	CV (%)	iTRAQ Median	Quantitative peptides	CV (%)
1	1433E	14-3-3 protein epsilon	29.2	96.7	2	11.4	N.D. (down)	N.D.	N.D.	0.37	1	N.D.	0.99	1	N.D.
2	1433Z	14-3-3 protein zeta/delta	27.7	133.7	2	9	N.D. (down)	N.D.	N.D.	0.37	1	N.D.	0.99	1	N.D.
3	A1AG1	Alpha-1-acid glycoprotein 1	23.5	7130.3	19	52.7	9.55	7	116.83	0.7	5	38	6.3	6	13.04
4	A1AG2	Alpha-1-acid glycoprotein 2	23.6	4666.1	13	51.7	8.73	7	69.92	0.74	6	44.73	5.26	7	28.24
5	A1AT	Alpha-1-antitrypsin	46.7	9142.6	37	65.1	10.49	16	42.81	0.54	13	63.87	5.18	14	42.85
6	A1BG	Alpha-1B-glycoprotein	54.2	3834.7	17	51.7	5.74	9	87.28	2.1	9	69.43	1.54	8	14.89
7	A2AP	Alpha-2-antiplasmin	54.5	29.2	1	2.6	N.D.	N.D.	N.D.	N.D.	N.D.	N.D.	N.D.	N.D.	N.D.
8	A2GL	Leucine-rich alpha-2-glycoprotein	38.2	3291.3	12	37.2	2.25	7	45.85	1.6	6	53.49	3.18	5	49.87
9	AACT	Alpha-1-antichymotrypsin	47.6	3135.7	16	38.5	2.84	8	44.77	0.84	7	28.75	3.11	9	24.6
10	ABCB7	ATP-binding cassette sub-family B member 7, mitochondrial	82.6	30.3	1	1.1	N.D.	N.D.	N.D.	0.66	1	N.D.	N.D.	N.D.	N.D.
11	ACBP	Acyl-CoA-binding protein	10	40.8	2	27.6	N.D.	N.D.	N.D.	0.46	1	N.D.	1.2	1	N.D.
12	ACTB	Actin, cytoplasmic 1	41.7	1388.3	13	38.4	0.29	5	44.36	0.65	5	39.4	1.31	5	48.05
13	ACTC	Actin, alpha cardiac muscle 1	42	757.9	8	19.9	0.3	2	51.89	0.51	4	22.87	1.32	4	54.1
14	ACTN4	Alpha-actinin-4	104.8	72.2	2	2.5	N.D.	N.D.	N.D.	1.07	1	N.D.	N.D.	N.D.	N.D.
15	AFAM	Afamin	69	1917	19	35.7	N.D. (up)	N.D.	N.D.	N.D. (up)	N.D.	N.D.	0.92	3	103.54
16	AGRIN	Agrin	217.1	102.1	3	1.5	N.D.	N.D.	N.D.	N.D.	N.D.	N.D.	N.D.	N.D.	N.D.
17	ALBU	Serum albumin	69.3	34933.4	71	78	2.77	38	164.85	4.38	34	127.2	0.62	52	50.69
18	ALDOB	Fructose-bisphosphate aldolase B	39.4	36.4	2	3.6	N.D.	N.D.	N.D.	N.D.	N.D.	N.D.	N.D.	N.D.	N.D.
19	AMBP	Protein AMBP	39	16156.4	27	52	2.2	20	138.75	0.54	15	88.25	3.2	19	317.4

20	AMPN	Aminopeptidase N	109.5	1270.4	21	29.3	0.19	4	49.14	0.51	10	45.23	1.13	10	22.66
21	AMY1	Alpha-amylase 1	57.7	5615.3	23	50.3	0.35	10	81.73	1.32	17	125.6	0.38	13	25.77
22	AMY2B	Alpha-amylase 2B	57.7	5373.5	23	49.9	0.35	10	81.73	1.39	16	127.65	0.39	12	25.72
23	AMYP	Pancreatic alpha-amylase	57.7	5796.9	25	61.3	0.3	11	83.27	1.34	17	124.78	0.38	13	26.82
24	ANAG	Alpha-N-acetylglucosaminidase	82.2	1347.4	14	26.1	0.25	3	39.66	0.97	10	26.56	0.71	7	16.94
25	ANGE2	Protein angel homolog 2	62.3	26	1	1.7	N.D.	N.D.	N.D.	N.D.	N.D.	N.D.	N.D.	N.D.	N.D.
26	ANGT	Angiotensinogen	53.1	289.8	4	8.9	2.79	2	17.93	N.D. (down)	N.D.	N.D.	N.D.	N.D.	N.D.
27	ANR26	Ankyrin repeat domain-containing protein 26	196.2	30.3	1	0.5	N.D.	N.D.	N.D.	N.D.	N.D.	N.D.	N.D.	N.D.	N.D.
28	ANT3	Antithrombin-III	52.6	849.3	12	30	2.77	3	45.65	N.D. (down)	N.D.	N.D.	N.D.(up)	N.D.	N.D.
29	ANX11	Annexin A11	54.4	42	1	2.2	N.D.	N.D.	N.D.	0.4	1	N.D.	1.06	1	N.D.
30	ANXA1	Annexin A1	38.7	927	9	36.4	N.D. (down)	N.D.	N.D.	0.78	6	25.67	0.83	6	9.13
31	ANXA2	Annexin A2	38.6	290.3	8	26.8	N.D. (down)	N.D.	N.D.	0.7	4	17.26	0.8	5	13.2
32	ANXA5	Annexin A5	35.9	73	1	5	N.D.	N.D.	N.D.	N.D.	N.D.	N.D.	N.D.	N.D.	N.D.
33	APOA	Apolipoprotein(a)	501	77.3	2	0.5	0.41	1	N.D.	N.D.	N.D.	N.D.	N.D.	N.D.	N.D.
34	APOA1	Apolipoprotein A-I	30.8	1183.9	10	40.8	1.67	5	21.24	1.78	5	132.16	1.03	3	109.84
35	APOA2	Apolipoprotein A-II	11.2	191.1	5	39	N.D.	N.D.	N.D.	N.D.	N.D.	N.D.	N.D.(up)	N.D.	N.D.
36	APOA4	Apolipoprotein A-IV	45.4	260.8	7	16.9	N.D. (up)	N.D.	N.D.	0.83	1	N.D.	N.D.	N.D.	N.D.
37	APOD	Apolipoprotein D	21.3	3823.4	10	34.9	0.54	8	116.19	0.45	7	157.4	1.79	8	30.16
38	APOH	Beta-2-glycoprotein 1	38.3	781	6	31	0.29	4	24.03	0.35	5	28.86	0.97	4	9.21
39	AQP2	Aquaporin-2	28.8	58.4	1	3.7	0.6	1	N.D.	2.04	1	N.D.	0.77	1	N.D.
40	ARSA	Arylsulfatase A	53.6	560.7	7	22.5	N.D.	N.D.	N.D.	1.38	3	28.18	0.65	2	44.21
41	ASAH1	Acid ceramidase	44.6	174.9	7	22.5	0.3	1		1.18	3	26.36	0.95	2	16.97
42	ASPG	N(4)-(beta-N-acetylglucosaminyl)-L-asparaginase	37.2	75.1	2	7.2	N.D.	N.D.	N.D.	N.D.	N.D.	N.D.	N.D.	N.D.	N.D.

43	ATRN	Attractin	158.4	258.3	6	4.8	1.02	3	21.89	N.D.	N.D.	N.D.	N.D.	N.D.	N.D.
44	B2MG	Beta-2-microglobulin	13.7	1058.5	9	60.5	2.24	2	7.2	0.36	1	N.D.	1.62	2	19.02
45	BGAL	Beta-galactosidase	76	662.1	14	30.6	N.D. (down)	N.D.	N.D.	1.09	5	22.66	0.37	6	25.1
46	BGLR	Beta-glucuronidase	74.7	50.8	1	4	N.D.	N.D.	N.D.	N.D.	N.D.	N.D.	N.D.	N.D.	N.D.
47	BRD3	Bromodomain-containing protein 3	79.5	29.2	1	2.1	N.D.	N.D.	N.D.	N.D.	N.D.	N.D.	N.D.	N.D.	N.D.
48	BT2A1	Butyrophilin subfamily 2 member A1	59.6	113.3	3	4.2	0.08	1	N.D.	N.D.(up)	N.D.	N.D.	N.D.	N.D.	N.D.
49	BTD	Biotinidase	61.1	912	6	16.4	1.01	4	22.23	0.9	4	21.93	0.97	3	0.81
50	C1RL	Complement C1r subcomponent-like protein	53.5	297.4	6	13.8	0.45	3	46.95	0.99	4	87.67	1.04	3	18.3
51	CAD11	Cadherin-11	87.9	281.9	8	11.1	0.46	1	N.D.	1.14	1		0.57	4	3.79
52	CAD13	Cadherin-13	78.2	1783.7	10	16.8	0.81	4	22.44	0.52	7	69.76	1.15	7	67.35
53	CADH1	Cadherin-1	97.4	3042.3	11	21.9	0.66	8	30.14	2.24	8	19.19	0.58	7	17.61
54	CADH2	Cadherin-2	99.7	406.5	9	13.9	0.46	2	0.01	1.13	6	14.18	0.64	4	17.15
55	CADH6	Cadherin-6	88.3	39.9	1	1.6	N.D.	N.D.	N.D.	N.D.	N.D.	N.D.	N.D.	N.D.	N.D.
56	CADM1	Cell adhesion molecule 1	48.5	116.2	1	4.3	N.D.	N.D.	N.D.	N.D.	N.D.	N.D.	N.D.	N.D.	N.D.
57	CADM3	Cell adhesion molecule 3	43.3	31.6	1	3	N.D.	N.D.	N.D.	N.D.	N.D.	N.D.	N.D.	N.D.	N.D.
58	CADM4	Cell adhesion molecule 4	42.8	464	6	22.4	0.5	2	7.91	0.75	3	11.9	0.64	3	40.97
59	CAH1	Carbonic anhydrase 1	28.9	104.2	4	18.8	N.D. (up)	N.D.	N.D.	N.D.	N.D.	N.D.	N.D. (up)	N.D.	N.D.
60	CALB1	Calbindin	30	87.8	3	12.3	N.D. (down)	N.D.	N.D.	0.34	1	N.D.	0.63	1	N.D.
61	CALM	Calmodulin	16.8	149.2	3	25.5	N.D. (down)	N.D.	N.D.	0.95	1	N.D.	0.32	1	N.D.
62	CATB	Cathepsin B	37.8	547.5	6	23.6	0.59	4	39.29	1.75	4	68.48	0.44	2	9.35
63	CATC	Dipeptidyl peptidase 1	51.8	769.8	7	25.9	0.73	4	44.77	1.22	5	29.42	0.82	5	12.91
64	CATD	Cathepsin D	44.5	639.5	8	23.5	0.82	2	40.85	1.21	6	52.6	1.16	5	6.92
65	CATH	Pro-cathepsin H	37.4	225.6	4	15.8	0.95	1	N.D.	3.91	1	N.D.	0.79	2	19.69

66	CATL1	Cathepsin L1	37.5	48.1	1	4.2	N.D.	N.D.	N.D.	N.D.	N.D.	N.D.	0.48	1	N.D.
67	CATZ	Cathepsin Z	33.8	346	3	12.5	0.69	2	6.23	0.73	2	37.59	1.49	1	N.D.
68	CBG	Corticosteroid-binding globulin	45.1	1035.2	8	23	3.24	4	60.74	0.71	2	22.19	3.12	2	20.09
69	CBPE	Carboxypeptidase E	53.1	25.7	1	4.6	N.D.	N.D.	N.D.	N.D.	N.D.	N.D.	N.D.	N.D.	N.D.
70	CBPM	Carboxypeptidase M	50.5	26.9	1	2.3	N.D.	N.D.	N.D.	N.D.	N.D.	N.D.	N.D.	N.D.	N.D.
71	CBPQ	Carboxypeptidase Q	51.9	417.4	6	15.3	3.18	1	N.D.	1.2	3	31.21	0.91	5	84.44
72	CBPZ	Carboxypeptidase Z	73.6	28.6	1	1.1	N.D.	N.D.	N.D.	N.D.	N.D.	N.D.	N.D.	N.D.	N.D.
73	CD14	Monocyte differentiation antigen CD14	40.1	1316.3	10	32.8	1.26	1	N.D.	1.55	9	55.06	0.58	9	20.31
74	CD248	Endosialin	80.8	775.5	10	19.9	0.33	2	70.99	0.85	5	99.38	0.47	4	5.93
75	CD27	CD27 antigen	29.1	96.5	2	8.8	2.85	1	N.D.	N.D.	N.D.	N.D.	N.D.	N.D.	N.D.
76	CD44	CD44 antigen	81.5	756.9	3	4.2	1.11	3	30.06	0.4	3	39.63	3.51	3	13.51
77	CD59	CD59 glycoprotein	14.2	1036.6	4	25	2.19	3	98.17	0.66	3	80.25	2.63	3	12.09
78	CD7	T-cell antigen CD7	25.4	51.6	1	9.2	N.D.	N.D.	N.D.	0.64	1	N.D.	N.D.	N.D.	N.D.
79	CDC42	Cell division control protein 42 homolog	21.2	26.3	1	5.8	N.D.	N.D.	N.D.	N.D.	N.D.	N.D.	N.D.	N.D.	N.D.
80	CDHR1	Cadherin-related family member 1	93.5	34.6	1	1.3	2.31	1	N.D.	1.75	1	N.D.	N.D.	N.D.	N.D.
81	CERU	Ceruloplasmin	122.1	7050.4	34	40.9	5.18	15	46.62	2.08	11	36.9	1.98	14	18.5
82	CFAB	Complement factor B	85.5	168.7	2	2.6	N.D.	N.D.	N.D.	N.D.	N.D.	N.D.	N.D.	N.D.	N.D.
83	CHM1B	Charged multivesicular body protein 1b	22.1	88.1	2	8.5	N.D.	N.D.	N.D.	N.D.	N.D.	N.D.	1.04	1	N.D.
84	CLIC1	Chloride intracellular channel protein 1	26.9	31.4	1	3.7	0.72	1	N.D.	0.17	1	N.D.	5.1	1	N.D.
85	CLM2	CMRF35-like molecule 2	22.9	50.5	1	5.9	N.D.	N.D.	N.D.	N.D.	N.D.	N.D.	N.D.	N.D.	N.D.
86	CLM8	CMRF35-like molecule 8	33.2	169.5	1	6	0.81	1	N.D.	0.6	1	N.D.	2.3	1	N.D.
87	CLM9	CMRF35-like molecule 9	36	457.1	3	12.7	1.25	2	59.01	1.98	2	16.61	1.04	3	12.02
88	CLMP	CXADR-like membrane protein	41.3	40.9	1	2.1	N.D.	N.D.	N.D.	N.D.	N.D.	N.D.	N.D.	N.D.	N.D.
89	CLUS	Clusterin	52.5	596	7	20.7	0.84	4	105.23	0.84	3	65.81	1	3	41.02

90	CNTFR	Ciliary neurotrophic factor receptor subunit alpha	40.6	133.9	1	3.5	N.D.	N.D.	N.D.	N.D.	N.D.	N.D.	N.D.	N.D.	N.D.
91	CO4B	Complement C4-B	192.6	561.4	5	4.1	0.31	1	N.D.	1.02	2	50.29	1.08	2	10.95
92	CO6A1	Collagen alpha-1(VI) chain	108.5	846.9	13	17	0.61	1	N.D.	0.68	8	48.75	0.81	7	12.73
93	CO6A3	Collagen alpha-3(VI) chain	343.5	71	2	0.5	N.D.	N.D.	N.D.	N.D.	N.D.	N.D.	N.D.	N.D.	N.D.
94	COBL	Protein cordon-bleu	135.5	40.2	1	0.7	N.D.	N.D.	N.D.	N.D.	N.D.	N.D.	1.32	1	N.D.
95	COCA1	Collagen alpha-1(XII) chain	332.9	243.4	1	0.4	N.D.	N.D.	N.D.	N.D.	N.D.	N.D.	0.57	1	N.D.
96	COFA1	Collagen alpha-1(XV) chain	141.6	107.5	2	1.6	N.D. (down)	N.D.	N.D.	N.D.	N.D.	N.D.	1.44	1	N.D.
97	COIA1	Collagen alpha-1(XVIII) chain	178.1	274.3	2	2.3	N.D.	N.D.	N.D.	N.D.	N.D.	N.D.	N.D.	N.D.	N.D.
98	COL12	Collectin-12	81.5	224.5	4	5.3	0.85	4	23.15	1.51	4	82.76	0.65	4	64.2
99	COMP	Cartilage oligomeric matrix protein	82.8	195.1	2	3.8	0.11	1	N.D.	0.2	1	N.D.	2.22	1	N.D.
100	CRIS3	Cysteine-rich secretory protein 3	27.6	32.1	1	3.7	N.D.	N.D.	N.D.	N.D.	N.D.	N.D.	N.D.	N.D.	N.D.
101	CRNN	Cornulin	53.5	538.8	5	18	N.D. (down)	N.D.	N.D.	0.41	1	N.D.	0.6	1	N.D.
102	CSF1	Macrophage colony-stimulating factor 1	60.1	471.5	4	7.9	0.21	3	22.73	0.23	2	7.4	1.65	1	N.D.
103	CSPG4	Chondroitin sulfate proteoglycan 4	250.4	65.5	1	0.4	N.D.	N.D.	N.D.	0.81	1	N.D.	0.77	1	N.D.
104	CTGF	Connective tissue growth factor	38.1	30.3	1	2.9	N.D.	N.D.	N.D.	N.D.	N.D.	N.D.	N.D.	N.D.	N.D.
105	CUBN	Cubilin	398.5	557.2	14	5.8	0.6	3	22.18	0.91	3	23.84	0.73	3	5.33
106	CUTA	Protein CutA	19.1	98.8	2	22.9	0.15	1	N.D.	N.D.	N.D.	N.D.	N.D.	N.D.	N.D.
107	CYLC1	Cylicin-1	74.2	32.2	1	1.5	N.D.	N.D.	N.D.	N.D.	N.D.	N.D.	N.D.	N.D.	N.D.
108	CYTA	Cystatin-A	11	396.4	4	53.1	2.68	3	46.15	N.D. (down)	N.D.	N.D.	N.D. (up)	N.D.	N.D.
109	CYTB	Cystatin-B	11.1	433.9	2	33.7	0.38	2	0	0.95	2	20.96	0.54	2	5.86
110	CYTC	Cystatin-C	15.8	133.4	3	18.5	N.D.	N.D.	N.D.	N.D.	N.D.	N.D.	N.D.	N.D.	N.D.
111	CYTM	Cystatin-M	16.5	42.5	5	10.3	1.91	3	56.87	0.31	1	N.D.	0.96	1	N.D.

112	CYTM1	Cysteine-rich and transmembrane domain-containing protein 1	10.6	385.2	1	50.3	N.D.	N.D.	N.D.	N.D.	N.D.	N.D.	N.D.	N.D.	N.D.
113	CYTS	Cystatin-S	16.2	81.3	2	17.7	N.D.	N.D.	N.D.	1.48	1	N.D.	0.67	1	N.D.
114	DAAM1	Disheveled-associated activator of morphogenesis 1	123.4	33.1	1	0.7	N.D.	N.D.	N.D.	N.D.	N.D.	N.D.	N.D.	N.D.	N.D.
115	DAF	Complement decay-accelerating factor	41.4	1001.5	9	28.3	1.19	3	11.77	N.D. (down)	N.D.	N.D.	N.D. (up)	N.D.	N.D.
116	DDAH2	N(G),N(G)-dimethylarginine dimethylaminohydrolase 2	29.6	32.4	1	2.8	N.D.	N.D.	N.D.	N.D.	N.D.	N.D.	N.D.	N.D.	N.D.
117	DEF1	Neutrophil defensin 1	10.2	294.8	2	19.1	0.8	2	2.95	9.65	2	0.12	0.37	2	8.53
118	DERM	Dermatopontin	24	499.1	4	21.9	0.46	3	38.52	0.83	3	50.5	1.11	2	6.66
119	DGKG	Diacylglycerol kinase gamma	89.1	35.3	1	1.3	0.13	1	N.D.	0.39	1	N.D.	0.63	1	N.D.
120	DIAC	Di-N-acetylchitobiase	43.7	1656.8	10	34.5	0.78	8	19.02	1.5	8	36.48	0.54	6	16.68
121	DLK1	Protein delta homolog 1	41.3	52	2	13.3	N.D.	N.D.	N.D.	N.D.	N.D.	N.D.	N.D.	N.D.	N.D.
122	DNAS1	Deoxyribonuclease-1	31.4	1229.1	8	43.6	0.12	2	0.84	0.63	6	61.63	0.52	3	7.43
123	DPP4	Dipeptidyl peptidase 4	88.2	762	15	20.2	0.69	6	42.39	1.15	7	49.53	1.15	11	33.75
124	DSC2	Desmocollin-2	99.9	551.1	9	17.3	0.51	4	88.8	0.8	5	46.28	0.75	4	26.47
125	EGF	Pro-epidermal growth factor	133.9	349.8	8	9.4	0.12	2	51.56	0.2	2	24.56	1.37	3	35.62
126	ENDD1	Endonuclease domain-containing 1 protein	55	398.3	8	18.2	0.44	6	46.24	0.86	6	37.85	0.9	3	15.05
127	ENOA	Alpha-enolase	47.1	30.8	1	2.1	N.D.	N.D.	N.D.	N.D.	N.D.	N.D.	N.D.	N.D.	N.D.
128	EPCR	Endothelial protein C receptor	26.7	822.2	3	16.4	0.13	3	38.41	0.29	3	76.69	0.61	2	5.77
129	EPHB2	Ephrin type-B receptor 2	117.4	35.7	1	1.1	N.D.	N.D.	N.D.	N.D.	N.D.	N.D.	N.D.	N.D.	N.D.
130	EZRI	Ezrin	69.4	41.4	1	1.4	N.D.	N.D.	N.D.	N.D.	N.D.	N.D.	N.D.	N.D.	N.D.
131	FAAA	Fumarylacetoacetase	46.3	34.7	1	2.9	0.06	1	N.D.	0.15	1	N.D.	0.79	1	N.D.
132	FABP5	Fatty acid-binding protein, epidermal	15.2	163.2	3	25.2	N.D. (down)	N.D.	N.D.	0.8	1	N.D.	N.D.	N.D.	N.D.
133	FAM3C	Protein FAM3C	24.7	148.9	3	18.1	0.6	1	N.D.	N.D.	N.D.	N.D.	N.D.	N.D.	N.D.

134	FBLN1	Fibulin-1	77.2	313.9	3	6.8	0.67	1	N.D.	N.D.	N.D.	N.D.	5.01	1	N.D.
135	FBLN2	Fibulin-2	126.5	1122.2	3	3.3	0.18	1	N.D.	0.33	1	N.D.	1.32	1	N.D.
136	FBLN3	EGF-containing fibulin-like extracellular matrix protein 1	54.6	1240.4	12	36.7	0.36	5	33.34	0.53	5	18.71	1.16	6	30.43
137	FBLN5	Fibulin-5	50.1	232.6	1	2.7	N.D.	N.D.	N.D.	N.D.	N.D.	N.D.	N.D.	N.D.	N.D.
138	FBN1	Fibrillin-1	312	508.2	5	2.5	0.44	3	46.41	0.63	1	N.D.	N.D. (up)	N.D.	N.D.
139	FBX38	F-box only protein 38	133.9	34.1	1	0.7	N.D.	N.D.	N.D.	N.D.	N.D.	N.D.	0.51	1	N.D.
140	FCG3A	Low affinity immunoglobulin gamma Fc region receptor III-A	29.1	254.5	3	10.6	0.1	1	N.D.	0.44	2	171.53	0.89	2	27.52
141	FETUA	Alpha-2-HS-glycoprotein	39.3	2918.8	8	26.2	2.17	6	156.39	1.93	7	214.08	0.87	5	37.78
142	FIBA	Fibrinogen alpha chain	94.9	2372.3	7	10.2	0.2	2	18.57	0.53	3	41.29	0.78	3	7.48
143	FINC	Fibronectin	262.5	1154.3	17	10.8	0.84	6	35.37	0.74	6	37.89	1.29	4	43.3
144	FLNC	Filamin-C	290.8	145.8	1	0.5	N.D.	N.D.	N.D.	N.D.	N.D.	N.D.	N.D.	N.D.	N.D.
145	FOLR1	Folate receptor alpha	29.8	125.4	3	12.8	0.24	2	11.73	N.D. (down)	N.D.	N.D.	4.21	1	N.D.
146	FRIH	Ferritin heavy chain	21.2	33.6	1	5.5	N.D.	N.D.	N.D.	N.D.	N.D.	N.D.	N.D.	N.D.	N.D.
147	FRIL	Ferritin light chain	20	40.7	1	5.1	N.D.	N.D.	N.D.	N.D.	N.D.	N.D.	N.D.	N.D.	N.D.
148	FZD2	Frizzled-2	63.5	49.6	1	1.4	N.D.	N.D.	N.D.	N.D.	N.D.	N.D.	N.D.	N.D.	N.D.
149	FZD4	Frizzled-4	59.8	149.1	2	4.3	0.45	2	24.38	N.D.	N.D.	N.D.	N.D.	N.D.	N.D.
150	GBB2	Guanine nucleotide-binding protein G(I)/G(S)/G(T) subunit beta-2	37.3	95.4	4	14.4	N.D. (down)	N.D.	N.D.	0.32	1	N.D.	1.31	1	N.D.
151	GBG12	Guanine nucleotide-binding protein G(I)/G(S)/G(O) subunit gamma-12	8	26.1	1	15.3	N.D.	N.D.	N.D.	N.D.	N.D.	N.D.	N.D.	N.D.	N.D.
152	GDF15	Growth/differentiation factor 15	34.1	53.9	1	5.2	N.D.	N.D.	N.D.	N.D.	N.D.	N.D.	0.95	1	N.D.
153	GDIR1	Rho GDP-dissociation inhibitor 1	23.2	51.9	1	3.4	N.D.	N.D.	N.D.	N.D.	N.D.	N.D.	N.D.	N.D.	N.D.
154	GELS	Gelsolin	85.6	4428.5	15	24.7	1.76	5	79.32	2.87	5	104.34	0.73	9	26.24

155	GGA2	ADP-ribosylation factor-binding protein	67.1	35.4	1	2	N.D.	N.D.	N.D.	N.D.	N.D.	N.D.	N.D.	N.D.	N.D.
156	GGH	Gamma-glutamyl hydrolase	35.9	681.4	6	24.2	1.47	3	744.82	1.27	4	40.18	0.46	3	32.51
157	GGT1	Gamma-glutamyltranspeptidase 1	61.4	41.1	2	3.7	N.D.	N.D.	N.D.	N.D.	N.D.	N.D.	N.D.	N.D.	N.D.
158	GILT	Gamma-interferon-inducible lysosomal thiol reductase	27.9	195.3	2	25.6	N.D.	N.D.	N.D.	N.D.	N.D.	N.D.	N.D.	N.D.	N.D.
159	GNAL	Guanine nucleotide-binding protein G(olf) subunit alpha	44.3	56.1	1	2.9	0.41	1	N.D.	0.25	1	N.D.	N.D.	N.D.	N.D.
160	GNS	N-acetylglucosamine-6-sulfatase	62	493.1	7	14.3	0.94	1	N.D.	1.15	3	15.64	0.86	4	19.73
161	GOLM1	Golgi membrane protein 1	45.3	32.6	2	5.2	0.71	1	N.D.	N.D.	N.D.	N.D.	N.D.	N.D.	N.D.
162	GPC5B	G-protein coupled receptor family C group 5 member B	44.8	71.8	1	3	N.D.	N.D.	N.D.	0.61	1	N.D.	N.D.	N.D.	N.D.
163	GPX3	Glutathione peroxidase 3	25.6	209.8	3	18.1	0.21	1	N.D.	1.04	2	9.35	0.67	2	5.83
164	GSTP1	Glutathione S-transferase P	23.3	120.1	1	9	N.D.	N.D.	N.D.	N.D.	N.D.	N.D.	N.D.	N.D.	N.D.
165	GUC2A	Guanylin	12.4	344.3	4	43.5	1.66	1	N.D.	N.D.	N.D.	N.D.	N.D.	N.D.	N.D.
166	HAVR2	Hepatitis A virus cellular receptor 2	33.4	248.4	4	13.6	0.32	3	13.11	0.35	4	70.07	1.14	3	6.17
167	HBA	Hemoglobin subunit alpha	15.2	190.8	3	42.3	0.37	1	N.D.	N.D.	N.D.	N.D.	N.D.	N.D.	N.D.
168	HBB	Hemoglobin subunit beta	16	705.8	10	82.3	0.49	2	81.98	0.6	1	N.D.	2.28	3	31
169	HEMO	Hemopexin	51.6	2907.6	19	51.5	2.08	12	28.91	0.99	12	63.44	3.08	11	21.52
170	HEXA	Beta-hexosaminidase subunit alpha	60.7	84.8	3	6	N.D. (down)	N.D.	N.D.	1.12	3	22.57	0.55	2	9.59
171	HEXB	Beta-hexosaminidase subunit beta	63.1	141.9	4	9.5	0.66	1	N.D.	1.64	3	15.64	0.49	1	N.D.
172	HGFA	Hepatocyte growth factor activator	70.6	162.7	3	4.7	N.D.	N.D.	N.D.	N.D.	N.D.	N.D.	0.71	1	N.D.
173	HMCN1	Hemicentin-1	613	389.1	5	1.4	0.36	2	32.08	0.73	3	76.08	0.79	2	11.77
174	HORN	Hornerin	282.2	157.6	1	0.6	N.D.	N.D.	N.D.	N.D.	N.D.	N.D.	N.D.	N.D.	N.D.
175	HPT	Haptoglobin	45.2	4025	26	53.4	9.56	12	87.79	1.79	13	65.41	1.48	13	27.33
176	HSP71	Heat shock 70 kDa protein 1A/1B	70	154	3	5.1	N.D.	N.D.	N.D.	0.42	1	N.D.	0.98	1	N.D.

177	HSP7C	Heat shock cognate 71 kDa protein	70.9	146.2	3	5.9	N.D.	N.D.	N.D.	0.42	1	N.D.	0.91	2	6.94
178	HSPB1	Heat shock protein beta-1	22.8	43.3	2	12.2	N.D.	N.D.	N.D.	2.16	1	N.D.	1.88	1	N.D.
179	HV101	Ig heavy chain V-I region EU	12.5	36.3	1	10.3	1.03	1	N.D.	1.98	1	N.D.	1.19	1	N.D.
180	HV304	Ig heavy chain V-III region TIL	12.3	448.7	2	26.1	1.06	1	N.D.	2.08	1	N.D.	0.78	2	4.91
181	HV305	Ig heavy chain V-III region BRO	13.2	762.8	2	25	1.05	1	N.D.	2.43	1	N.D.	0.91	2	17.78
182	HV307	Ig heavy chain V-III region CAM	13.7	193.3	3	18	0.49	1	N.D.	0.64	1	N.D.	1.18	2	8.07
183	I18BP	Interleukin-18-binding protein	21.1	67.8	1	4.1	N.D.	N.D.	N.D.	N.D.	N.D.	N.D.	3.13	1	N.D.
184	IC1	Plasma protease C1 inhibitor	55.1	657.9	6	12.2	0.35	4	92.27	0.4	4	72.75	1.67	4	8.12
185	ICA69	Islet cell autoantigen 1	54.6	25.2	1	2.3	N.D.	N.D.	N.D.	N.D.	N.D.	N.D.	N.D.	N.D.	N.D.
186	ICOSL	ICOS ligand	33.3	444.2	3	14.6	0.32	2	21.64	0.48	2	63.09	1.31	2	8.75
187	IDH3A	Isocitrate dehydrogenase [NAD] subunit alpha, mitochondrial	39.6	71	1	2.7	8.73	1	N.D.	N.D.	N.D.	N.D.	N.D.	N.D.	N.D.
188	IGHA1	Ig alpha-1 chain C region	37.6	3698.9	14	49.9	2.08	7	58.53	2.54	7	64.85	1.12	8	12.01
189	IGHA2	Ig alpha-2 chain C region	36.5	2950.2	11	50.3	1.42	5	51.79	2.1	5	24.04	0.97	6	35.23
190	IGHG1	Ig gamma-1 chain C region	36.1	1708.7	8	21.8	0.13	3	65.5	0.37	5	46.29	0.81	3	24.36
191	IGHG2	Ig gamma-2 chain C region	35.9	601.2	6	19.6	0.27	3	39.98	0.54	4	10.22	0.9	4	32.88
192	IGHG4	Ig gamma-4 chain C region	35.9	1319	8	22	0.17	3	102.71	0.39	5	51.02	1.07	3	15.74
193	IGHM	Ig mu chain C region	49.3	1060	5	10.2	0.54	2	5.09	1.26	3	35.47	1.11	4	20.03
194	IGJ	Immunoglobulin J chain	18.1	458.1	5	28.3	0.4	2	51.7	1.85	3	61.93	0.75	3	5.68
195	IGKC	Ig kappa chain C region	11.6	11224.7	12	82.1	0.98	7	101.23	4.63	7	166.77	0.46	9	35.74
196	IGLL1	Immunoglobulin lambda-like polypeptide 1	22.9	25.5	1	7	N.D.	N.D.	N.D.	N.D.	N.D.	N.D.	N.D.	N.D.	N.D.
197	IGLL5	Immunoglobulin lambda-like polypeptide 5	23	3224.7	6	40.7	1.88	2	7.9	2.18	1	N.D.	1.02	3	42.58
198	IGSF8	Immunoglobulin superfamily member 8	65	198.6	2	4.7	N.D.	N.D.	N.D.	3.27	1	N.D.	0.5	1	N.D.

199	INVO	Involucrin	68.4	101.1	2	3.9	N.D.	N.D.	N.D.	N.D.	N.D.	N.D.	N.D.	N.D.	N.D.
200	IPSP	Plasma serine protease inhibitor	45.6	33	1	2.7	N.D.	N.D.	N.D.	N.D.	N.D.	N.D.	N.D.	N.D.	N.D.
201	IST1	IST1 homolog	39.7	533.5	2	8	N.D.	N.D.	N.D.	N.D.	N.D.	N.D.	N.D.	N.D.	N.D.
202	ITIH4	Inter-alpha-trypsin inhibitor heavy chain H4	103.3	2900.2	11	14.9	N.D. (down)	N.D.	N.D.	1.24	9	61.93	0.61	6	15.43
203	K1C10	Keratin, type I cytoskeletal 10	58.8	5039.2	31	50.5	0.83	15	72.45	0.81	12	91.82	1.05	13	28.77
204	K1C13	Keratin, type I cytoskeletal 13	49.6	1129.4	19	31.4	0.15	5	134.18	0.44	11	90.9	0.35	1	N.D.
205	K1C14	Keratin, type I cytoskeletal 14	51.5	846.2	10	17.4	0.27	3	73.47	0.5	4	68.98	0.82	3	52.45
206	K1C9	Keratin, type I cytoskeletal 9	62	5105.4	27	55.5	1.78	8	78.03	2.37	6	38.28	0.67	9	50.18
207	K22E	Keratin, type II cytoskeletal 2 epidermal	65.4	2772.6	22	34.3	0.84	8	31.31	0.5	8	79.11	1.01	7	57.44
208	K2C1	Keratin, type II cytoskeletal 1	66	5717.9	29	41.1	0.83	14	49.78	0.88	13	66.66	0.64	17	35.73
209	K2C1B	Keratin, type II cytoskeletal 1b	61.9	651.1	5	8.3	1.39	2	4.09	1.67	2	16.5	0.61	3	29.18
210	K2C4	Keratin, type II cytoskeletal 4	57.2	660	9	15.2	1.09	1	N.D.	0.41	4	109.03	1.05	1	N.D.
211	K2C5	Keratin, type II cytoskeletal 5	62.3	1091.8	13	18.8	0.33	3	50.09	0.45	4	18.73	0.75	2	66.07
212	K2C6A	Keratin, type II cytoskeletal 6A	60	1198.8	14	22.2	0.3	5	77.86	0.31	5	62.51	1.03	5	73.41
213	KLK1	Kallikrein-1	28.9	1180.7	7	42	0.23	3	14.84	0.6	3	14.81	0.65	4	59.48
214	KLK3	Prostate-specific antigen	28.7	73.9	1	4.6	N.D.	N.D.	N.D.	N.D.	N.D.	N.D.	N.D.	N.D.	N.D.
215	KNG1	Kininogen-1	71.9	7303	27	34.6	0.29	14	21.11	0.64	16	35.56	0.66	15	23.64
216	KV101	Ig kappa chain V-I region AG	12	1762.1	3	31.5	0.57	2	2.71	2.06	2	22.1	0.64	3	20.81
217	KV102	Ig kappa chain V-I region AU	11.9	1362	3	31.5	0.64	2	13.34	1.93	2	28.89	0.63	3	23.86
218	KV105	Ig kappa chain V-I region DEE	11.7	1362.3	3	16.7	0.56	1	N.D.	2.56	1	N.D.	0.74	2	7.24
219	KV106	Ig kappa chain V-I region EU	11.8	506	2	26.9	0.72	1	N.D.	2.42	1	N.D.	1.09	1	N.D.
220	KV116	Ig kappa chain V-I region Roy	11.8	1354.4	3	38.9	0.56	1	N.D.	2.56	1	N.D.	0.74	2	7.24
221	KV117	Ig kappa chain V-I region Scw	11.8	1579.8	4	42.6	0.79	2	35.83	3.2	2	22.72	0.73	3	6.09

222	KV119	Ig kappa chain V-I region Wes	11.6	352.5	1	16.7	N.D.	N.D.	N.D.	3.14	1	N.D.	0.7	1	N.D.
223	KV120	Ig kappa chain V-I region Mev	11.9	151	1	16.5	N.D.	N.D.	N.D.	N.D.	N.D.	N.D.	1.02	1	N.D.
224	KV121	Ig kappa chain V-I region Ni	12.2	817.4	2	30.4	0.59	1	N.D.	1.92	2	15.01	0.64	2	27.87
225	KV122	Ig kappa chain V-I region BAN	11.8	354.2	1	16.7	N.D.	N.D.	N.D.	N.D.	N.D.	N.D.	N.D.	N.D.	N.D.
226	KV203	Ig kappa chain V-II region MIL	12	820.8	4	35.7	0.54	2	20.45	1.61	3	23.54	0.83	2	20.72
227	KV204	Ig kappa chain V-II region TEW	12.3	1288.2	3	35.4	0.48	3	24.03	1.67	3	19.25	0.95	3	25.6
228	KV206	Ig kappa chain V-II region RPMI 6410	14.7	787.3	3	18	0.54	2	20.45	1.84	2	17.16	0.83	2	20.72
229	KV301	Ig kappa chain V-III region B6	11.6	1117.4	2	31.5	0.42	1	N.D.	1.09	2	3.52	0.66	2	0.68
230	KV302	Ig kappa chain V-III region SIE	11.8	2114.5	3	39.4	0.43	2	2.31	1.78	3	39.78	0.63	3	11.74
231	KV309	Ig kappa chain V-III region VG (Fragment)	12.6	239.5	1	7.8	0.45	1	N.D.	2.27	1	N.D.	0.87	1	N.D.
232	KV310	Ig kappa chain V-III region VH (Fragment)	12.7	78.8	2	23.3	0.54	2	10.85	2.37	2	4.39	0.88	2	1.66
233	KV311	Ig kappa chain V-III region IARC/BL41	14.1	102.7	1	12.5	N.D.	N.D.	N.D.	N.D.	N.D.	N.D.	N.D.	N.D.	N.D.
234	KV401	Ig kappa chain V-IV region (Fragment)	13.4	375	3	29.8	0.41	2	21.16	1.02	2	1.09	1.01	2	8.07
235	KV402	Ig kappa chain V-IV region Len	12.6	1054.5	3	36.8	0.44	3	20.31	1.34	3	40.33	0.81	3	32.77
236	LAC2	Ig lambda-2 chain C regions	11.3	3364.5	5	74.5	1.88	2	7.9	2.18	1	N.D.	0.9	2	47.04
237	LAC7	Ig lambda-7 chain C region	11.3	1808.3	3	41.5	1.74	1	N.D.	2.18	1	N.D.	1.4	1	N.D.
238	LAIR1	Leukocyte-associated immunoglobulin-like receptor 1	31.4	326.8	2	9.1	2.42	1	N.D.	N.D.	N.D.	N.D.	N.D. (up)	N.D.	N.D.
239	LAMP1	Lysosome-associated membrane glycoprotein 1	44.9	94.7	1	2.2	N.D.	N.D.	N.D.	N.D.	N.D.	N.D.	N.D.	N.D.	N.D.
240	LAMP2	Lysosome-associated membrane glycoprotein 2	44.9	59.9	2	3.9	0.68	1	N.D.	N.D.	N.D.	N.D.	N.D.	N.D.	N.D.

241	LCN1	Lipocalin-1	19.2	133.5	3	12.5	1.69	1	N.D.	N.D.	N.D.	N.D.	1.75	1	N.D.
242	LEG9B	Galectin-9B	39.6	71	1	3.4	N.D.	N.D.	N.D.	2.41	1	N.D.	0.56	1	N.D.
243	LG3BP	Galectin-3-binding protein	65.3	2232.5	12	27.9	0.28	7	46.31	0.86	10	28.2	0.78	9	15.08
244	LMAN2	Vesicular integral-membrane protein VIP36	40.2	3150.1	14	39.9	0.15	10	20.5	0.67	12	45.83	1.16	11	17.36
245	LRP2	Low-density lipoprotein receptor-related protein 2	521.6	1360.1	15	3.5	0.42	6	55.39	0.95	8	48.08	0.75	7	50.5
246	LTBP2	Latent-transforming growth factor beta-binding protein 2	194.9	267.4	1	1	N.D.	N.D.	N.D.	N.D.	N.D.	N.D.	N.D.	N.D.	N.D.
247	LUM	Lumican	38.4	146.2	4	13.3	N.D.	N.D.	N.D.	1.29	1	N.D.	1.25	1	N.D.
248	LV001	Ig lambda chain V region 4A	12.4	91.5	2	13.7	N.D.	N.D.	N.D.	N.D.	N.D.	N.D.	2.23	1	N.D.
249	LV102	Ig lambda chain V-I region HA	11.9	69.2	2	18.8	N.D.	N.D.	N.D.	N.D.	N.D.	N.D.	N.D.	N.D.	N.D.
250	LV103	Ig lambda chain V-I region NEW	11.4	145.9	1	7.2	N.D.	N.D.	N.D.	N.D.	N.D.	N.D.	1.86	1	N.D.
251	LV301	Ig lambda chain V-III region SH	11.4	468.2	3	34.3	N.D.	N.D.	N.D.	N.D.	N.D.	N.D.	1.02	1	N.D.
252	LV302	Ig lambda chain V-III region LOI	11.9	369.8	2	21.6	3.16	1	N.D.	6.09	1	N.D.	1.71	2	4.84
253	LV401	Ig lambda chain V-IV region Bau	11.3	103.4	1	7.5	N.D.	N.D.	N.D.	N.D.	N.D.	N.D.	1.83	1	N.D.
254	LV403	Ig lambda chain V-IV region Hil	11.5	125.3	2	28	0.61	1	N.D.	0.7	1	N.D.	1.41	1	N.D.
255	LYAG	Lysosomal alpha-glucosidase	105.3	2931.4	20	31.4	0.31	8	46.65	1	16	70.69	0.46	10	36.74
256	LYNX1	Ly-6/neurotoxin-like protein 1	14	139	1	23.7	N.D.	N.D.	N.D.	N.D.	N.D.	N.D.	N.D.	N.D.	N.D.
257	LYVE1	Lymphatic vessel endothelial hyaluronic acid receptor 1	35.2	606.3	5	14.3	4.58	3	113.87	0.66	2	26.72	2.41	4	29.59
258	M3K9	Mitogen-activated protein kinase kinase kinase 9	121.8	45.1	1	1.1	0.79	1	N.D.	N.D.	N.D.	N.D.	N.D.	N.D.	N.D.
259	MAP2	Microtubule-associated protein 2	199.4	32.1	1	0.7	N.D.	N.D.	N.D.	N.D.	N.D.	N.D.	N.D.	N.D.	N.D.
260	MASP2	Mannan-binding lectin serine protease 2	75.7	2054.7	7	12.2	0.45	5	59.85	1.83	6	111.2	0.57	5	75.08

261	MATN4	Matrilin-4	68.4	316.9	1	2.9	N.D.	N.D.	N.D.	N.D.	N.D.	N.D.	N.D.	N.D.	N.D.
262	MCR	Mineralocorticoid receptor	107	33.7	1	1.1	0.66	1	N.D.	1.35	1	N.D.	N.D.	N.D.	N.D.
263	MGA	Maltase-glucoamylase, intestinal	209.7	518.2	14	9.7	0.39	5	51.3	1.13	6	49.64	0.49	8	28.2
264	MIEN1	Migration and invasion enhancer 1	12.4	348.6	3	38.3	0.28	1	N.D.	0.96	2	3.79	0.77	2	18.33
265	MMP20	Matrix metalloproteinase-20	54.4	34.8	1	1.9	0.1	1	N.D.	0.76	1	N.D.	2.03	1	N.D.
266	MRRP1	Mitochondrial ribonuclease P protein 1	47.3	28.9	1	1.7	N.D.	N.D.	N.D.	1.4	1	N.D.	2.66	1	N.D.
267	MSMB	Beta-microseminoprotein	12.9	111.8	3	44.7	0.35	1	N.D.	N.D.	N.D.	N.D.	N.D.	N.D.	N.D.
268	MUC1	Mucin-1	122	289.3	4	3.8	N.D.	N.D.	N.D.	0.56	2	52.87	1.28	2	13.07
269	MXRA8	Matrix-remodeling-associated protein 8	49.1	83.7	3	6.3	N.D. (down)	N.D.	N.D.	N.D. (down)	N.D.	N.D.	N.D.	N.D.	N.D.
270	NAGAB	Alpha-N-acetylgalactosaminidase	46.5	31.3	1	3.4	N.D.	N.D.	N.D.	N.D.	N.D.	N.D.	N.D.	N.D.	N.D.
271	NAPSA	Napsin-A	45.4	122	2	8.1	N.D.	N.D.	N.D.	N.D.	N.D.	N.D.	N.D.	N.D.	N.D.
272	NBL1	Neuroblastoma suppressor of tumorigenicity 1	19.3	36.8	1	6.1	N.D.	N.D.	N.D.	N.D.	N.D.	N.D.	N.D.	N.D.	N.D.
273	NCAM1	Neural cell adhesion molecule 1	94.5	80.8	1	1.3	N.D.	N.D.	N.D.	N.D.	N.D.	N.D.	N.D.	N.D.	N.D.
274	NCF1B	Putative neutrophil cytosol factor 1B	44.8	29	1	2	N.D.	N.D.	N.D.	N.D.	N.D.	N.D.	N.D.	N.D.	N.D.
275	NEGR1	Neuronal growth regulator 1	38.7	307.2	2	9.3	0.44	2	0.6	0.5	2	17.39	1.52	2	18.62
276	NEUS	Neuroserpin	46.4	30.8	1	2.2	N.D.	N.D.	N.D.	N.D.	N.D.	N.D.	N.D.	N.D.	N.D.
277	NHLC3	NHL repeat-containing protein 3	38.3	66.3	1	3.5	N.D.	N.D.	N.D.	N.D.	N.D.	N.D.	N.D.	N.D.	N.D.
278	NID1	Nidogen-1	136.3	991.2	7	11.4	0.18	1	N.D.	0.75	2	12.01	0.79	3	13.52
279	NOL7	Nucleolar protein 7	29.4	50.2	2	7.4	N.D.	N.D.	N.D.	N.D.	N.D.	N.D.	N.D.	N.D.	N.D.
280	NOV	Protein NOV homolog	39.1	104.7	2	7	0.97	1	N.D.	N.D.	N.D.	N.D.	N.D.	N.D.	N.D.
281	NPC2	Epididymal secretory protein E1	16.6	62.4	2	17.2	2.37	1	N.D.	N.D.	N.D.	N.D.	N.D.	N.D.	N.D.
282	NSF1C	NSFL1 cofactor p47	40.5	73.4	1	3.2	N.D.	N.D.	N.D.	0.99	1	N.D.	0.86	1	N.D.

283	NTF2	Nuclear transport factor 2	14.5	332.8	5	69.3	0.16	2	13.54	0.51	3	9.04	1.29	3	8.7
284	OLFM4	Olfactomedin-4	57.2	165.3	3	8.4	N.D.	N.D.	N.D.	N.D.	N.D.	N.D.	N.D.	N.D.	N.D.
285	OSCAR	Osteoclast-associated immunoglobulin-like receptor	30.5	61.4	1	3.5	N.D.	N.D.	N.D.	N.D.	N.D.	N.D.	N.D.	N.D.	N.D.
286	OSTP	Osteopontin	35.4	1571.5	12	46.2	4.83	3	40.27	N.D. (down)	N.D.	N.D.	N.D. (up)	N.D.	N.D.
287	P3IP1	Phosphoinositide-3-kinase-interacting protein 1	28.2	616.9	5	20.9	1.17	2	53.24	N.D. (down)	N.D.	N.D.	3.81	2	10.38
288	PAPP2	Pappalysin-2	198.4	27.7	1	0.5	N.D.	N.D.	N.D.	N.D.	N.D.	N.D.	N.D.	N.D.	N.D.
289	PCDGK	Protocadherin gamma-C3	101	30.1	1	1.1	N.D.	N.D.	N.D.	N.D.	N.D.	N.D.	N.D.	N.D.	N.D.
290	PCDH1	Protocadherin-1	114.7	58.9	2	3.8	N.D.	N.D.	N.D.	N.D.	N.D.	N.D.	N.D.	N.D.	N.D.
291	PCNT	Pericentrin	377.8	31.1	1	0.2	3.8	1	N.D.	N.D.	N.D.	N.D.	N.D.	N.D.	N.D.
292	PCP	Lysosomal Pro-X carboxypeptidase	55.8	135.3	2	6.5	N.D.	N.D.	N.D.	N.D.	N.D.	N.D.	N.D.	N.D.	N.D.
293	PEBP1	Phosphatidylethanolamine-binding protein 1	21	348.2	4	32.1	N.D. (down)	N.D.	N.D.	0.72	2	0.53	N.D.	N.D.	N.D.
294	PEPA3	Pepsin A-3	41.9	169.7	4	6.7	0.03	1	N.D.	0.37	3	42.18	2.19	2	13.05
295	PGBM	Basement membrane-specific heparan sulfate proteoglycan core protein	468.5	4377.4	16	4.6	0.25	8	48.93	1.29	9	46.98	0.53	10	21.74
296	PGRP1	Peptidoglycan recognition protein 1	21.7	66.2	1	11.7	N.D.	N.D.	N.D.	N.D.	N.D.	N.D.	N.D.	N.D.	N.D.
297	PGRP2	N-acetylmuramoyl-L-alanine amidase	62.2	132.4	3	8.9	0.51	1	N.D.	1.09	2	5.75	N.D.	N.D.	N.D.
298	PI16	Peptidase inhibitor 16	49.4	2738.5	9	27.6	1.53	4	16.95	1.23	4	16.68	1.34	6	28.8
299	PIGR	Polymeric immunoglobulin receptor	83.2	2324.8	21	34.2	0.39	6	44.25	1.73	9	88.17	0.54	12	40.67
300	PIP	Prolactin-inducible protein	16.6	128.3	3	26	0.27	2	18.34	0.5	1	N.D.	N.D.	N.D.	N.D.
301	PLBL2	Putative phospholipase B-like 2	65.4	37.4	1	2	N.D.	N.D.	N.D.	N.D.	N.D.	N.D.	N.D.	N.D.	N.D.
302	PLMN	Plasminogen	90.5	54.9	1	2.3	N.D.	N.D.	N.D.	N.D.	N.D.	N.D.	N.D.	N.D.	N.D.
303	PLOD1	Procollagen-lysine, 2-oxoglutarate 5-dioxygenase 1	83.5	26.1	1	1.2	3.4	1	N.D.	0.37	1	N.D.	3.42	1	N.D.

304	PLSL	Plastin-2	70.2	41.1	1	2.4	N.D.	N.D.	N.D.	N.D.	N.D.	N.D.	N.D.	N.D.	N.D.
305	PPAL	Lysosomal acid phosphatase	48.3	209.9	5	10.6	N.D. (down)	N.D.	N.D.	1.17	3	15.05	0.5	3	15.33
306	PPAP	Prostatic acid phosphatase	44.5	478.6	9	31.9	N.D. (down)	N.D.	N.D.	0.35	4	75.36	2.33	5	13.44
307	PPGB	Lysosomal protective protein	54.4	266.2	3	6.9	N.D.	N.D.	N.D.	1.09	1	N.D.	0.55	2	8.78
308	PPIA	Peptidyl-prolyl cis-trans isomerase A	18	58.1	1	10.9	N.D.	N.D.	N.D.	N.D.	N.D.	N.D.	N.D.	N.D.	N.D.
309	PRD11	PR domain-containing protein 11	57.8	31.5	1	1.6	N.D.	N.D.	N.D.	N.D.	N.D.	N.D.	N.D.	N.D.	N.D.
310	PRIO	Major prion protein	27.6	297.4	1	4.7	0.28	1	N.D.	0.25	1	N.D.	3.14	1	N.D.
311	PROZ	Vitamin K-dependent protein Z	44.7	134.9	4	12	0.25	1	N.D.	0.82	2	37.39	0.61	1	N.D.
312	PRSS8	Prostasin	36.4	161.6	2	11.7	N.D.	N.D.	N.D.	N.D.	N.D.	N.D.	0.42	1	N.D.
313	PSCA	Prostate stem cell antigen	12.9	85	1	8.1	0.13	1	N.D.	0.29	1	N.D.	0.97	1	N.D.
314	PTGDS	Prostaglandin-H2 D-isomerase	21	1655.1	7	40.5	2.16	3	22.56	0.45	3	22.61	2.45	4	43.45
315	PTPRT	Receptor-type tyrosine-protein phosphatase T	162	31.5	1	0.9	N.D.	N.D.	N.D.	N.D.	N.D.	N.D.	N.D.	N.D.	N.D.
316	PVR	Poliovirus receptor	45.3	93.1	3	7.9	N.D.	N.D.	N.D.	0.3	1	N.D.	N.D.	N.D.	N.D.
317	PVRL2	Poliovirus receptor-related protein 2	57.7	73.8	1	1.5	N.D.	N.D.	N.D.	N.D.	N.D.	N.D.	N.D.	N.D.	N.D.
318	PVRL4	Poliovirus receptor-related protein 4	55.4	62	2	3.5	N.D.	N.D.	N.D.	0.97	1	N.D.	N.D.	N.D.	N.D.
319	RAB1A	Ras-related protein Rab-1A	22.7	55	1	5.4	N.D.	N.D.	N.D.	0.43	1	N.D.	N.D.	N.D.	N.D.
320	RAI14	Ankycorbin	110	28.2	1	0.7	N.D.	N.D.	N.D.	N.D.	N.D.	N.D.	N.D.	N.D.	N.D.
321	REG1A	Lithostathine-1-alpha	18.7	1389.6	6	53.6	0.67	5	54.23	0.44	3	21.05	2.16	4	67.38
322	RET4	Retinol-binding protein 4	23	3527.4	13	71.6	1.91	7	114.28	1.1	6	169.25	1.31	6	34.28
323	RETN	Resistin	11.4	56	1	10.2	N.D.	N.D.	N.D.	0.45	1	N.D.	0.64	1	N.D.
324	RN220	E3 ubiquitin-protein ligase RNF220	62.7	119.1	1	1.8	0.24	1	N.D.	0.06	1	N.D.	6.36	1	N.D.

325	RNAS2	Non-secretory ribonuclease	18.3	1190.4	4	19.9	0.37	4	55.51	0.25	3	26.28	4.67	2	1.94
326	ROBO4	Roundabout homolog 4	107.4	488.7	7	10	0.23	1	N.D.	0.7	1	N.D.	0.54	3	14.64
327	RS27A	Ubiquitin-40S ribosomal protein S27a	18	794.9	5	34	1.3	4	56.32	0.6	3	57.65	2.04	3	13.54
328	S100P	Protein S100-P	10.4	238	1	13.7	N.D.	N.D.	N.D.	N.D.	N.D.	N.D.	N.D.	N.D.	N.D.
329	S10A6	Protein S100-A6	10.2	213.1	2	16.7	N.D.	N.D.	N.D.	2.72	1	N.D.	0.44	2	19.76
330	S10A7	Protein S100-A7	11.5	184	2	21.8	N.D. (down)	N.D.	N.D.	N.D. (down)	N.D.	N.D.	N.D.	N.D.	N.D.
331	S10A8	Protein S100-A8	10.8	280.6	5	40.9	N.D. (down)	N.D.	N.D.	2.15	3	45.4	0.41	5	70.75
332	S10A9	Protein S100-A9	13.2	2271.7	9	81.6	0.2	4	172.62	1	4	187.36	0.8	5	24.53
333	S10AB	Protein S100-A11	11.7	389.6	3	23.8	N.D.	N.D.	N.D.	0.42	1	N.D.	N.D.	N.D.	N.D.
334	S12A1	Solute carrier family 12 member 1	121.4	51.3	1	0.9	N.D.	N.D.	N.D.	N.D.	N.D.	N.D.	N.D.	N.D.	N.D.
335	SAP	Proactivator polypeptide	58.1	3166.2	14	21.2	0.39	8	119.79	0.32	9	182.76	1.9	8	69.53
336	SAP3	Ganglioside GM2 activator	20.8	609.6	5	29.5	1.36	4	111.07	1.31	3	117.2	1.63	4	23.38
337	SCTM1	Secreted and transmembrane protein 1	27	951.3	4	21	0.07	1	N.D.	0.37	3	144.15	0.76	1	N.D.
338	SCUB1	Signal peptide, CUB and EGF-like domain-containing protein 1	107.8	26.3	1	0.8	N.D.	N.D.	N.D.	N.D.	N.D.	N.D.	N.D.	N.D.	N.D.
339	SDCB1	Syntenin-1	32.4	45.8	1	6.4	N.D.	N.D.	N.D.	N.D.	N.D.	N.D.	N.D.	N.D.	N.D.
340	SEMG1	Semenogelin-1	52.1	330.4	10	23.6	N.D.	N.D.	N.D.	N.D.	N.D.	N.D.	N.D.	N.D.	N.D.
341	SEMG2	Semenogelin-2	65.4	186.6	3	5.3	N.D.	N.D.	N.D.	N.D.	N.D.	N.D.	N.D.	N.D.	N.D.
342	SH3L3	SH3 domain-binding glutamic acid-rich-like protein 3	10.4	945.5	4	66.7	0.17	2	331.58	1.85	2	72.6	0.43	3	22.84
343	SHSA5	Protein shisa-5	25.6	175.1	2	14.6	1.72	2	4.75	N.D. (down)	N.D.	N.D.	N.D.	N.D.	N.D.
344	SIAE	Sialate O-acetylesterase	58.3	67.5	2	4.2	N.D.	N.D.	N.D.	N.D.	N.D.	N.D.	0.59	2	7.72
345	SIRBL	Signal-regulatory protein beta-1 isoform 3	43.3	70.3	3	7.8	N.D.	N.D.	N.D.	N.D.	N.D.	N.D.	N.D.	N.D.	N.D.

346	SLIK1	SLIT and NTRK-like protein 1	77.7	45	1	1.1	N.D.	N.D.	N.D.	N.D.	N.D.	N.D.	N.D.	N.D.	N.D.
347	SLUR1	Secreted Ly-6/uPAR-related protein 1	11.2	842.4	2	52.4	0.34	1	N.D.	0.11	1	N.D.	3.43	1	N.D.
348	SMBT2	Scm-like with four MBT domains protein 2	100.5	33.4	1	1	N.D.	N.D.	N.D.	N.D.	N.D.	N.D.	N.D.	N.D.	N.D.
349	SODC	Superoxide dismutase [Cu-Zn]	15.9	458.8	2	23.4	N.D.	N.D.	N.D.	N.D.	N.D.	N.D.	1.08	1	N.D.
350	SPA12	Serpin A12	47.1	27.1	1	3.1	N.D.	N.D.	N.D.	N.D.	N.D.	N.D.	N.D.	N.D.	N.D.
351	SPB3	Serpin B3	44.5	303.9	8	24.6	N.D.	N.D.	N.D.	N.D.	N.D.	N.D.	1.42	1	N.D.
352	SPN90	NCK-interacting protein with SH3 domain	78.9	27.4	1	1.4	N.D.	N.D.	N.D.	N.D.	N.D.	N.D.	0.56	1	N.D.
353	SPR1A	Cornifin-A	9.9	25.6	1	9	N.D.	N.D.	N.D.	N.D.	N.D.	N.D.	N.D.	N.D.	N.D.
354	SPRR3	Small proline-rich protein 3	18.1	925.2	8	52.1	2.34	3	45.17	N.D. (down)	N.D.	N.D.	5.67	1	N.D.
355	SRC8	Src substrate cortactin	61.5	56.1	1	2.2	N.D.	N.D.	N.D.	N.D.	N.D.	N.D.	N.D.	N.D.	N.D.
356	STK36	Serine/threonine-protein kinase 36	143.9	29.9	1	0.6	1.97	1	N.D.	1.85	1	N.D.	1.15	1	N.D.
357	SUCB1	Succinyl-CoA ligase [ADP-forming] subunit beta, mitochondrial	50.3	49.8	1	1.7	N.D.	N.D.	N.D.	0.75	1	N.D.	0.74	1	N.D.
358	SYNP2	Synaptopodin-2	117.4	27	1	0.7	0.36	1	N.D.	2.89	1	N.D.	0.21	1	N.D.
359	SZT2	Protein SZT2	377.8	30.9	1	0.3	4.52	1	N.D.	N.D.	N.D.	N.D.	N.D.	N.D.	N.D.
360	TENA	Tenascin	240.7	39.2	1	0.4	N.D.	N.D.	N.D.	N.D.	N.D.	N.D.	N.D.	N.D.	N.D.
361	TENX	Tenascin-X	464	50.1	2	0.5	N.D.	N.D.	N.D.	0.4	1	N.D.	1.1	1	N.D.
362	TETN	Tetranectin	22.5	951.8	5	31.2	0.16	2	21.55	0.61	2	43.34	0.48	3	34.21
363	TFF1	Trefoil factor 1	9.1	148	2	29.8	2.68	1	N.D.	7.45	1	N.D.	N.D.	N.D.	N.D.
364	TFF2	Trefoil factor 2	14.3	738.3	4	49.6	1.35	2	29.08	0.6	2	62.51	5.58	2	7.31
365	THBG	Thyroxine-binding globulin	46.3	790.8	9	25.1	0.81	4	164.46	0.84	1	N.D.	2.21	1	N.D.
366	THIO	Thioredoxin	11.7	189.8	2	21	0.59	1	N.D.	0.35	1	N.D.	N.D.	N.D.	N.D.
367	THRB	Prothrombin	70	3737	12	19.8	0.69	11	32.71	0.24	8	77.08	5.81	3	10.86
368	THY1	Thy-1 membrane	17.9	299.4	2	18	0.49	2	6.75	0.32	2	21.57	1.47	2	4.83

		glycoprotein												
369	TINAL	Tubulointerstitial nephritis antigen-like	52.4	66	1	2.1	N.D.	N.D.	N.D.	N.D.	N.D.	N.D.	N.D.	N.D.	N.D.
370	TNIP3	TNFAIP3-interacting protein 3	38.9	45	1	3.1	N.D.	N.D.	N.D.	N.D.	N.D.	N.D.	1.52	1	N.D.
371	TNR16	Tumor necrosis factor receptor superfamily member 16	45.2	523.5	3	9.4	1.1	2	16.82	N.D. (down)	N.D.	N.D.	N.D. (up)	N.D.	N.D.
372	TNR1B	Tumor necrosis factor receptor superfamily member 1B	48.3	73.1	1	2.8	N.D.	N.D.	N.D.	N.D.	N.D.	N.D.	N.D.	N.D.	N.D.
373	TPIS	Triosephosphate isomerase	30.8	130.3	2	8.7	N.D.	N.D.	N.D.	N.D.	N.D.	N.D.	1.78	1	N.D.
374	TPM1	Tropomyosin alpha-1 chain	32.7	58.4	1	3.5	N.D.	N.D.	N.D.	N.D.	N.D.	N.D.	N.D.	N.D.	N.D.
375	TR19L	Tumor necrosis factor receptor superfamily member 19L	46.1	102	1	4.4	N.D.	N.D.	N.D.	N.D.	N.D.	N.D.	N.D.	N.D.	N.D.
376	TRFE	Serotransferrin	77	28280	70	75.8	15.36	31	75.14	1.68	30	67.45	3.67	39	44
377	TRFM	Melanotransferrin	80.2	56	2	2.7	N.D.	N.D.	N.D.	N.D.	N.D.	N.D.	N.D.	N.D.	N.D.
378	TSN1	Tetraspanin-1	26.3	267.9	1	5.4	0.15	1	N.D.	0.42	1	N.D.	N.D.	N.D.	N.D.
379	TTHY	Transthyretin	15.9	1368.2	6	63.9	2.62	5	23.12	0.7	4	15.55	2.45	3	26.38
380	TWSG1	Twisted gastrulation protein homolog 1	25	166.6	3	15.2	0.38	1	N.D.	0.26	1	N.D.	1.24	1	N.D.
381	UBR4	E3 ubiquitin-protein ligase UBR4	573.5	27	1	0.3	N.D.	N.D.	N.D.	N.D.	N.D.	N.D.	N.D.	N.D.	N.D.
382	UFO	Tyrosine-protein kinase receptor UFO	98.3	1478.7	7	11.7	0.45	4	53.52	1.21	4	55.44	0.74	5	22.32
383	UROM	Uromodulin	69.7	11292.5	30	45	0.19	23	74.65	1.28	25	64.89	0.24	23	31.55
384	UTER	Uteroglobin	10	1050.3	5	41.8	N.D.	N.D.	N.D.	N.D.	N.D.	N.D.	1.18	2	5.97
385	VASN	Vasorin	71.7	864.6	5	9.7	0.43	3	47.76	0.7	3	48.26	1.15	4	61.85
386	VCAM1	Vascular cell adhesion protein 1	81.2	857.6	9	13.9	0.85	2	34.66	1.61	3	15.93	0.86	5	9.32
387	VIME	Vimentin	53.6	68.9	3	6.2	N.D.	N.D.	N.D.	N.D.	N.D.	N.D.	N.D.	N.D.	N.D.
388	VMO1	Vitelline membrane outer	21.5	1936.2	7	49.5	0.22	2	15.94	0.98	4	68.1	0.86	4	27.7

		layer protein 1 homolog													
389	VNN1	Pantetheinase	57	94.5	1	2.9	N.D.	N.D.	N.D.	N.D.	N.D.	N.D.	N.D.	N.D.	N.D.
390	VTDB	Vitamin D-binding protein	52.9	4562.9	22	56.3	2.87	13	47.35	1.25	10	51.98	1.95	12	25.89
391	VTNC	Vitronectin GN=VTN PE=1 SV=1	54.3	248.7	2	5.2	0.57	1	N.D.	N.D.	N.D.	N.D.	N.D.	N.D.	N.D.
392	WFDC2	WAP four-disulfide core domain protein 2	13	156.4	2	25.8	N.D.	N.D.	N.D.	N.D.	N.D.	N.D.	N.D.	N.D.	N.D.
393	WISP2	WNT1-inducible-signaling pathway protein 2	26.8	451.8	1	11.2	0.62	1	N.D.	1.3	1	N.D.	1.12	1	N.D.
394	YD002	Uncharacterized protein FLJ44066	104.4	95.1	1	0.9	0.55	1	N.D.	0.39	1	N.D.	3.2	1	N.D.
395	YLPM1	YLP motif-containing protein 1	219.8	30.6	1	0.5	N.D.	N.D.	N.D.	N.D.	N.D.	N.D.	N.D.	N.D.	N.D.
396	ZA2G	Zinc-alpha-2-glycoprotein	34.2	9555.4	27	57.7	6.83	19	165.48	3.27	19	136.41	1.39	20	55.59

Table S2-1. Seventeen proteins were up-regulated according to the results of isobaric tags for relative and absolute quantification.

							(WDM-NP: Healthy)			(DM-WNP: Healthy)			(DM-NP: DM-WNP Healthy)		
NO.	Accession	Protein	MW (kDa)	Scores	Qualitative peptides	SC (%)	iTRAQ Median	Quantitative peptides	CV (%)	iTRAQ Median	Quantitative peptides	CV (%)	iTRAQ Median	Quantitative peptides	CV (%)
1	A1AG1	Alpha-1-acid glycoprotein 1	23.5	801.5	15	31.8	7.94	10	86.17	0.73	10	50.55	4	9	47.17
2	A1AG2	Alpha-1-acid glycoprotein 2	23.6	356.7	9	24.9	3.27	5	156.47	0.63	5	30.28	4.37	4	69.59
3	A1AT	Alpha-1-antitrypsin	46.7	2100.8	36	44	7.29	20	95.11	0.67	14	53.67	3.05	16	55.02
4	A1BG	Alpha-1B-glycoprotein	54.2	1220.7	21	44.4	2.5	6	33.85	1.06	5	22.97	1.14	5	54.03
5	B2MG	Beta-2-microglobulin	13.7	178.1	4	19.3	1.5	4	90.85	0.95	4	41.36	1.42	4	17.18
6	HEMO	Hemopexin	51.6	129.5	3	10	1.62	1	N.D.	1.08	1	N.D.	N.D.	N.D.	N.D.
7	CERU	Ceruloplasmin	122.1	693.2	12	15	1.87	4	28.26	1.27	4	25.3	1.72	5	29.68
8	SAP3	Ganglioside GM2 activator	20.8	285.2	4	7.1	0.45	3	57.74	0.27	3	33.69	3.04	2	16.75
9	HPT	Haptoglobin	45.2	723.3	13	26.6	3.42	10	67.54	1.11	9	39.42	1.3	7	18.55
10	IGHA1	Ig alpha-1 chain C region	37.6	598.5	12	27.5	1.68	4	47.53	2.16	3	48.94	0.79	5	29.4
11	IGHA2	Ig alpha-2 chain C region	36.5	403.6	8	25.9	1.55	3	61.14	2.21	3	44.7	0.79	3	33.56
12	A2GL	Leucine-rich alpha-2-glycoprotein	38.2	742.6	13	39.5	1.22	4	10.19	0.88	4	15.78	1.68	5	45.68
13	PEPA3	Pepsin A-3	41.9	206.4	5	10.8	0.28	2	7.21	0.6	3	33.59	1.93	2	27.55
14	AMBP	Protein AMBP	39	1009.4	19	36.9	1.4	7	187.03	0.41	7	79.48	5.75	6	206.59
15	TRFE	Serotransferrin	77	3991.5	78	48.6	4.71	36	121.14	1.22	36	67.65	2.56	47	66.06
16	TTHY	Transthyretin	15.9	210	3	24.5	3.25	1	N.D.	2.11	1	N.D.	1.29	1	N.D.
17	MYO5B	Unconventional myosin-Vb	213.5	68	2	0.4	2.67	2	33.76	0.93	2	31.39	1.11	2	3.85

Table S2-2. Twenty proteins were down-regulated according to the results from isobaric tags for relative and absolute quantification.

							(WDM-NP: Healthy)			(DM-WNP: Healthy)			(DM-NP: DM-WNP Healthy)		
No	Accession	Protein	MW (kDa)	Scores	Qualitative peptides	SC (%)	iTRAQ Median	Quantitative peptides	CV (%)	iTRAQ Median	Quantitative peptides	CV (%)	iTRAQ Median	Quantitative peptides	CV (%)
1	FETUA	Alpha-2-HS-glyco protein	39.3	644.8	13	34.1	0.57	4	57.87	1.06	4	44.37	0.43	2	23.26
2	AMY1	Alpha-amylase 1	57.7	1443.2	24	40.9	0.35	9	92.92	0.99	10	29.83	0.52	11	80.31
3	AMPN	Aminopeptidase N	109.5	772.4	16	20.1	0.71	2	45.13	1.05	2	22.07	N.D.	N.D.	N.D.
4	APOD	Apolipoprotein D	21.3	1330	27	39.2	0.26	15	70.9	0.21	16	84.85	1.51	4	61.23
5	PGBM	Basement membrane-specific heparan sulfate proteoglycan core protein	468.5	1329.6	18	3.8	0.28	6	64.95	1.13	6	57.01	0.4	7	52.32
6	LG3BP	Galectin-3-binding protein	65.3	439.6	9	17.8	0.29	3	28.3	0.76	3	33.46	0.71	1	N.D.
7	CD44	CD44 antigen	81.5	296.5	6	4.6	0.64	3	60.04	0.21	3	110.89	1.51	3	49.43
8	CD59	CD59 glycoprotein	14.2	118.4	3	15.6	0.66	2	39.46	0.35	2	74.17	1.57	2	34.01
9	DIAC	Di-N-acetylchitobiase	43.7	105.6	2	7.8	0.38	2	37.82	0.53	1	N.D.	0.67	2	0.28
10	SAP3	Ganglioside GM2 activator	20.8	285.2	4	7.1	0.45	3	57.74	0.27	3	33.69	3.04	2	16.75
11	IGHG1	Ig gamma-1 chain C region	36.1	306.6	5	14.8	0.16	2	32.99	0.59	2	45.66	0.67	3	47.43
12	KV204	Ig kappa chain V-II region TEW	12.3	290.9	4	38.9	0.7	2	47.46	1.08	2	8.32	0.48	2	4.79
13	KLK1	Kallikrein-1	28.9	322	5	17.9	0.28	1	N.D.	0.45	1	N.D.	0.86	2	23.8
14	K1C10	Keratin, type I cytoskeletal 10	58.8	690.2	10	16.8	0.3	2	3.84	0.63	1	N.D.	0.68	2	45.93
15	KNG1	Kininogen-1	71.9	1705.2	28	14.1	0.43	11	45.71	0.56	11	63.61	0.55	7	40.93
16	LYAG	Lysosomal alpha-glucosidase	105.3	1172.4	22	27.1	0.21	4	182.56	0.69	4	19.08	0.48	5	18.08

17	AMYP	Pancreatic alpha-amylase	57.7	1687.4	27	46	0.27	9	71.93	1.03	11	37.92	0.49	12	86.7
18	S10A9	Protein S100-A9	13.2	545.1	9	74.6	0.12	3	46.76	0.58	4	109.97	0.72	3	13.47
19	UROM	Uromodulin	69.7	2930.6	51	30.6	0.22	20	84.09	1.03	21	84.45	0.23	18	61.96
20	LMAN2	Vesicular integral-membrane protein VIP36	40.2	659	11	28.7	0.37	2	97.65	0.74	3	15.12	0.86	2	10.69

Table S2-3. Forty-nine proteins were up-regulated according to the results of label-free quantitative proteomics.

							(WDM-NP: Healthy)			(DM-WNP: Healthy)			(DM-NP: DM-WNP Healthy)		
No.	Accession	Protein	MW (kDa)	Scores	Qualitative peptides	SC (%)	LF Median	Quantitative peptides	CV (%)	LF Median	Quantitative peptides	CV (%)	LF Median	Quantitative peptides	CV (%)
1	A1AG1	Alpha-1-acid glycoprotein 1	23.5	7130.3	19	52.7	9.55	7	116.83	0.7	5	38	6.3	6	13.04
2	A1AG2	Alpha-1-acid glycoprotein 2	23.6	4666.1	13	51.7	8.73	7	69.92	0.74	6	44.73	5.26	7	28.24
3	AACT	Alpha-1-antichymotrypsin	47.6	3135.7	16	38.5	2.84	8	44.77	0.84	7	28.75	3.11	9	24.6
4	A1AT	Alpha-1-antitrypsin	46.7	9142.6	37	65.1	10.49	16	42.81	0.54	13	63.87	5.18	14	42.85
5	A1BG	Alpha-1B-glycoprotein	54.2	3834.7	17	51.7	5.74	9	87.28	2.1	9	69.43	1.54	8	14.89
6	FETUA	Alpha-2-HS-glycoprotein	39.3	2918.8	8	26.2	2.17	6	156.39	1.93	7	214.08	0.87	5	37.78
7	ANT3	Antithrombin-III	52.6	849.3	12	30	2.77	3	45.65	N.D. (down)	N.D.	N.D.	N.D. (up)	N.D.	N.D.
8	APOA1	Apolipoprotein A-I	30.8	1183.9	10	40.8	1.67	5	21.24	1.78	5	132.16	1.03	3	109.84
9	APOD	Apolipoprotein D	21.3	3823.4	10	34.9	0.54	8	116.19	0.45	7	157.4	1.79	8	30.16
10	B2MG	Beta-2-microglobulin	13.7	1058.5	9	60.5	2.24	2	7.2	0.36	1	N.D.	1.62	2	19.02
11	CBPQ	Carboxypeptidase Q	51.9	417.4	6	15.3	3.18	1	N.D.	1.2	3	31.21	0.91	5	84.44
12	CD44	CD44 antigen	81.5	756.9	3	4.2	1.11	3	30.06	0.4	3	39.63	3.51	3	13.51
13	CD59	CD59 glycoprotein	14.2	1036.6	4	25	2.19	3	98.17	0.66	3	80.25	2.63	3	12.09
14	CERU	Ceruloplasmin	122.1	7050.4	34	40.9	5.18	15	46.62	2.08	11	36.9	1.98	14	18.5
15	CBG	Corticosteroid-binding globulin	45.1	1035.2	8	23	3.24	4	60.74	0.71	2	22.19	3.12	2	20.09
16	CYTA	Cystatin-A	11	396.4	4	53.1	2.68	3	46.15	N.D. (down)	N.D.	N.D.	N.D. (up)	N.D.	N.D.
17	CYTM	Cystatin-M	16.5	42.5	5	10.3	1.91	3	56.87	0.31	1	N.D.	0.96	1	N.D.
18	FBLN1	Fibulin-1	77.2	313.9	3	6.8	0.67	1	N.D.	N.D.	N.D.	N.D.	5.01	1	N.D.

19	GGH	Gamma-glutamyl hydrolase	35.9	681.4	6	24.2	1.47	3	744.82	1.27	4	40.18	0.46	3	32.51
20	SAP3	Ganglioside GM2 activator	20.8	609.6	5	29.5	1.36	4	111.07	1.31	3	117.2	1.63	4	23.38
21	GELS	Gelsolin	85.6	4428.5	15	24.7	1.76	5	79.32	2.87	5	104.34	0.73	9	26.24
22	HPT	Haptoglobin	45.2	4025	26	53.4	9.56	12	87.79	1.79	13	65.41	1.48	13	27.33
23	HBB	Hemoglobin subunit beta	16	705.8	10	82.3	0.49	2	81.98	0.6	1	N.D.	2.28	3	31
24	HEMO	Hemopexin	51.6	2907.6	19	51.5	2.08	12	28.91	0.99	12	63.44	3.08	11	21.52
25	IGHA1	Ig alpha-1 chain C region	37.6	3698.9	14	49.9	2.08	7	58.53	2.54	7	64.85	1.12	8	12.01
26	HV305	Ig heavy chain V-III region BRO	13.2	762.8	2	25	1.05	1	N.D.	2.43	1	N.D.	0.91	2	17.78
27	LV302	Ig lambda chain V-III region LOI	11.9	369.8	2	21.6	3.16	1	N.D.	6.09	1	N.D.	1.71	2	4.84
28	LAC2	Ig lambda-2 chain C regions	11.3	3364.5	5	74.5	1.88	2	7.9	2.18	1	N.D.	0.9	2	47.04
29	IGLL5	Immunoglobulin lambda-like polypeptide 5	23	3224.7	6	40.7	1.88	2	7.9	2.18	1	N.D.	1.02	3	42.58
30	K1C9	Keratin, type I cytoskeletal 9	62	5105.4	27	55.5	1.78	8	78.03	2.37	6	38.28	0.67	9	50.18
31	K2C1B	Keratin, type II cytoskeletal 1b	61.9	651.1	5	8.3	1.39	2	4.09	1.67	2	16.5	0.61	3	29.18
32	A2GL	Leucine-rich alpha-2-glycoprotein	38.2	3291.3	12	37.2	2.25	7	45.85	1.6	6	53.49	3.18	5	49.87
33	REG1A	Lithostathine-1-alpha	18.7	1389.6	6	53.6	0.67	5	54.23	0.44	3	21.05	2.16	4	67.38
34	LYVE1	Lymphatic vessel endothelial hyaluronic acid receptor 1	35.2	606.3	5	14.3	4.58	3	113.87	0.66	2	26.72	2.41	4	29.59
35	OSTP	Osteopontin	35.4	1571.5	12	46.2	4.83	3	40.27	N.D. (down)	N.D.	N.D.	N.D. (up)	N.D.	N.D.
36	PEPA3	Pepsin A-3	41.9	169.7	4	6.7	0.03	1	N.D.	0.37	3	42.18	2.19	2	13.05

37	PI16	Peptidase inhibitor 16	49.4	2738.5	9	27.6	1.53	4	16.95	1.23	4	16.68	1.34	6	28.8
38	PTGDS	Prostaglandin-H2 D-isomerase	21	1655.1	7	40.5	2.16	3	22.56	0.45	3	22.61	2.45	4	43.45
39	AMBP	Protein AMBP	39	16156.4	27	52	2.2	20	138.75	0.54	15	88.25	3.2	19	317.4
40	THRB	Prothrombin	70	3737	12	19.8	0.69	11	32.71	0.24	8	77.08	5.81	3	10.86
41	RET4	Retinol-binding protein 4	23	3527.4	13	71.6	1.91	7	114.28	1.1	6	169.25	1.31	6	34.28
42	TRFE	Serotransferrin	77	28280	70	75.8	15.36	31	75.14	1.68	30	67.45	3.67	39	44
43	ALBU	Serum albumin	69.3	34933.4	71	78	2.77	38	164.85	4.38	34	127.2	0.62	52	50.69
44	SPRR3	Small proline-rich protein 3	18.1	925.2	8	52.1	2.34	3	45.17	N.D. (down)	N.D.	N.D.	5.67	1	N.D.
45	TTHY	Transthyretin	15.9	1368.2	6	63.9	2.62	5	23.12	0.7	4	15.55	2.45	3	26.38
46	TFF2	Trefoil factor 2	14.3	738.3	4	49.6	1.35	2	29.08	0.6	2	62.51	5.58	2	7.31
47	RS27A	Ubiquitin-40S ribosomal protein S27a	18	794.9	5	34	1.3	4	56.32	0.6	3	57.65	2.04	3	13.54
48	VTDB	Vitamin D-binding protein	52.9	4562.9	22	56.3	2.87	13	47.35	1.25	10	51.98	1.95	12	25.89
49	ZA2G	Zinc-alpha-2-glycoprotein	34.2	9555.4	27	57.7	6.83	19	165.48	3.27	19	136.41	1.39	20	55.59

Table S2-4. Eighty-four proteins were down-regulated according to the results of label-free quantitative proteomics.

							(WDM-NP: Healthy)			(DM-WNP: Healthy)			(DM-NP: DM-WNP Healthy)		
No	Accession	Protein	MW (kDa)	Scores	Qualitative peptides	SC (%)	LF Median	Quantitative peptides	CV (%)	LF Media n	Quantitative peptides	CV (%)	LF Median	Quantitative peptides	CV (%)
1	ACTB	Actin, cytoplasmic 1	41.7	1388.3	13	38.4	0.29	5	44.36	0.65	5	39.4	1.31	5	48.05
2	ACTC	Actin, alpha cardiac muscle 1	42	757.9	8	19.9	0.3	2	51.89	0.51	4	22.87	1.32	4	54.1
3	AMY1	Alpha-amylase 1	57.7	5615.3	23	50.3	0.35	10	81.73	1.32	17	125.6	0.38	13	25.77
4	AMY2B	Alpha-amylase 2B	57.7	5373.5	23	49.9	0.35	10	81.73	1.39	16	127.65	0.39	12	25.72
5	ANAG	Alpha-N-acetyl glucosaminidase	82.2	1347.4	14	26.1	0.25	3	39.66	0.97	10	26.56	0.71	7	16.94
6	AMPN	Aminopeptidase N	109.5	1270.4	21	29.3	0.19	4	49.14	0.51	10	45.23	1.13	10	22.66
7	APOD	Apolipoprotein D	21.3	3823.4	10	34.9	0.54	8	116.19	0.45	7	157.4	1.79	8	30.16
8	PGBM	Basement membrane-specific heparan sulfate proteoglycan core protein	468.5	4377.4	16	4.6	0.25	8	48.93	1.29	9	46.98	0.53	10	21.74
9	APOH	Beta-2-glycoprotein 1	38.3	781	6	31	0.29	4	24.03	0.35	5	28.86	0.97	4	9.21
10	BGAL	Beta-galactosidase	76	662.1	14	30.6	N.D. (down)	N.D.	N.D.	1.09	5	22.66	0.37	6	25.1
11	CADH2	Cadherin-2	99.7	406.5	9	13.9	0.46	2	0.01	1.13	6	14.18	0.64	4	17.15
12	CALB1	Calbindin	30	87.8	3	12.3	N.D. (down)	N.D.	N.D.	0.34	1	N.D.	0.63	1	N.D.
13	CADM4	Cell adhesion molecule4	42.8	464	6	22.4	0.5	2	7.91	0.75	3	11.9	0.64	3	40.97
14	CO6A1	Collagen alpha-1(VI) chain	108.5	846.9	13	17	0.61	1	N.D.	0.68	8	48.75	0.81	7	12.73
15	C1RL	Complement C1r subcomponent-like protein	53.5	297.4	6	13.8	0.45	3	46.95	0.99	4	87.67	1.04	3	18.3
16	CO4B	Complement C4-B	192.6	561.4	5	4.1	0.31	1	N.D.	1.02	2	50.29	1.08	2	10.95
17	CYTB	Cystatin-B	11.1	433.9	2	33.7	0.38	2	0	0.95	2	20.96	0.54	2	5.86

18	DNAS1	Deoxyribonuclease-1	31.4	1229.1	8	43.6	0.12	2	0.84	0.63	6	61.63	0.52	3	7.43
19	DERM	Dermatopontin	24	499.1	4	21.9	0.46	3	38.52	0.83	3	50.5	1.11	2	6.66
20	DSC2	Desmocollin-2	99.9	551.1	9	17.3	0.51	4	88.8	0.8	5	46.28	0.75	4	26.47
21	FBLN3	EGF-containing fibulin-like extracellular matrix protein 1	54.6	1240.4	12	36.7	0.36	5	33.34	0.53	5	18.71	1.16	6	30.43
22	ENDD1	Endonuclease domain-containing 1 protein	55	398.3	8	18.2	0.44	6	46.24	0.86	6	37.85	0.9	3	15.05
23	CD248	Endosialin	80.8	775.5	10	19.9	0.33	2	70.99	0.85	5	99.38	0.47	4	5.93
24	EPCR	Endothelial protein C receptor	26.7	822.2	3	16.4	0.13	3	38.41	0.29	3	76.69	0.61	2	5.77
25	FBN1	Fibrillin-1	312	508.2	5	2.5	0.44	3	46.41	0.63	1	N.D.	N.D. (up)	N.D.	N.D.
26	FIBA	Fibrinogen alpha chain	94.9	2372.3	7	10.2	0.2	2	18.57	0.53	3	41.29	0.78	3	7.48
27	FBLN2	Fibulin-2	126.5	1122.2	3	3.3	0.18	1	N.D.	0.33	1	N.D.	1.32	1	N.D.
28	LG3BP	Galectin-3-binding protein	65.3	2232.5	12	27.9	0.28	7	46.31	0.86	10	28.2	0.78	9	15.08
29	GPX3	Glutathione peroxidase3	25.6	209.8	3	18.1	0.21	1	N.D.	1.04	2	9.35	0.67	2	5.83
30	HMCN1	Hemicentin-1	613	389.1	5	1.4	0.36	2	32.08	0.73	3	76.08	0.79	2	11.77
31	HBB	Hemoglobin subunit beta	16	705.8	10	82.3	0.49	2	81.98	0.6	1	N.D.	2.28	3	31
32	HAVR2	Hepatitis A virus cellular receptor 2	33.4	248.4	4	13.6	0.32	3	13.11	0.35	4	70.07	1.14	3	6.17
33	ICOSL	ICOS ligand	33.3	444.2	3	14.6	0.32	2	21.64	0.48	2	63.09	1.31	2	8.75
34	IGHG1	Ig gamma-1 chain C region	36.1	1708.7	8	21.8	0.13	3	65.5	0.37	5	46.29	0.81	3	24.36
35	IGHG2	Ig gamma-2 chain C region	35.9	601.2	6	19.6	0.27	3	39.98	0.54	4	10.22	0.9	4	32.88
36	IGHG4	Ig gamma-4 chain C region	35.9	1319	8	22	0.17	3	102.71	0.39	5	51.02	1.07	3	15.74
37	KV204	Ig kappa chain V-II region TEW	12.3	1288.2	3	35.4	0.48	3	24.03	1.67	3	19.25	0.95	3	25.6

38	KV301	Ig kappa chain V-III region B6	11.6	1117.4	2	31.5	0.42	1	N.D.	1.09	2	3.52	0.66	2	0.68
39	KV302	Ig kappa chain V-III region SIE	11.8	2114.5	3	39.4	0.43	2	2.31	1.78	3	39.78	0.63	3	11.74
40	KV401	Ig kappa chain V-IV region (Fragment)	13.4	375	3	29.8	0.41	2	21.16	1.02	2	1.09	1.01	2	8.07
41	KV402	Ig kappa chain V-IV region Len	12.6	1054.5	3	36.8	0.44	3	20.31	1.34	3	40.33	0.81	3	32.77
42	IGJ	Immunoglobulin J chain	18.1	458.1	5	28.3	0.4	2	51.7	1.85	3	61.93	0.75	3	5.68
43	KLK1	Kallikrein-1	28.9	1180.7	7	42	0.23	3	14.84	0.6	3	14.81	0.65	4	59.48
44	K1C13	Keratin, type I cytoskeletal 13	49.6	1129.4	19	31.4	0.15	5	134.18	0.44	11	90.9	0.35	1	N.D.
45	K1C14	Keratin, type I cytoskeletal 14	51.5	846.2	10	17.4	0.27	3	73.47	0.5	4	68.98	0.82	3	52.45
46	K22E	Keratin, type II cytoskeletal 2 epidermal	65.4	2772.6	22	34.3	0.84	8	31.31	0.5	8	79.11	1.01	7	57.44
47	K2C5	Keratin, type II cytoskeletal 5	62.3	1091.8	13	18.8	0.33	3	50.09	0.45	4	18.73	0.75	2	66.07
48	K2C6A	Keratin, type II cytoskeletal 6A	60	1198.8	14	22.2	0.3	5	77.86	0.31	5	62.51	1.03	5	73.41
49	KNG1	Kininogen-1	71.9	7303	27	34.6	0.29	14	21.11	0.64	16	35.56	0.66	15	23.64
50	REG1A	Lithostathine-1-alpha	18.7	1389.6	6	53.6	0.67	5	54.23	0.44	3	21.05	2.16	4	67.38
51	FCG3A	Low affinity immunoglobulin gamma Fc region receptor III-A	29.1	254.5	3	10.6	0.1	1	N.D.	0.44	2	171.53	0.89	2	27.52
52	LRP2	Low-density lipoprotein receptor-related protein2	521.6	1360.1	15	3.5	0.42	6	55.39	0.95	8	48.08	0.75	7	50.5
53	LYAG	Lysosomal alpha-glucosidase	105.3	2931.4	20	31.4	0.31	8	46.65	1	16	70.69	0.46	10	36.74
54	CSF1	Macrophage colony -stimulating factor 1	60.1	471.5	4	7.9	0.21	3	22.73	0.23	2	7.4	1.65	1	N.D.
55	MGA	Maltase-glucoamylase, intestinal	209.7	518.2	14	9.7	0.39	5	51.3	1.13	6	49.64	0.49	8	28.2

56	MASP2	Mannan-binding lectin serine protease 2	75.7	2054.7	7	12.2	0.45	5	59.85	1.83	6	111.2	0.57	5	75.08
57	MIEN1	Migration and invasion enhancer 1	12.4	348.6	3	38.3	0.28	1	N.D.	0.96	2	3.79	0.77	2	18.33
58	MUC1	Mucin-1	122	289.3	4	3.8	N.D.	N.D.	N.D.	0.56	2	52.87	1.28	2	13.07
59	PGRP2	N-acetylmuramoyl-L-alanine amidase	62.2	132.4	3	8.9	0.51	1	N.D.	1.09	2	5.75	N.D.	N.D.	N.D.
60	NEGR1	Neuronal growth regulator 1	38.7	307.2	2	9.3	0.44	2	0.6	0.5	2	17.39	1.52	2	18.62
61	NID1	Nidogen-1	136.3	991.2	7	11.4	0.18	1	N.D.	0.75	2	12.01	0.79	3	13.52
62	RNAS2	Non-secretory ribonuclease	18.3	1190.4	4	19.9	0.37	4	55.51	0.25	3	26.28	4.67	2	1.94
63	NTF2	Nuclear transport factor2	14.5	332.8	5	69.3	0.16	2	13.54	0.51	3	9.04	1.29	3	8.7
64	AMYP	Pancreatic alpha-amylase	57.7	5796.9	25	61.3	0.3	11	83.27	1.34	17	124.78	0.38	13	26.82
65	PEPA3	Pepsin A-3	41.9	169.7	4	6.7	0.03	1	N.D.	0.37	3	42.18	2.19	2	13.05
66	IC1	Plasma protease C1 inhibitor	55.1	657.9	6	12.2	0.35	4	92.27	0.4	4	72.75	1.67	4	8.12
67	PIGR	Polymeric immunoglobulin receptor	83.2	2324.8	21	34.2	0.39	6	44.25	1.73	9	88.17	0.54	12	40.67
68	SAP	Proactivator polypeptide	58.1	3166.2	14	21.2	0.39	8	119.79	0.32	9	182.76	1.9	8	69.53
69	EGF	Pro-epidermal growth factor	133.9	349.8	8	9.4	0.12	2	51.56	0.2	2	24.56	1.37	3	35.62
70	PIP	Prolactin-inducible protein	16.6	128.3	3	26	0.27	2	18.34	0.5	1	N.D.	N.D.	N.D.	N.D.
71	PPAP	Prostatic acid phosphatase	44.5	478.6	9	31.9	N.D. (down)	N.D.	N.D.	0.35	4	75.36	2.33	5	13.44
72	S10A8	Protein S100-A8	10.8	280.6	5	40.9	N.D. (down)	N.D.	N.D.	2.15	3	45.4	0.41	5	70.75
73	S10A9	Protein S100-A9	13.2	2271.7	9	81.6	0.2	4	172.62	1	4	187.36	0.8	5	24.53
74	ROBO4	Roundabout homolog 4	107.4	488.7	7	10	0.23	1	N.D.	0.7	1	N.D.	0.54	3	14.64
75	SCTM1	Secreted and transmembrane protein1	27	951.3	4	21	0.07	1	N.D.	0.37	3	144.15	0.76	1	N.D.

76	SH3L3	SH3 domain-binding glutamic acid-rich-like protein 3	10.4	945.5	4	66.7	0.17	2	331.58	1.85	2	72.6	0.43	3	22.84
77	TETN	Tetranectin	22.5	951.8	5	31.2	0.16	2	21.55	0.61	2	43.34	0.48	3	34.21
78	THY1	Thy-1 membrane glycoprotein	17.9	299.4	2	18	0.49	2	6.75	0.32	2	21.57	1.47	2	4.83
79	UFO	Tyrosine-protein kinase receptor UFO	98.3	1478.7	7	11.7	0.45	4	53.52	1.21	4	55.44	0.74	5	22.32
80	UROM	Uromodulin	69.7	11292.5	30	45	0.19	23	74.65	1.28	25	64.89	0.24	23	31.55
81	VASN	Vasorin	71.7	864.6	5	9.7	0.43	3	47.76	0.7	3	48.26	1.15	4	61.85
82	LMAN2	Vesicular integral-membrane protein VIP36	40.2	3150.1	14	39.9	0.15	10	20.5	0.67	12	45.83	1.16	11	17.36
83	PROZ	Vitamin K-dependent protein Z	44.7	134.9	4	12	0.25	1	N.D.	0.82	2	37.39	0.61	1	N.D.
84	VMO1	Vitelline membrane outer layer protein 1 homolog	21.5	1936.2	7	49.5	0.22	2	15.94	0.98	4	68.1	0.86	4	27.7

Table S3. Demographic and clinical characteristics of study participants recruited for the verification of urinary protein markers.

	Healthy (n = 34)	WDM-NP[a] (n = 30)	DM-WNP[b] (n = 89)	DM-NP[c] (n = 55)	Overall *p* value	P^1	P^2	P^3
Age (years)	52.82 (5.65)	65.07 (15.10)	61.10 (10.85)	65.56 (11.39)	<0.001	<0.001	<0.001	0.020
Male (N, %)	19 (55.9%)	14 (46.7%)	48 (53.9%)	26 (47.3%)	0.766			
BMI (kg/m^2)	23.34 (2.78)	24.67 (5.49)	24.97 (3.17)	26.16 (3.90)	0.002	0.257	0.009	0.049
SBP (mmHg)	115.35 (12.35)	128.89 (18.75)	124.89 (11.83)	129.13 (8.51)	<0.001	0.002	<0.001	0.014
DBP (mmHg)	72.24 (9.86)	77.19 (9.02)	76.44 (8.45)	76.42 (7.86)	0.067	0.050	0.020	0.989
FBG (mg/dL)	82.15 (9.34)	100.64 (7.78)	128.10 (22.15)	137.20 (31.74)	<0.001	<0.001	<0.001	0.066
HbA1c (%)	5.59 (0.50)	5.80 (0.52)	6.69 (0.60)	7.02 (0.73)	<0.001	0.145	<0.001	0.003
eGFR (mL/min per 1.73 m^2)	76.94 (11.65)	66.10 (20.71)	93.37 (23.11)	87.82 (24.36)	<0.001	0.037	<0.001	0.172
ACR (mg/g)	5.97 (5.78)	108.93 (67.33)	6.91 (5.98)	97.36 (51.94)	<0.001	<0.001	0.433	<0.001
Age-onset (years)	--	--	52.21 (9.59)	52.22 (10.75)	--	--	--	0.998
Diabetic duration (years)	--	--	8.89 (6.94)	13.35 (9.10)	--	--	--	0.001

Data presented as mean (SD) or N (%). Abbreviation: BMI, body mass index; SBP, systolic blood pressure; DBP, diastolic blood pressure; FBG, fasting blood glucose; HbA1c, hemoglobin A1c; eGFR, estimated glomerular filtration rate; ACR, albumin creatinine ratio. [a] WDM-NP, non-diabetic subjects with nephropathy; [b] DM-WNP, diabetic subjects without nephropathy; [c] DM-NP, diabetic subjects with nephropathy; ^1p value for t test when compared healthy subjects with WDM-NP; ^2p value for t test when compared healthy subjects with DM-WNP; ^3p value for t test when compared DM-WNP with DM-NP.

Table S4. Preliminary results from a nested case-control study to validate two potential urinary biomarkers in the verification phase.

	Early Renal Function Decline [a]		
	**Yes (n=20)**	**No (n=17)**	**P value**
Age (years)	56.65 (9.43)	54.59 (8.56)	0.494
Male (N, %)	11 (47.8%)	12 (52.2%)	0.330
Follow up duration (years)	4.80 (2.19)	3.47 (1.46)	0.035*
DM duration (years)	7.83 (4.43)	9.56 (6.17)	0.464
**At haptoglobin measurement**			
HbA1c (%)	8.34 (2.03)	8.25 (2.22)	0.901
Urine Creatinine (mg/dL)	111.17 (66.13)	126.91 (70.70)	0.668
eGFR (mL/min per 1.73 m^2)	104.45 (25.44)	103.53 (28.12)	0.917
BMI (kg/m^2)	26.26 (5.06)	25.62 (3.20)	0.643
SBP (mmHg)	126.30 (20.11)	132.12 (16.41)	0.536
DBP (mmHg)	70.70 (15.19)	75.53 (11.30)	0.288
**Biomarker concentrations**			
ACR (mg/g)	39.81 (58.75)	38.28 (46.72)	0.930
HCR[b] (ng/mg)	3.21 (1.65)	4.21 (1.22)	0.049*
AMBP_C[b] (pg/mg)	3.25 (0.87)	3.51 (1.02)	0.418
HPX_C (ng/mg)	39.92 (67.00)	51.13 (93.98)	0.676
CST_C (ng/mg)	68.63 (78.81)	67.18 (65.49)	0.952
A1AT_C (ng/mg)	299.21 (371.28)	251.75 (187.79)	0.636

Abbreviation: ERFD, early renal functional decline; DM, diabetes mellitus; eGFR, estimated glomerular filtration rate; BMI, body mass index; SBP, systolic blood pressure; DBP, diastolic blood pressure; ACR, albumin-to-creatinine ratio; HCR, Haptoglobin-to-creatinine ratio; AMBP_C, α-1-microglobulin/bikunin precursor (AMBP)-to-creatinine ratio; HPX_C, Hemopexin-to-creatinine ratio; CST_C, Cystatin C-to-creatinine ratio; A1AT_C, Alpha-1-antitrypsin-to-creatinine ratio; [a]Early renal function decline was defined as more than 3.3 mL/min per 1.73 m^2 decline in the eGFR per year; [b]nature logarithmic transformed.

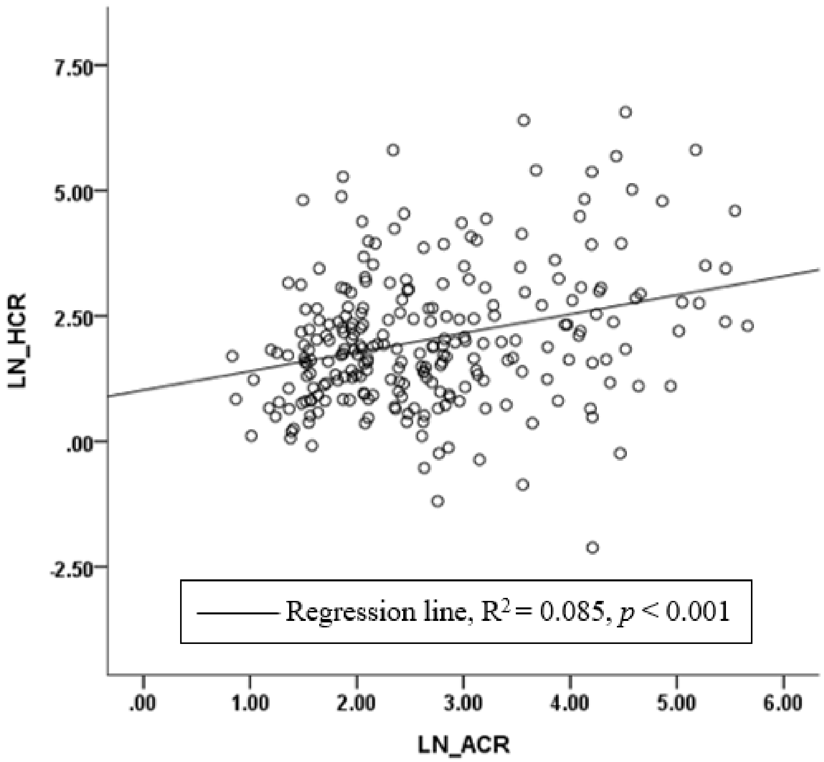

Figure S2. Correlation between HCR and ACR. Abbreviation: ACR, albumin to creatinine ratio; HCR, haptoglobin to creatinine ratio; LN, nature logarithmic transformed; R^2, the Pearson correlation coefficient.
